# Transcriptome analysis of functional differentiation between haploid and diploid cells of *Emiliania huxleyi*, a globally significant photosynthetic calcifying cell

**DOI:** 10.1186/gb-2009-10-10-r114

**Published:** 2009-10-15

**Authors:** Peter von Dassow, Hiroyuki Ogata, Ian Probert, Patrick Wincker, Corinne Da Silva, Stéphane Audic, Jean-Michel Claverie, Colomban de Vargas

**Affiliations:** 1 Evolution du Plancton et PaleOceans, Station Biologique de Roscoff, CNRS UPMC UMR7144, 29682 Roscoff, France; 2 Information Génomique et Structurale, CNRS - UPR2589, Institut de Microbiologie de la Méditerranée, Parc Scientifique de Luminy - 163 Avenue de Luminy - Case 934, FR- 13288, Marseille cedex 09, France; 3 Genoscope, 2 Rue Gaston Crémieux, 91057 Evry, France

## Abstract

**Background:**

Eukaryotes are classified as either haplontic, diplontic, or haplo-diplontic, depending on which ploidy levels undergo mitotic cell division in the life cycle. *Emiliania huxleyi *is one of the most abundant phytoplankton species in the ocean, playing an important role in global carbon fluxes, and represents haptophytes, an enigmatic group of unicellular organisms that diverged early in eukaryotic evolution. This species is haplo-diplontic. Little is known about the haploid cells, but they have been hypothesized to allow persistence of the species between the yearly blooms of diploid cells. We sequenced over 38,000 expressed sequence tags from haploid and diploid *E. huxleyi *normalized cDNA libraries to identify genes involved in important processes specific to each life phase (2N calcification or 1N motility), and to better understand the haploid phase of this prominent haplo-diplontic organism.

**Results:**

The haploid and diploid transcriptomes showed a dramatic differentiation, with approximately 20% greater transcriptome richness in diploid cells than in haploid cells and only ≤ 50% of transcripts estimated to be common between the two phases. The major functional category of transcripts differentiating haploids included signal transduction and motility genes. Diploid-specific transcripts included Ca^2+^, H^+^, and HCO_3_^- ^pumps. Potential factors differentiating the transcriptomes included haploid-specific Myb transcription factor homologs and an unusual diploid-specific histone H4 homolog.

**Conclusions:**

This study permitted the identification of genes likely involved in diploid-specific biomineralization, haploid-specific motility, and transcriptional control. Greater transcriptome richness in diploid cells suggests they may be more versatile for exploiting a diversity of rich environments whereas haploid cells are intrinsically more streamlined.

## Abstract 2

An EST analysis of the phytoplankton *Emiliania huxleyi* reveals genes involved in haploid- and diploid-specific processes and provides insights into environmental adaptation.

## Background

Coccolithophores are unicellular marine phytoplankton that strongly influence carbonate chemistry and sinking carbon fluxes in the modern ocean due to the calcite plates (coccoliths) that are produced in intracellular vacuoles and extruded onto the cell surface [1]. Coccolithophores are members of the Haptophyta [2,3], a basal-branching division of eukaryotes with still uncertain phylogenetic relationships with other major lineages of this domain [4,5]. Intricately patterned coccoliths accumulated in marine sediments over the past 220 million years have left one of the most complete fossil records, providing an exceptional tool for evolutionary reconstruction and biostratigraphic dating [3]. Coccolith calcification also represents a potential source of nanotechnological innovation. Fossil records indicate that *Emiliania huxleyi *arose only approximately 270,000 years ago [6], yet this single morphospecies is now the most abundant and cosmopolitan coccolithophore, seasonally forming massive blooms reaching over 10^7 ^cells l^-1 ^in temperate and sub-polar waters [7]. Many studies are being conducted to determine how the on-going anthropogenic atmospheric CO_2 _increases affect *E. huxleyi *calcification, with conflicting results [8,9]. Because of its environmental prominence and ease of maintenance in laboratory culture, *E. huxleyi *has become the model coccolithophore for physiological, molecular, genomic and environmental studies, and a draft genome assembly of one strain, CCMP1516, is now being analyzed [10]. However, coccolithophorid biology still is in its infancy.

*E. huxleyi *exhibits a haplo-diplontic life cycle, alternating between calcified, non-motile, diploid (2N) cells and non-calcified, motile, haploid (1N) cells, with both phases being capable of unlimited asexual cell division [11,12]. Almost all laboratory and environmental studies on this species have focused only on 2N cells, and lack of information about the ecophysiology and biochemistry of 1N cells represents a large knowledge gap in understanding the biology and evolution of *E. huxleyi *and coccolithophores. More generally, a major question remaining in understanding eukaryotic life cycle evolution is the evolutionary maintenance of haplo-diplontic life cycles in a broad diversity of eukaryotes [13,14], and *E. huxleyi *represents a prominent organism in which new insights might be gained.

*E. huxleyi *1N cells are very distinct from both calcified and non-calcified 2N cells in ultrastructure [12] and ecophysiological properties [15]. 1N cells have two flagella and associated flagellar bases, whereas 2N cells completely lack both flagella and flagellar bases. The coccolith-forming apparatus is present in both calcified and naked-mutants of 2N cells but is absent in 1N cells [7]. 1N cells are also differentiated from 2N cells by formation of particular non-mineralized organic body scales (and thus are not 'naked') [7,11]. 1N cells show different growth preferences relative to 2N cells [16] and do not have the exceptional ability to adapt to high light exhibited by 2N cells [15]. As 1N cells of *E. huxleyi *are not recognizable by classic microscope techniques, little is yet known about their ecological distribution. Recent advances in fluorescent *in situ *hybridization now allow detection of non-calcified *E. huxleyi *cells in the environment [17], although it is still impossible to distinguish 1N cells from non-calcified 2N cells. However, 1N cells of certain other coccolithophore species are recognizable due to the production of distinct holococcolith structures and appear to have a shallower depth distribution and preference for oligotrophic waters compared to 2N cells of the same species [18]. Recently, *E. huxleyi *1N cells were demonstrated to be resistant to the EhV viruses that are lethal to 2N cells and are involved in terminating massive blooms of 2N cells in nature [19]. This suggests that 1N cells might have a crucial role in the long-term maintenance of *E. huxleyi *populations by serving as the link for survival between the yearly 'boom and bust' successions of 2N blooms.

The pronounced differences between 1N and 2N cells suggest a large difference in gene expression between the two sexual stages. In this study, we conducted a comparison of the 1N and 2N transcriptomes in order to: test the prediction that expression patterns are, to a large extent, ploidy level specific; identify a set of core genes expressed in both life cycle phases; identify genes involved in important cellular processes known to be specific to one phase or the other (for example, motility for 1N cells and calcification for 2N cells); provide insights into transcriptional/epigenetic controls on phase-specific gene expression; and provide the basis for the development of molecular tools allowing the detection of 1N cells in nature. For our analysis we selected isogenic cultures originating from strain RCC1216 because strain CCMP1516, from which the genome sequence will be available, has not been observed to produce flagellated 1N cells. Pure clonal 1N cultures (RCC1217) originating from RCC1216 have been stable for several years and can be compared to pure 2N cultures originating from the same genetic background [15,16]. We produced separate normalized cDNA libraries from pure axenic 1N and 2N cultures. Over 19,000 expressed sequence tag (EST) sequences were obtained from each library. Inter-library comparison revealed major compositional differences between the two transcriptomes, and we confirmed the predicted ploidy phase-specific expression for some genes by reverse transcription PCR (RT-PCR).

## Results

### Strain origins and characteristics at time of harvesting

*E. huxleyi *strains RCC1216 (2N) and RCC1217 (1N) were both originally isolated into clonal culture less than 10 years prior to the collection of biological material in this study (Table 1). Repeated analyses of nuclear DNA content by flow cytometry have shown no detectable variation in the DNA contents (the ploidy) of these strains over several years ([20] and unpublished tests performed in 2006 to 2008). Axenic cultures of both 1N and 2N strains were successfully prepared.

**Table 1:** Origins of *Emiliania huxleyi *strains

Strain designation	RCC1216	RCC1217
Strain synonym	TQ26-2N	TQ26-1N
Coccolith morphotype	R	NA
Origin	Tasman Sea, New Zealand Coast	Clonal isolate from RCC1216
Date of isolation	October, 1998	July, 1999
Date axenic cultures prepared, and purity of ploidy type ensured	August-October 2007	August-October 2007
Date of RNA harvest	11-12 November 2007	12-13 December 2007

NA, not applicable.

**Figure 1:**
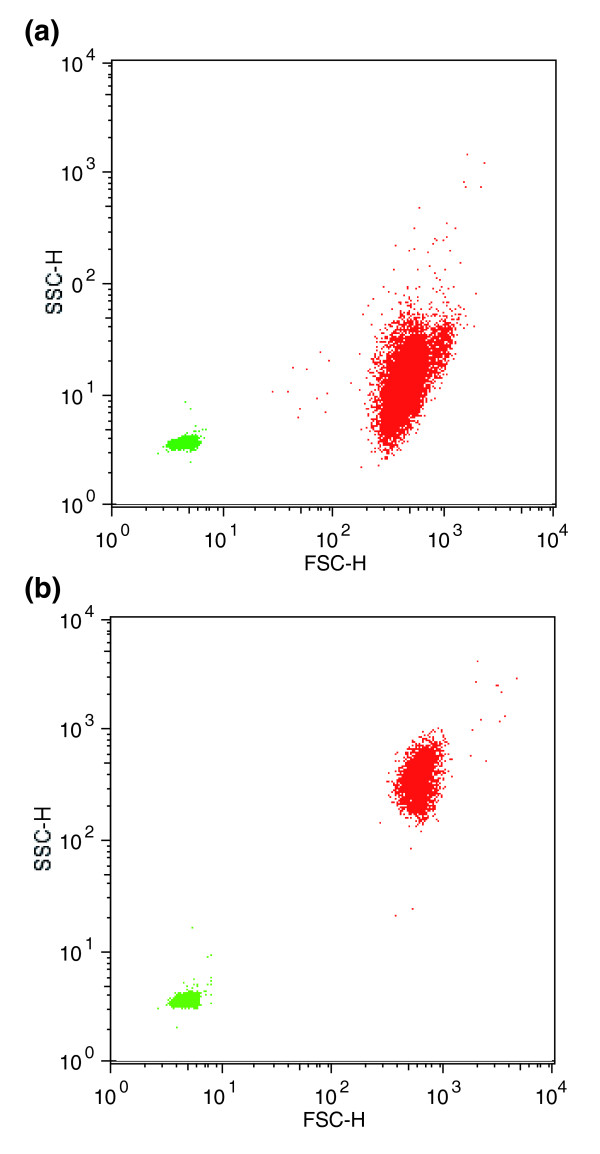
Flow cytometry plot showing conditions of cells in cultures on day of harvesting. **(a) **1N and, **(b) **2N cells (red) were identified by chlorophyll autofluorescence and their forward scatter (FSC) and side scatter (SSC) were compared to 1 μm bead standards (green).

The growth rates of the 2N and 1N cultures used for library construction were 0.843 ± 0.028 day^-1 ^(n = 4) and 0.851 ± 0.004 day^-1 ^(n = 2), respectively. These rates were not significantly different (*P *= 0.70). Two other 1N cultures experienced exposure to continuous light for one or two days prior to harvesting due to a failure of the lighting system. The growth rate of these 1N cultures was 0.893 ± 0.008 day^-1 ^(n = 2). These cultures were not used for library construction but were included in RT-PCR tests. Flow cytometric profiles and microscopic examination taken during harvesting indicated that nearly 100% of 2N cells were highly calcified (indicated by high side scatter) and that no calcified cells were present in the 1N cultures [21] (Figure 1). No motile cells were seen in extensive microscopic examination of 2N cultures over a period of 3 months. 1N cells were highly motile, and displayed prominent phototaxis in culture vessels (not shown).

Both 1N and 2N cultures maintained high photosynthetic efficiency measured by maximum quantum yield of photosytem II (Fv/Fm) throughout the day-night period of harvesting. The Fv/Fm of phased 1N cultures was 0.652 ± 0.009 over the whole 24-h period; it was slightly higher during the dark (0.661 ± 0.003) than during the light period (0.644 ± 0.001; *P *= 9.14 × 10^-5^). The Fv/Fm of 2N cells was 0.675 ± 0.007, with no significant variation between the light and dark periods. These data suggest that both the 1N and 2N cells were maintained in a healthy state throughout the entire period of harvesting.

**Figure 2:**
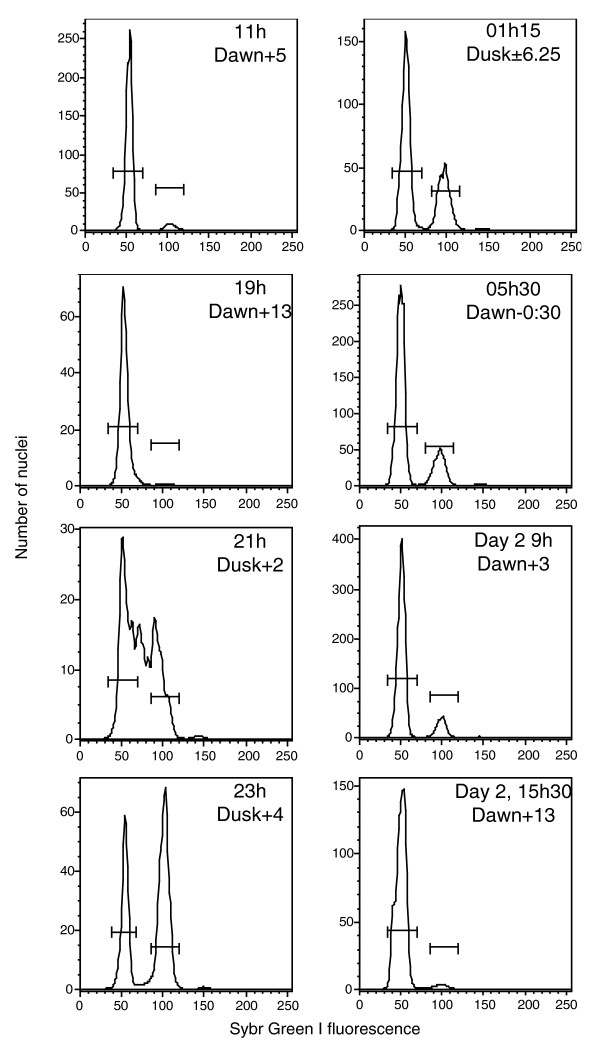
Cell cycle changes during the day-night cycle of harvesting. Example DNA content histograms of nuclear extracts taken from 1N cultures at different times are shown. The time point at 15 h on day 1 is not shown but had a similar distribution to that at 19 h on day 1 and 15 h30 on day 2. RNA was not collected at 15 h30 on day 2, but nuclear extracts (shown here), flow cytometric profiles, and Fv/Fm confirmed cells had returned to the same state after a complete diel cycle. Extracted nuclei were stained with Sybr Green I and analyzed by flow cytometry.

Cell division was phased to the middle of the dark period both in 2N cultures and in the 1N cultures on the correct light-dark cycle (Figure S1 in Additional data file 1). The 1N cultures exposed to continuous light did not show phased cell division. Nuclear extraction from the phased 1N cultures showed that cells remained predominantly in G1 phase throughout the day, entered S phase 1 h after dusk (lights off), and reached the maximum in G2 phase at 3 to 4 h into the dark phase (Figure 2). A small G2 peak was present in the morning hours and disappeared in the late afternoon. These data show that we successfully captured all major changes in the diel and cell cycle of actively growing, physiologically healthy 1N and 2N cells for library construction (below).

### Global characterization of haploid and diploid transcriptomes

#### General features, comparison to existing EST datasets, and analysis of transcriptome complexity and differentiation

High quality total RNA was obtained from eight time points in the diel cycle (Figure S2 in Additional data file 1) and pooled for cDNA construction. We performed two rounds of 5'-end sequencing. In the first round, 9,774 and 9,734 cDNA clones were sequenced from the 1N and 2N libraries, respectively. In the second round, additional 9,758 1N and 9,825 2N clones were selected for sequencing. Altogether our sequencing yielded 19,532 1N and 19,559 2N reads for a total of 39,091 reads (from 39,091 clones). Following quality control, we finally obtained 38,386 high quality EST sequences ≥ 50 nucleotides in length (19,198 for 1N and 19,188 for 2N). The average size of the trimmed ESTs was 582 nucleotides with a maximum of 897 nucleotides (Table 2). Their G+C content (65%) was identical to that observed for ESTs from *E. huxleyi *strain CCMP1516 [22], and was consistent with the high genomic G+C content (approximately 60%) of *E. huxleyi*.

**Table 2:** EST read characteristics

	RCC1217 1N	RCC1216 2N
Number of raw sequences	19,532	19,559
Number of ESTs after trimming, quality control	19,198	19,188
Length of high quality trimmed ESTs, mean ± standard deviation (minimum/maximum)	599.51 ± 143.14 (50/897)	563.55 ± 151.37 (55/866)
%GC	64.49	64.68

Sequence similarity searches between the 1N and 2N EST libraries revealed that only approximately 60% of ESTs in one library were represented in the other library. More precisely, 56 to 59% of 1N ESTs had similar sequences (≥ 95% identity) in the 2N EST library, and 59 to 62% of the 2N ESTs had similar sequences in the 1N EST library, with the range depending on the minimum length of BLAT alignment (100 nucleotides or 50 nucleotides). To qualify this overlap between the 1N and 2N libraries, we constructed two artificial sets of ESTs by first pooling the ESTs from both libraries and then re-dividing them into two sets based on the time of sequencing (that is, the first and the second rounds). Based on the same similarity search criteria, a larger overlap (73 to 79%) was found between the two artificial sets than between the 1N and 2N EST sets. Given the fact that our cDNA libraries were normalized towards uniform sampling of cDNA species, this result already indicates the existence of substantial differences between the 1N and 2N transcriptomes in our culture conditions.

**Figure 3:**
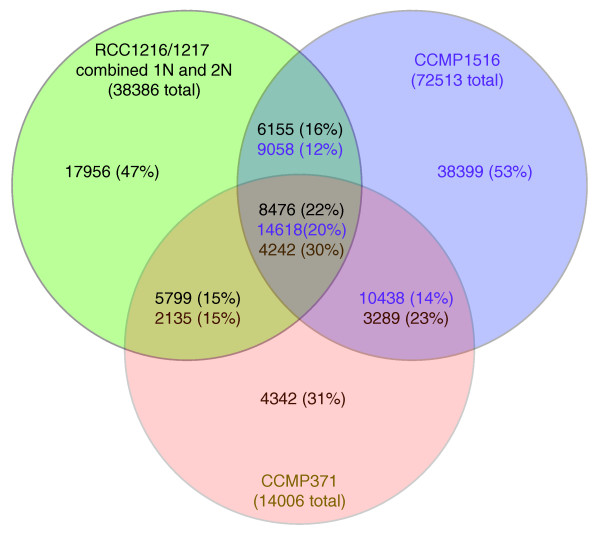
Venn diagram showing the degree of overlap existing *E. huxleyi *EST libraries. Included are the libraries analyzed in this study (1N RCC1217 and 2N RCC1216, combined) and the two other publicly available EST libraries (CCMP 1516 and CCMP371). ESTs were considered matching based on BLAT criteria of an alignment length of ≥ 100 nucleotides and ≥ 95% identity. The degrees of overlap increased only very modestly when the BLAT criteria were relaxed to an alignment length of ≥ 50 nucleotides.

Sequence similarity search further revealed an even smaller overlap between the ESTs from RCC1216/RCC1217 and the ESTs from other diploid strains of different geographic origins (CCMP1516, B morphotype, originating from near the Pacific coast of South America, 72,513 ESTs; CCMP371, originating from the Sargasso Sea, 14,006 ESTs). Only 38% of the RCC1216/RCC1217 ESTs had similar sequences in the ESTs from CCMP1516, and only 37% had similar sequences in the ESTs from CCMP371 (BLAT, identity ≥ 95%, alignment length ≥ 100 nucleotides; Figure 3). Overall, 53% of the RCC1216/RCC1217 ESTs had BLAT matches in these previously determined EST data sets. Larger overlaps were observed for the ESTs from the diploid RCC1216 (47% with CCMP1516 and 45% with CCMP371) than for the haploid RCC1217 strain (37% with CCMP1516 and 36% with CCMP371), consistent with the predominantly diploid nature of the CCMP1516 and CCMP371 strains at the time of EST generation. When the best alignment was considered for each EST, the average sequence identity between strains was close to 100% (that is, 99.7% between RCC1216/RCC1217 and CCMP1516, 99.6% between RCC1216/RCC1217 and CCMP371, and 99.5% between CCMP1516 and CCMP371), being much higher than the similarity cutoff (≥ 95% identity) used in the BLAT searches. The average sequence identity between RCC1216 (2N) and RCC1217 (1N) was 99.9%. Thus, sequence divergence between strains (or alleles) was unlikely to be the major cause of the limited level of overlap between these EST sets. A large fraction of our EST datasets thus likely provides formerly inaccessible information on *E. huxleyi *transcriptomes.

One of the primary objectives of this study was to estimate the extent to which the change in ploidy affects the transcriptome. Therefore, we utilized for the following analyses only the ESTs from RCC1216 (2N) and RCC1217 (1N), originating from cultures of pure ploidy state and identical physiological conditions. The 38,386 ESTs from 1N and 2N libraries were found to represent 16,470 consensus sequences (mini-clusters), which were further grouped into 13,056 clusters (Table 3; Additional data file 2 includes a list of all ESTs with the clusters and mini-clusters to which they are associated and their EMBL accession numbers). Of the 13,056 clusters, only 3,519 (26.9%) were represented by at least one EST from each of the two libraries, thus defining a tentative 'core set' of EST clusters expressed in both cell types. The remaining clusters were exclusively composed of EST(s) from either the 1N (4,368 clusters) or the 2N (5,169 clusters) library; hereafter, we denote these clusters as '1N-unique' and '2N-unique' clusters, respectively. Cluster size (that is, the number of ESTs per cluster) varied from 1 (singletons) up to 43, and displayed a negative exponential rank-size distribution for both libraries (Figure S3 in Additional data file 1). The Shannon diversity indices were found close to the theoretical maximum for both libraries, indicating a high evenness in coverage and successful normalization in our cDNA library construction (Table 4). Crucially, the fact that the rank-size distributions of the two libraries were essentially identical also shows that the normalization process occurred comparably in both libraries (Figure S3 in Additional data file 1).

**Table 3:** EST clusters

	Total	1N and 2N	1N only	2N only
Number of mini-clusters	16,470	3,226	6,002	7,242
Number of mini-clusters (containing ≥ 2 EST reads)	6,444	3,226	1,765	1,453
Number of mini-clusters singletons (only 1 read)	10,026	0	4,237	5,789
Number of clusters	13,056	3,519	4,368	5,169
Number of clusters (≥ 2 EST reads)	6,701	3,519	1,717	1,465
Number of clusters singletons (only 1 read)	6,355	0	2,651	3,704

Clusters were generated from the total pool of 1N (RCC1217) and 2N (RCC1216) ESTs. Clusters represented by EST reads in both libraries (1N and 2N) and clusters with representation in only one library (1N only or 2N only) are also shown.

**Table 4:** Analysis of transcriptome complexity

	RCC1217 1N	RCC1216 2N	Combined libraries
Total clusters	7,887	8,688	13,056
ML estimate of transcriptome richness	10,039	11,988	16,211
Chao1 ± SD (boundaries of 95% CI)	12,840 ± 214 (12,438, 13,278)	15,931 ± 289 (15,385, 16,522)	22,169 ± 314 (21,573, 22,806)
Coverage (%) based on richness estimates	61.4-78.6	54.5-72.5	58.9-80.5
Shannon diversity (maximum possible)	8.66 (8.97)	8.76 (9.06)	9.05 (9.48)

The maximum likelihood (ML) estimate of transcriptome richness was calculated following Claverie [23] using the two separate rounds of EST sequencing. The Chao1 estimator of transcriptome richness and the Shannon diversity index was computed for each library separately and for the combined library using EstimateS with the classic formula for Chao1. The range of estimated coverage was calculated by dividing the number of clusters observed by the two estimates of transcriptome richness. The similarity of content of the 1N and 2N libraries was also determined: the Chao abundance-based estimator of the Jaccard similarity index (accounting for estimated proportions of unseen shared and unique transcripts) was 0.506 ± 0.009, calculated with 200 bootstrap replicates and the upper abundance limit for rare or infrequent transcript species set at 2. The maximum possible Shannon diversity index was calculated as the natural log of the number of clusters.

Interestingly, a larger number of singletons was obtained from the 2N library (3,704 singletons, 19% of 2N ESTs) than from the 1N library (2,651 singletons, 14% of 1N ESTs), suggesting that 2N cells may express more genes (that is, RNA species) than 1N cells. To test this hypothesis, we assessed transcriptome richness (that is, the total number of mRNA species) of 1N and 2N cells using a maximum likelihood (ML) estimate [23] and the Chao1 richness estimator [24]. These estimates indicated that 2N cells express 19 to 24% more genes than 1N cells under the culture conditions in this study, supporting the larger transcriptomic richness for 2N relative to 1N (Table 4). To assess the above-mentioned small overlap between the 1N and 2N EST sets, we computed the abundance-based Jaccard similarity index between the two samples based on our clustering data. This index provides an estimate for the true probability with which two randomly chosen transcripts, one from each of the two libraries, both correspond to genes expressed in both cell types (to take into account that further sampling of each library would likely increase the number of shared clusters because coverage is less than 100%). From our samples, this index was estimated to be 50.6 ± 0.9% and again statistically supports a large transcriptomic difference between the haploid and diploid life cycles.

#### Functional difference between life stages

**Figure 4:**
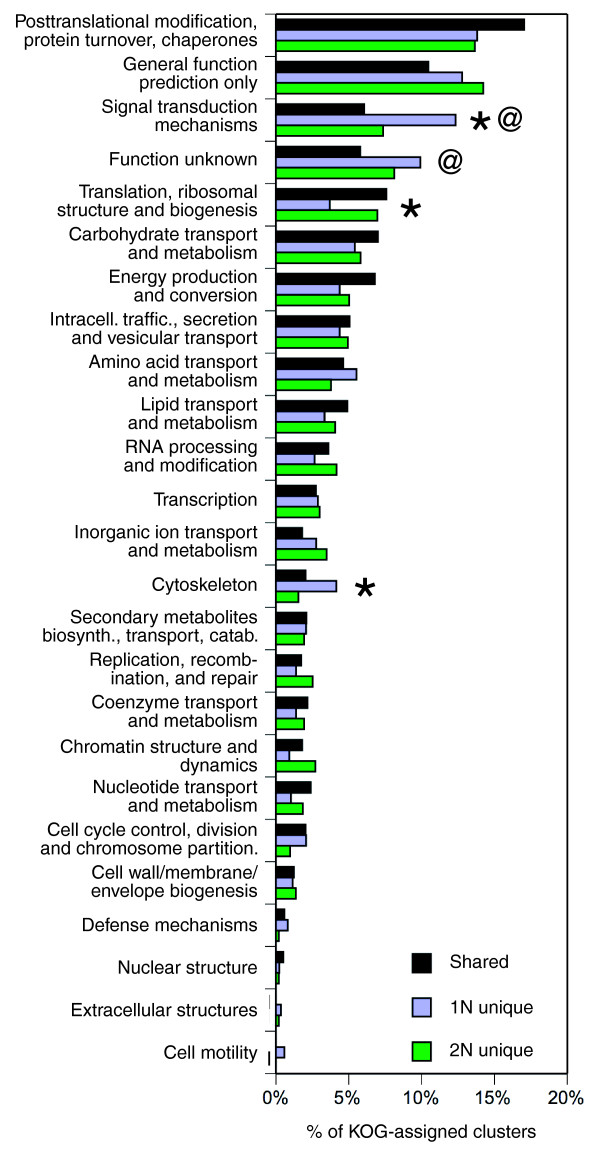
Distribution of clusters and reads by KOG functional class and library. Distributions of clusters over KOG class for clusters shared between the 1N and 2N libraries and clusters unique to each library. Fisher's exact test was used to determine significant differences in the distribution of clusters by KOG class between the 1N-unique and 2N-unique sets (asterisks indicate the KOG classes exhibiting significant differences between the 1N-unique and 2N-unique sets); *P *< 0.002 without correction for multiple tests). The same test was applied to determine differences in the distribution of clusters by KOG class between the set of shared clusters and both 1N-unique and 2N-unique clusters (the at symbol (@) indicates KOG classes exhibiting significant differences between the 1N-unique and shared sets; *P *< 0.002 without correction for multiple tests).

In the NCBI eukarote orthologous group (KOG) database, 3,286 clusters (25.2%) had significant sequence similarity to protein sequence families (Additional data file 3 provides a list of all clusters with their top homologs identified in UniProt, Swiss-Prot, and KOG, and also the number of component mini-clusters and ESTs from each library). Of these KOG-matched clusters, 2,253 were associated with 1N ESTs (1,385 shared core clusters plus 868 1N-unique clusters), and 2,418 were associated with 2N ESTs (1,385 shared core clusters plus 1,033 2N-unique clusters). The distributions of the number of clusters across different KOG functional classes were generally similar among the 1N-unique, the 2N-unique and the shared core clusters, with exceptions in several KOG classes (Figure 4a). The 'signal transduction mechanisms' and 'cytoskeleton' classes were significantly over-represented (12.3% and 4.15%) in the 1N-unique clusters relative to the 2N-unique clusters (7.36% and 1.55%) (*P *< 0.002; Fisher's exact test, without correction for multiple tests). These classes were also less abundant in the shared clusters (6.06% and 2.02%) compared to the 1N-unique clusters (*P *= 3.49 × 10^-7 ^for 'signal transduction mechanisms'; *P *= 0.00395 for 'cytoskeleton'). In contrast, the 'translation, ribosomal structure and biogenesis' class was significantly under-represented (3.69%) in the 1N-unique clusters compared to the 2N-unique (6.97%) and the shared clusters (7.58%). Similar differences were observed when the 1N-unique and 2N-unique sets were further restricted to clusters containing two or more ESTs (Figure S4 in Additional data file 1).

We used Audic and Claverie's method [25] to rank individual EST clusters based on the significance of differential representation in 1N versus 2N libraries. An arbitrarily chosen threshold of *P *< 0.01 provided a list of 220 clusters predicted to be specific to 1N (Additional data file 4) and a list of 110 clusters predicted to be specific to 2N (Additional data file 5). A major caveat is that normalization tends to reduce the confidence in determining differentially expressed genes between cells. As a first step to examine the prediction, we were particularly interested in transcripts that may be effectively absent in one life phase but not the other. Namely, we focused on 198 (90.0%) that are specific and unique to 1N as well as 89 (80.9%) clusters that are specific and unique to 2N, which we termed 'highly 1N-specific' (Tables 5 and 6; Additional data file 4) and 'highly 2N-specific' clusters (Tables 7 and 8; Additional data file 5).

**Table 5:** KOG-assigned EST clusters predicted to be highly 1N-specific based on statistical comparison of libraries

Cluster ID	Number of 1N ESTs	*P*-value	Homolog ID	Homolog description	BLAST
Amino acid transport and metabolism					
GS01965	6	7.8 × 10^-3^	CDO_CAEBR	Cysteine dioxygenase	8 × 10^-19^
GS00820	7	3.9 × 10^-3^	*Q8GYS4_ARATH	Putative uncharacterized protein	5 × 10^-11^
Carbohydrate transport and metabolism					
GS01922	6	7.8 × 10^-3^	AAPC_CENCI	Putative apospory-associated protein C	2 × 10^-25^
Cell cycle control, cell division, chromosome partitioning					
**GS00508**	6	7.8 × 10^-3^	^†^Cyclin_N	Cyclin, N-terminal domain	1 × 10^-09^
Chromatin structure and dynamics					
GS09138	13	6.1 × 10^-5^	H4_OLILU	Histone H4	1 × 10^-38^
Cytoskeleton					
GS00708	6	7.8 × 10^-3^	DYI3_ANTCR	Dynein intermediate chain 3, ciliary	6 × 10^-62^
Function unknown					
GS00091	6	7.8 × 10^-3^	EMAL4_MOUSE	Echinoderm microtubule-associated protein-like 4	4 × 10^-36^
GS02362	7	3.9 × 10^-3^	*A8Q1G0_MALGO	Putative uncharacterized protein	8 × 10^-16^
GS00939	6	7.8 × 10^-3^	* B8BBW9_ORYSI	Putative uncharacterized protein	9 × 10^-08^
General function prediction only					
**GS01285**	6	7.8 × 10^-3^	EHMT2_MOUSE	Histone-lysine N-methyltransferase	3 × 10^-13^
GS08284	8	2.0 × 10^-3^	EI2B_AQUAE	Putative translation initiation factor eIF-2B	4 × 10^-27^
GS00938	7	3.9 × 10^-3^	MORN3_HUMAN	MORN repeat-containing protein 3	4 × 10^-18^
GS00985	6	7.8 × 10^-3^	PTHD2_MOUSE	Patched domain-containing protein 2	2 × 10^-08^
Inorganic ion transport and metabolism					
GS01939	6	7.8 × 10^-3^	AMT12_ARATH	Ammonium transporter 1 member 2	2 × 10^-25^
GS02431	8	2.0 × 10^-3^	RABL5_DANRE	Rab-like protein 5	3 × 10^-28^
GS01141	6	7.8 × 10^-3^	TM9S2_RAT	Transmembrane 9 superfamily member 2	7 × 10^-84^
GS00197	6	7.8 × 10^-3^	ARF1_SALBA	ADP-ribosylation factor 1	1 × 10^-70^
Nucleotide transport and metabolism					
GS00406	7	3.9 × 10^-3^	NDK7_HUMAN	Nucleoside diphosphate kinase 7	2 × 10^-32^
Posttranslational modification, protein turnover, chaperones					
GS00465	6	7.8 × 10^-3^	TRAP1_DICDI	TNF receptor-associated protein 1 homolog, mitochondrial precursor	1 × 10^-98^
GS04078	6	7.8 × 10^-3^	BIRC7_HUMAN	Baculoviral IAP repeat-containing protein 7	2 × 10^-06^
GS01693	6	7.8 × 10^-3^	IQCAL_HUMAN	IQ and AAA domain-containing protein ENSP00000340148	3 × 10^-41^
GS00324	8	2.0 × 10^-3^	TTLL4_HUMAN	Tubulin polyglutamylase	1 × 10^-42^
GS06285	7	3.9 × 10^-3^	IAP3_NPVOP	Apoptosis inhibitor 3	1 × 10^-05^
GS03771	6	7.8 × 10^-3^	14335_ORYSJ	14-3-3-like protein GF14-E	1 × 10^-34^
GS01424	6	7.8 × 10^-3^	PCSK7_RAT	Proprotein convertase subtilisin/kexin type 7 precursor	2 × 10^-08^
GS01530	6	7.8 × 10^-3^	YDM9_SCHPO	Uncharacterized RING finger protein C57A7.09 precursor	3 × 10^-07^
GS00537	7	3.9 × 10^-3^	XRP2_XENLA	Protein XRP2	5 × 10^-20^
Signal transduction mechanisms					
GS01456	8	2.0 × 10^-3^	CML12_ARATH	Calmodulin-like protein 12	3 × 10^-11^
GS03471	6	7.8 × 10^-3^	DNAL1_CHLRE	Flagellar outer arm dynein light chain 1	1 × 10^-52^
**GS00910**	14	3.1 × 10^-5^	KAPR2_DROME	amp-dependent protein kinase type II regulatory subunit	2 × 10^-08^
GS04612	6	7.8 × 10^-3^	RHOM_DROME	Protein rhomboid	3 × 10^-08^
GS02444	11	2.4 × 10^-4^	ANR11_HUMAN	Ankyrin repeat domain-containing protein 11	3 × 10^-09^
GS02191	6	7.8 × 10^-3^	LRC50_HUMAN	Leucine-rich repeat-containing protein 50	1 × 10^-54^
**GS00234**	7	3.9 × 10^-3^	KCC1A_RAT	Calcium/calmodulin-dependent protein kinase type 1	1 × 10^-51^
**GS00184**	6	7.8 × 10^-3^	TNI3K_RAT	Serine/threonine-protein kinase	3 × 10^-14^
GS01544	7	3.9 × 10^-3^			
GS03554	7	3.9 × 10^-3^	^†^PH	Plecstrin homology domain	3 × 10^-09^
Transcription					
GS00117	8	2.0 × 10^-3^	MYB_DROME	Myb protein	9 × 10^-06^
**GS00273**	8	2.0 × 10^-3^	MYB_CHICK	Myb proto-oncogene protein (C-myb)	3 × 10^-34^
GS01762	6	7.8 × 10^-3^	MYBB_CHICK	Myb-related protein B	5 × 10^-06^

Only clusters with zero ESTs originating from the 2N library are shown. The number of 1N EST reads in each cluster and the *P*-value for significance of the difference between libraries are shown. When no Swiss-Prot homolog was detected, ID and homology values for the top Uniprot homolog are given (indicated by an asterisk), or the CDD name and homology values are given (indicated by †). Clusters are arranged by KOG class. Clusters in bold were chosen for RT-PCR validation. Additional data file 4 gives a complete list of all clusters predicted to be 1N-specific by statistical comparison of libraries.

**Table 6:** EST clusters without KOG assignment predicted to be highly 1N-specific based on statistical comparison of libraries

Cluster ID	Number of 1N ESTs	*P*-value	Homolog ID	Homolog description	BLAST
**GS00667**	7	3.9 × 10^-3^	DYHC_ANTCR	Dynein beta chain, ciliary	2 × 10^-52^
GS01639	6	7.8 × 10^-3^	BSN1_BACAM	Extracellular ribonuclease precursor	2 × 10^-10^
GS02259	7	3.9 × 10^-3^	GAS8_CHLRE	Growth arrest-specific protein 8 homolog (Protein PF2)	2 × 10^-82^
GS00095	6	7.8 × 10^-3^	DYHB_CHLRE	Dynein beta chain, flagellar outer arm	1 × 10^-35^
GS03902	6	7.8 × 10^-3^	*Q94EY1_CHLRE	Predicted protein	8 × 10^-14^
GS00471	6	7.8 × 10^-3^	*A9BCA5_PROM4	Putative uncharacterized protein	2 × 10^-80^
GS00126	7	3.9 × 10^-3^	STCE_ECO57	Metalloprotease stcE precursor	5 × 10^-31^
**GS00242**	8	2.0 × 10^-3^	SPT17_HUMAN	Spermatogenesis-associated protein 17	8 × 10^-11^
**GS00012**	9	9.8 × 10^-4^	DYH6_HUMAN	Axonemal beta dynein heavy chain 6	1 × 10^-129^
GS00276	11	2.4 × 10^-4^	PLMN_MACEU	Plasminogen precursor	2 × 10^-15^
GS00140	10	4.9 × 10^-4^	Y326_METJA	Uncharacterized protein MJ0326	1 × 10^-64^
GS01207	8	2.0 × 10^-3^	CF206_MOUSE	Uncharacterized protein C6orf206 homolog	2 × 10^-26^
GS01392	9	9.8 × 10^-4^	DYH3_MOUSE	Axonemal beta dynein heavy chain 3	5 × 10^-89^
GS02146	9	9.8 × 10^-4^	CCD37_MOUSE	Coiled-coil domain-containing protein 37	3 × 10^-21^
GS00154	6	7.8 × 10^-3^	IQCG_MOUSE	IQ domain-containing protein G	3 × 10^-22^
GS02689	6	7.8 × 10^-3^	RNF32_MOUSE	RING finger protein 32	2 × 10^-11^
GS00461	10	4.9 × 10^-4^	NAT_MYCSM	Arylamine N-acetyltransferase	2 × 10^-21^
GS03363	6	7.8E-03	*A1UWW2_BURMS	RemN protein	6 × 10^-06^
GS00524	8	2.0E-03	*Q0 MYX1_EMIHU	Putative uncharacterized protein	3 × 10^-55^
GS00907	7	3.9E-03	*Q0 MYV7_EMIHU	Putative uncharacterized protein	7 × 10^-07^
**GS02894**	6	7.8E-03	*Q9ZTY0_EMIHU	Putative calcium binding protein	2 × 10^-07^
GS02739	8	2.0E-03	*Q2 MCN4_HYDAT	HyTSR1 protein	5 × 10^-07^
GS01630	7	3.9E-03	*A0L4Q4_MAGSM	Cadherin	4 × 10^-12^
GS02194	8	2.0E-03	*C1 MZQ6_9CHLO	Predicted protein	9 × 10^-07^
GS00043	7	3.9E-03	*C1NAB5_9CHLO	Predicted protein	6 × 10^-14^
GS02204	6	7.8E-03	*C1EGP6_9CHLO	Predicted protein	2 × 10^-09^
GS02009	6	7.8E-03	*A9UNX1_MONBE	Predicted protein	4 × 10^-24^
GS03800	8	2.0E-03	*Q0JCM6_ORYSJ	Os04 g0461600 protein	3 × 10^-09^
GS00472	6	7.8E-03	*Q00Y28_OSTTA	Chromosome 12 contig 1, DNA sequence	2 × 10^-13^
GS00972	6	7.8E-03	*A0DFH5_PARTE	Chromosome undetermined scaffold_49, whole genome shotgun sequence	7 × 10^-13^
GS00363	8	2.0E-03	*A9RPM7_PHYPA	Predicted protein	2 × 10^-07^
**GS00157**	12	1.2E-04	*Q0E9S1_PLEHA	Putative beta-type carbonic anhydrase	9 × 10^-70^
GS00753	6	7.8E-03	*Q0E9R5_PLEHA	Putative uncharacterized protein	2 × 10^-30^
**GS02990**	15	1.5E-05	*Q2NSA6_SODGM	Hypothetical phage protein	5 × 10^-06^
GS00195	7	3.9E-03	*C4EA11_STRRS	Putative uncharacterized protein	4 × 10^-12^
GS01216	8	2.0E-03	*B4WU30_9SYNE	Putative uncharacterized protein	1 × 10^-06^
GS00006	8	2.0E-03	*B8BYB9_THAPS	Predicted protein	4 × 10^-12^
GS00629	6	7.8E-03	*B8LBM2_THAPS	Predicted protein	1 × 10^-32^
GS03100	8	2.0E-03	*A5AXV4_VITVI	Putative uncharacterized protein	7 × 10^-07^
					
Orphan genes tested					
**GS01257**	25	1.5 × 10^-8^			
**GS01805**	16	7.7 × 10^-6^			

Only clusters with zero ESTs originating from the 2N library are shown, and only the orphans confirmed by RT-PCR are included in this table. Homolog IDs are marked as in Table 5. Additional data file 4 gives a complete list of all clusters predicted to be 1N-specific by statistical comparison of libraries.

**Table 7:** KOG-assigned EST clusters predicted to be highly 2N-specific based on statistical comparison of libraries

Cluster ID	Number of 2N ESTs	*P*-value	Homolog ID	Homolog description	BLAST
Carbohydrate transport and metabolism					
**GS00451**	7	3.9 × 10^-3^	PIP25_ARATH	Probable aquaporin PIP2-5	1 × 10^-34^
GS00433	8	1.9 × 10^-3^	F26_RANCA	6PF-2-K/Fru-2,6-P2ASE liver/muscle isozymes	3 × 10^-40^
Cell wall/membrane/envelope biogenesis					
GS01290	8	1.9 × 10^-3^	ASB3_BOVIN	Ankyrin repeat and SOCS box protein 3 (ASB-3)	9 × 10^-06^
Chromatin structure and dynamics					
**GS02435**	6	7.8 × 10^-3^	H4_OLILU	Histone H4	8 × 10^-33^
Cytoskeleton					
GS00171	6	7.8 × 10^-3^	EXS_ARATH	Leucine-rich repeat receptor protein kinase EXS precursor	1 × 10^-08^
Energy production and conversion					
GS00763	6	7.8 × 10^-3^	QORH_ARATH	Putative chloroplastic quinone-oxidoreductase homolog	6 × 10^-25^
GS01632	7	3.9 × 10^-3^	CYPD_BACSU	Probable bifunctional P-450/NADPH-P450 reductase 1	2 × 10^-43^
General function prediction only					
GS00580	9	9.7 × 10^-4^	YMO3_ERWST	Uncharacterized protein in mobD 3' region	6 × 10^-07^
GS02524	7	3.9 × 10^-3^	^†^RKIP	Raf kinase inhibitor protein (RKIP), Phosphatidylethanolamine-binding protein (PEBP)	1 × 10^-06^
Inorganic ion transport and metabolism					
**GS00463**	8	1.9 × 10^-3^	NCKXH_DROME	Probable Na^+^/K^+^/Ca^2+ ^exchanger CG1090	1 × 10^-22^
**GS05051**	7	3.9 × 10^-3^	B3A2_RAT	Anion exchange protein 2 (AE2 anion exchanger)	8 × 10^-14^
Intracellular trafficking, secretion, and vesicular transport					
**GS02941**	9	9.7 × 10^-4^	STX1A_CAEEL	Syntaxin-1A homolog	2 × 10^-19^
Lipid transport and metabolism					
GS00955	7	3.9 × 10^-3^	S5A1_MACFA	3-oxo-5-alpha-steroid 4-dehydrogenase 1	3 × 10^-54^
Posttranslational modification, protein turnover, chaperones					
GS06447	6	7.8 × 10^-3^	CLPP3_ANASP	Probable ATP-dependent Clp protease proteolytic subunit 3	2 × 10^-31^
GS02029	8	1.9 × 10^-3^	UBCY_ARATH	Ubiquitin-conjugating enzyme E2-18 kDa	4 × 10^-20^
GS03925	8	1.9 × 10^-3^	FKBP4_DICDI	FK506-binding protein 4 (peptidyl-prolyl cis-trans isomerase)	1 × 10^-07^
Replication, recombination and repair					
GS00109	8	1.9 × 10^-3^	MCM2_XENTR	DNA replication licensing factor mcm2	1 × 10^-109^
Secondary metabolites biosynthesis, transport and catabolism					
GS00417	6	7.8 × 10^-3^	WBC11_ARATH	White-brown complex homolog protein 11	9 × 10^-28^
Signal transduction mechanisms					
GS00826	6	7.8 × 10^-3^	STK4_BOVIN	Serine/threonine-protein kinase 4	2 × 10^-47^
GS00712	7	3.9 × 10^-3^	PI4K_DICDI	Phosphatidylinositol 4-kinase	3 × 10^-43^
GS00083	7	3.9 × 10^-3^	SHKE_DICDI	Dual specificity protein kinase shkE	9 × 10^-22^
GS01230	7	3.9 × 10^-3^	^†^PP2Cc	Serine/threonine phosphatases, family 2C, catalytic domain	2 × 10^-08^

Only clusters with zero ESTs originating from the 1N library are shown. The number of 2N EST reads in each cluster and the *P*-value for significance of the difference between libraries are shown. Homolog IDs are marked as in Table 5. Clusters are arranged by KOG class. Clusters in bold were chosen for RT-PCR validation. Additional data file 5 gives a complete list of all clusters predicted to be 2N-specific by statistical comparison of libraries.

**Table 8:** EST clusters without KOG assignment predicted to be highly 2N-specific based on statistical comparison of libraries

Cluster ID	Number of 2N ESTs	*P*-value	Homolog ID	Homolog description	BLAST
GS00092	6	7 × 10^-17^	*B1X317_CYAA5	Putative uncharacterized protein	7 × 10^-17^
**GS03351**	14	2 × 10^-06^	*Q0 MYU5_EMIHU	Putative arachidonate 15-lipoxygenase second type	2 × 10^-06^
GS05210	7	1 × 10^-25^	*C1AEM4_GEMAT	Putative glutamine cyclotransferase	1 × 10^-25^
GS01732	8	1 × 10^-19^	*A7WPV6_KARMI	Putative uncharacterized protein	1 × 10^-19^
GS02223	7	1 × 10^-31^	*C1 MGG4_9CHLO	Predicted protein	1 × 10^-31^
GS05779	6	2 × 10^-17^	*C1E2K5_9CHLO	Predicted protein	2 × 10^-17^
GS06362	6	2 × 10^-15^	*A9V2G5_MONBE	Predicted protein	2 × 10^-15^
GS00766	7	3 × 10^-07^	*B7G9 M0_PHATR	Predicted protein	3 × 10^-07^
GS03302	7	1 × 10^-32^	*B7G0S2_PHATR	Predicted protein	1 × 10^-32^
GS03476	9	2 × 10^-09^	*B7FQM3_PHATR	Predicted protein	2 × 10^-09^
GS00513	8	6 × 10^-08^	*Q7V952_PROMM	Putative uncharacterized protein	6 × 10^-08^
GS01720	9	8 × 10^-06^	*B2ZYD9_9CAUD	Nucleoside-diphosphate-sugar pyrophosphorylase-like protein	8 × 10^-06^
GS05985	7	6 × 10^-06^	*B0J8I4_RHILT	Putative uncharacterized protein	6 × 10^-06^
GS01421	6	7 × 10^-11^	*B9S8J5_RICCO	Putative uncharacterized protein	7 × 10^-11^
GS05596	8	2 × 10^-11^	*B8 MI73_TALSN	Putative uncharacterized protein	2 × 10^-11^
GS00659	6	5 × 10^-22^	*A4VDD7_TETTH	Putative uncharacterized protein	5 × 10^-22^
***GS11002***	16	7.6 × 10^-6^			
**GS02507**	12	1.2 × 10^-4^			
**GS01164**	10	4.9 × 10^-4^			
**GS01802**	10	4.9 × 10^-4^			

Only clusters with zero ESTs originating from the 1N library are shown, and only the orphans confirmed by RT-PCR are included in this table. Homolog IDs are marked as in Table 5. Clusters in bold were chosen for RT-PCR validation (cluster GS11002 is shown in bold italics, the only cluster tested in which abundant RT-PCR product could also be detected from 1N cells). Additional data file 5 gives a complete list of all clusters predicted to be 2N-specific by statistical comparison of libraries.

The most significantly differentially represented highly 1N-specific clusters (*P *= 10^-9^~10^-4^) included a homolog of histone H4 (cluster GS09138; 1N ESTs = 13 versus 2N ESTs = 0), a homolog of cAMP-dependent protein kinase type II regulatory subunit (GS00910; 1N = 14 versus 2N = 0), a transcript encoding a DNA-6-adenine-methyltransferase (Dam) domain (GS02990) and four other clusters of unknown functions. Other predicted highly 1N-specific clusters included several flagellar components, and three clusters showing homology to the Myb transcription factor superfamily (GS00117, GS00273, GS01762; 1N = 8, 8, and 6 ESTs, respectively, and 2N = 0 in all cases). The most significantly differentially represented highly 2N-specific clusters (*P *= 10^-7^~10^-4^) included a cluster of unknown function (GS11002; 1N = 0 and 2N = 16) and a weak homolog of a putative *E. huxleyi *arachidonate 15-lipoxygenase (E-value 2 × 10^-6^). Of the 199 highly 1N-specific clusters, 40 had homologs in the KOG database, including 9 clusters (22.5%) assigned to the 'posttranslational modification, protein turnover, chaperones' class and 10 (25.0%) assigned to the 'signal transduction mechanisms' class. The KOG classes for the 22 2N-specific clusters with KOG matches appeared more evenly distributed, with slightly more abundance in the 'signal transduction mechanisms' class (4 clusters, 18.2%). As discussed in the 'Validation and exploration of the predicted differential expression of selected genes' section of the Results, RT-PCR tests validated these predictions of differential expression with a high rate of success.

#### Taxonomic distribution of transcript homology varies over the life cycle

**Figure 5:**
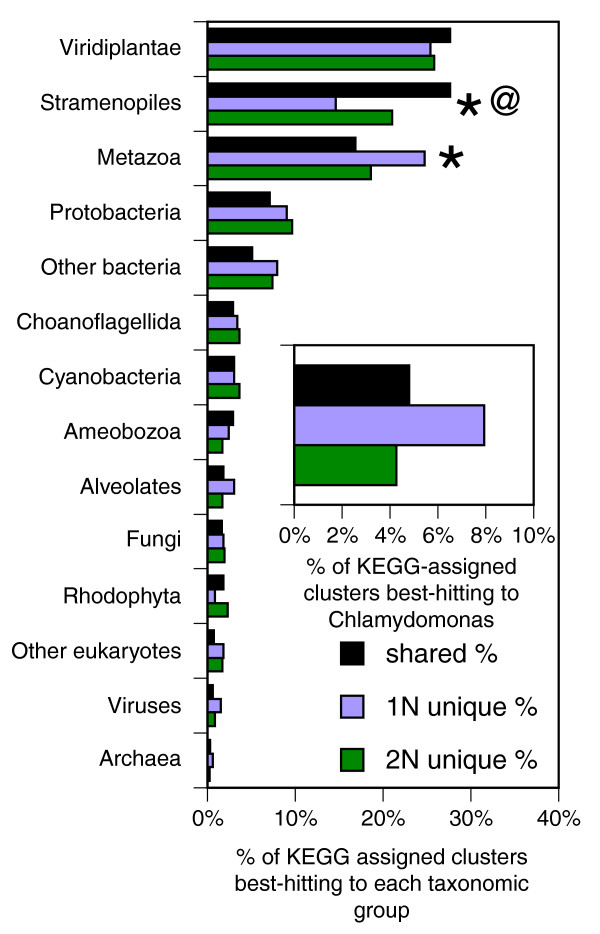
The taxonomic distribution of homology. Shown are the percentages of clusters with KEGG homologs that have the 'best hit' in each taxonomic group. Indicated are cases where the proportion of clusters best hitting to the taxonomic group differs between 1N-unique and 2N-unique (asterisks) or between 1N-unique and shared clusters (at symbol (@)), tested as above. The inset shows the proportion of all assigned clusters that are accounted for by best-hits to *Chlamydomonas reinhardtii *(a subset of those which are best-hits to Viridiplantae). The differences between 1N-unique and 2N-unique, and between 1N-unique and shared clusters were significant (*P *< 0.002).

To characterize the taxonomic distribution of the homologs of EST clusters, we performed BLASTX searches against a combined database, which includes the proteomes from 42 selected eukaryotic genomes taken from the Kyoto Encyclopedia of Genes and Genomes (KEGG) database (see Additional data file 6 for a list of selected genomes from the KEGG database) as well as prokaryotic/viral sequences from the UniProt database. There were 4,055 clusters (31.1%; 1,731 shared, 1,083 1N-unique and 1,241 2N-unique clusters) with significant homology in the database (E-value <1 × 10^-10^), with Viridiplantae, stramenopiles, and metazoans receiving the largest numbers of hits (72.1%, 66.4%, and 60.9%, respectively, of all clusters with KEGG hits). These clusters were classified by the taxonomic group of their closest BLAST homolog (that is, 'best hit'). The distribution of the taxonomic group was found to substantially vary among the shared, 1N-unique and 2N-unique clusters. Shared clusters had a significantly higher proportion of best hits to stramenopiles compared to both 1N-unique and 2N-unique clusters, while 1N-unique clusters had a significantly lower percentage of best hits to stramenopiles than 2N-unique clusters. In contrast, metazoans received a significantly greater portion of best hits from 1N-unique than from 2N-unique and shared clusters. Consistent with the above functional analysis, the KOG class 'signal transduction mechanisms' was over-represented in clusters best-hitting to metazoans (11.0%) compared to all clusters with homologs in KEGG (5.0%) or clusters best-hitting to Viridiplantae (4.8%) (*P *= 2.9 × 10^-13 ^and 5.0 × 10^-6^, respectively; Fishers exact test). There was no difference among 1N-unique, 2N-unique, and shared clusters in the proportion of clusters with best hits to Viridiplantae (Figure 5). However, among the Viridiplantae best hits, a significantly greater proportion of 1N-unique clusters was found to be best-hitting to *Chlamydonomas reinhardtii *(Figure 5), the only free-living motile, haploid genome from Viridiplantae represented in our database.

Of all clusters best-hitting to either Viridiplantae, stramenopiles, or metazoans, the shared clusters had the highest percentage of clusters (53.6%) with homologs in all three groups, and the lowest percentage of clusters (3.1%) with homologs only in metazoans (Figure S5 in Additional data file 1). Clusters with homologs in stramenopiles were significantly over-represented among shared clusters and under-represented in 1N-unique clusters relative to 2N-unique clusters.

**Figure 6:**
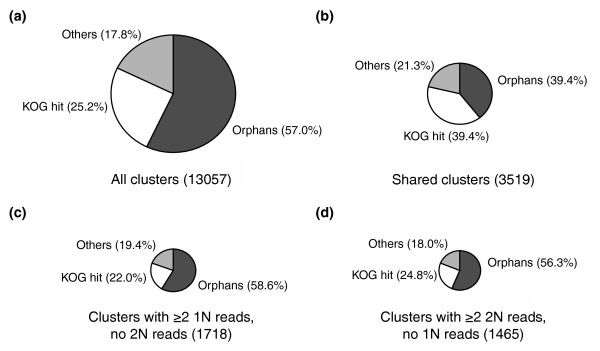
The proportion of orphan clusters. Non-orphan clusters that do not have hits in the KOG database are also represented (Others). **(a) **All clusters. **(b) **Shared clusters composed of reads in both 1N and 2N libraries. **(c) **Potentially 1N-specific clusters composed of two or more reads in the 1N library but zero in the 2N library. **(d) **Potentially 2N-specific clusters composed of two or more reads in the 2N library but zero in the 1N library.

The vast majority (7,442 clusters; 57.0%) of the total EST clusters were orphans (Figure 6a). One of the main causes of the high orphan proportion might be the presence of many short EST clusters with only one or a few ESTs. The non-orphan clusters (having matches in UniProt, KOG, or the conserved domains database (CDD)) exhibited a significantly higher average number of reads per cluster (3.67, combining reads from both libraries) than orphan clusters (2.39; *P *< 0.0001, Mann-Whitney test). In a similar way, the orphan proportion decreased to 39.4% for the shared core clusters (Figure 6b), which have an average of 6.25 ESTs per cluster. However, a more detailed analysis indicated that the size of clusters (that is, the number of ESTs in the cluster) may not be the sole reason for the abundance of the orphan clusters. For instance, 58.6% of 1N-unique clusters with two or more ESTs were orphan clusters (Figure 6c). Furthermore, an even higher orphan proportion (63.9%) was obtained when these 1N-unique clusters were limited to the 119 clusters represented by ≥ 7 ESTs. Similarly high orphan proportions were also obtained for the 2N-unique clusters (56.3% for the clusters with ≥ 2 ESTs (Figure 6d), and 55.0% for the 60 clusters with ≥ 7 ESTs.). Overall, these results suggest that our transcriptomic data include many new genes probably unique to haptophytes, coccolithophores or *E. huxleyi*, and that many of these unique genes may be preferentially expressed in one of the two life cycle phases.

### Validation and exploration of the predicted differential expression of selected genes

We examined how well our *in silico *comparison of the two normalized libraries successfully identified gene content differentiating the two transcriptomes based on in-depth sequence/bibliographic analysis and RT-PCR assays (summarized in Tables S1 and S2 in Additional data file 7). We began with homologs of eukaryotic flagellar-associated proteins. This large group of proteins is well-conserved across motile eukaryotes. Genes for proteins known to be exclusively present in flagellar or basal bodies are expected to be specifically expressed in the motile 1N stage of *E. huxleyi*, whereas those for proteins known to also serve functions in the cell body may also be expressed in non-motile cells. Thus, flagella-related genes serve as a particularly useful initial validation step. Next, we examined several other clusters with strong *in silico *signals for differential expression between the 1N and 2N libraries. Finally, we explored clusters homologous to known Ca^2+ ^and H^+ ^transporters, potentially involved in the calcification process of 2N cells, and histones, which might play roles in epigenetic control of 1N versus 2N differentiation. In total, we tested the predicted expression patterns of 39 clusters representing 38 different genes. The predicted expression pattern (1N-specific, 2N-specific, or shared) was confirmed for 37 clusters (36 genes), demonstrating a high rate of success of the *in silico *comparison of transcriptome content.

#### Motility-related clusters

A total of 156 *E. huxleyi *EST clusters were found to be homologous to 85 flagellar-related or basal body-related proteins from animals or *C. reinhardtii*, a unicellular green alga serving as a model organism for studies of eukaryotic flagella/cilia [26-28] (Tables 9 and 10). This analysis combined a systematic BLAST searche using 100 *C. reinhardtii *motility-related proteins identified by classic biochemical analysis [27] with additional homology searches (detailed analysis provided in Additional data files 8 and 9). Of the 100 *C. reinhardtii *proteins, 64 were found to have one or more similar sequences in the *E. huxleyi *EST dataset. We could also identify homologs for six of the nine Bardet-Biedl syndrome (BBS) proteins known to be basal body components [29,30]. Excluding 64 clusters closely related to proteins known to play additional roles outside the flagellum/basal body (such as actin and calmodulin) and 10 clusters showing a relatively low level of sequence similarity to flagellar-related proteins, 82 of the 156 clusters were considered highly specific to motility. Remarkably, these clusters were found to be represented by 252 ESTs from the 1N but 0 ESTs from the 2N library (Table 9). In contrast, clusters related to proteins with known possible roles outside of flagella tended to be composed of ESTs from both 1N and 2N libraries, as expected (Table 10).

**Table 9:** Distribution of EST reads and clusters related to proteins highly specific to cilia/flagella or basal bodies

	Number of 1N clusters	Number of 2N clusters	Number of 1N ESTs	Number of 2N ESTs
**Outer dynein arm**				
Dynein heavy chain alpha (ODA11)	2	0	8	0
Dynein heavy chain beta (ODA4)	3	0	12	0
Outer dynein arm intermediate chain 1 (ODA9)	1	0	2	0
Dynein, 70 kDa intermediate chain, flagellar outer arm (ODA6)	2	0	7	0
Outer dynein arm light chain 1 (DLC1)	1	0	6	0
Outer dynein arm light chain 2 (ODA12)	1	0	5	0
Outer dynein arm light chain 5, 14KD (DLC5)	3	0	9	0
Outer dynein arm light chain 7b (DLC7b)	1	0	2	0
Outer dynein arm light chain 8, 8KD (FLA14)	2	0	3	0
Outer dynein arm docking complex 2 (ODA-DC2)	1	0	5	0
Outer dynein arm docking complex 3 (ODA-DC3)	2	0	7	0
				
**Inner dynein arm**				
Inner dynein arm heavy chain 1-alpha (DHC1a)	1	0	1	0
Inner dynein arm heavy chain 1-beta (DHC1b/IDA2)	3	0	6	0
Dynein heavy chain 2 (DHC2)	3	0	15	0
Dynein heavy chain 8 (DHC8)	1	0	1	0
Dynein heavy chain 9 (DHC9)	3/2	1/0	15/14	4/0
Inner dynein arm I1 intermediate chain IC14 (IDA7)	1	0	4	0
Inner dynein arm I1 intermediate chain (IC138)	1	0	3	0
Inner dynein arm ligh chain p28 (IDA4)	1	0	2	0
Dynein light chain tctex1 (TCTEX1)	2	0	5	0
Dynein light chain Tctex2b	1	0	4	0
				
**Radial spoke associated proteins**				
Radial spoke protein 1	1	0	1	0
Radial spoke protein 2 (PF24)	1/0	0	3/0	0/0
Radial spoke protein 4 (PF1)	1	0	3	0
Radial spoke protein 9	1	0	8	0
Radial spoke protein 10	2/0	0/0	6/0	0/0
Radial spoke protein 11	1	0	4	0
Radial spoke protein 14	1	0	1	0
Radial spoke protein 16	1/0	0/0	1/0	0'0
Radial spoke protein 23	1/0	0/0	8/0	5/0
				
**Central pair**				
Central pair protein (PF16)	2	0	5	0
Central pair associated WD-repeat protein	1	0	4	0
Central pair protein (PF6)	1	0	1	0
				
**Intraflagellar transport**				
Dynein 1b light intermediate chain (D1bLIC)	1	0	1	0
Intraflagellar transport protein 20 (IFT2)	1	0	2	0
Intraflagellar transport protein 57 (IFT57), alternative version	1	0	4	0
Intraflagellar transport protein 72 and 74 (IFT72/74)	1	0	1	0
Intraflagellar transport protein 80 (CHE2)	2	0	6	0
Intraflagellar transport protein 81 (IFT81)	1	0	4	0
Intraflagellar transport protein 121 (IFT121)	1	0	2	0
Intraflagellar transport protein 139 (IFT139)	1	0	1	0
Intraflagellar transport protein 140 (IFT140)	2/1	0/0	2/1	0/0
Intraflagellar transport protein 172 (IFT172)	1	0	1	0
				
**Miscellaneous**				
Dynein regulatory complex protein (PF2)	1	0	7	0
Tektin	1	0	3	0
Conserved uncharacterized flagellar associated protein FAP189	2	0	9	0
Conserved uncharacterized flagellar associated protein FAP58	1	0	3	0
Flagellar protofilament ribbon protein (RIB43a)	1	0	6	0
Nucleoside-diphosphokinase regulatory subunit p72 (RIB72)	1	0	1	0
				
**Proteins found by manual search of Uniprot/Swiss-Prot hits related to eukaryotic flagella and basal body**				
Subunit of axonemal inner dynein arn (A9ZPM1_CHLRE)	1	0	1	0
Flagellar associated protein (A8J1V4_CHLRE)	1	0	4	0
Flagellar associated protein (A8JDM7_CHLRE)	1	0	1	0
Flagellar associated protein (A8J0N6_CHLRE)	1	0	4	0
Flagellar associated protein (A8J7D6_CHLRE)	1	0	4	0
Flagellar associated protein (A8JB22_CHLRE)	1	0	4	0
Flagellar associated protein (A8HZK8_CHLRE)	2	0	6	0
Flagellar associated protein (A8I9E8_CHLRE)	2	0	5	0
Flagellar associated protein (A7S8J6_NEMVE)	1	0	6	0
Flagellar associated protein (A8HMZ4_CHLRE)	1	0	1	0
Chlamydomonas minus and plus agglutinin (AAS07042.1)	1/0	0/0	3/0	0/0
Flagellar/basal body protein (A8J795_CHLRE)	1	0	2	0
Flagellar/basal body protein (A8I6L8_CHLRE)	1	0	1	0
Bardet-Biedl syndrome 1 protein (A8JEA1_CHLRE)	1	0	4	0
Dynein heavy chain beta (ODA4)	1	0	1	0
ADP-ribosylation factor-like protein 6 (BBS3) (Q9HF7_HUMAN)	1	0	3	0
Bardet-Biedl syndrome 5 protein (BBS5_DANRE)	1	0	2	0
Bardet-Biedl syndrome 7 protein (BBS7_MOUSE)	1	0	1	0
Bardet-Biedl syndrome 9 protein (PTHB1_HUMAN)	1	0	2	0
				
**Totals**	90/82	1/0	275/252	9/0

The numbers of homologous clusters containing ESTs originating from 1N and 2N libraries are shown. Also shown are numbers of component ESTs from 1N and 2N libraries. Potential 'false positive' homologs were identified (for example, clusters with stronger homology to non-flagellar proteins). In such cases, the numerator represents the number of clusters or ESTs including potential false positive clusters, and the denominator when false positive clusters are excluded. Detailed analysis of all motility-related clusters is provided in Additional data files 7 and 8.

**Table 10:** Distribution of EST reads and clusters related to cilia/flagella components that also have non-ciliary functions

	Number of 1N clusters	Number of 2N clusters	Number of 1N ESTs	Number of 2N ESTs
**Tubulins**				
Alpha-1 tubulin (TUA1, TUA2)	6	2	10	5
Beta-1 tubulin (TUB1, TUB2)	3	1	4	2
				
**Inner dynein arm**				
Actin, inner dynein arm intermediate chain (IDA5)	7	6	11	13
Caltractin/centrin 20 kDa calcium-binding protein (VFL2)	8	4	13	22
				
**Central pair**				
Kinesin-like protein 1 (KLP1)	1	0	1	0
Phophatase 1 (PP1a)	3	2	4	8
				
**Intraflagellar transport**				
Kinesin-II associated protein (KAP1)	1	0	1	0
Cytoplasmic dynein heavy chain 1b (DHC1b)	2	0	7	0
				
**Miscellaneous**				
Microtubule-associated protein (EB1)	1	1	6	4
Glycogen synthase kinase 3 (GSK3)	2	2	5	12
Calmodulin (CAM)	7	3	19	10
Deflagellation inducible protein, 13KD (DIP13)	0	1	0	1
Heat shock 70 kDa protein (HSP70A)	4	4	11	8
Phototropin, blue light receptor (PHOT)	5/4	2/2	17/15	6/6
Protein phosphatase 2a (PP2A-r2)	0	3	0	3
				
**Proteins found by manual search of Uniprot/Swiss-Prot hits related to eukaryotic flagella and basal body**				
Flagellar associated protein (A8JAF7_CHLRE)	1	1	6	4
Flagellar associated protein (A8JC09_CHLRE)	1	1	6	1
				
**Totals**	52/51	33/33	121/119	99/99

Table is organized as Table 9. Additional data files 7 and 8 contain a detailed analysis.

The abundance of 1N-unique EST clusters with the closest homolog in Metazoa (Figure 5) appears to be partially due to the expression of genes related to flagellar components in 1N cells. In fact, 58 (37.2%) of the 156 motility-related clusters had best-hits to Metazoa in the KEGG database, compared to only 789 (14.1%) of all 5,614 non-orphan clusters (*P *= 2.9 × 10^-13^).

**Figure 7:**
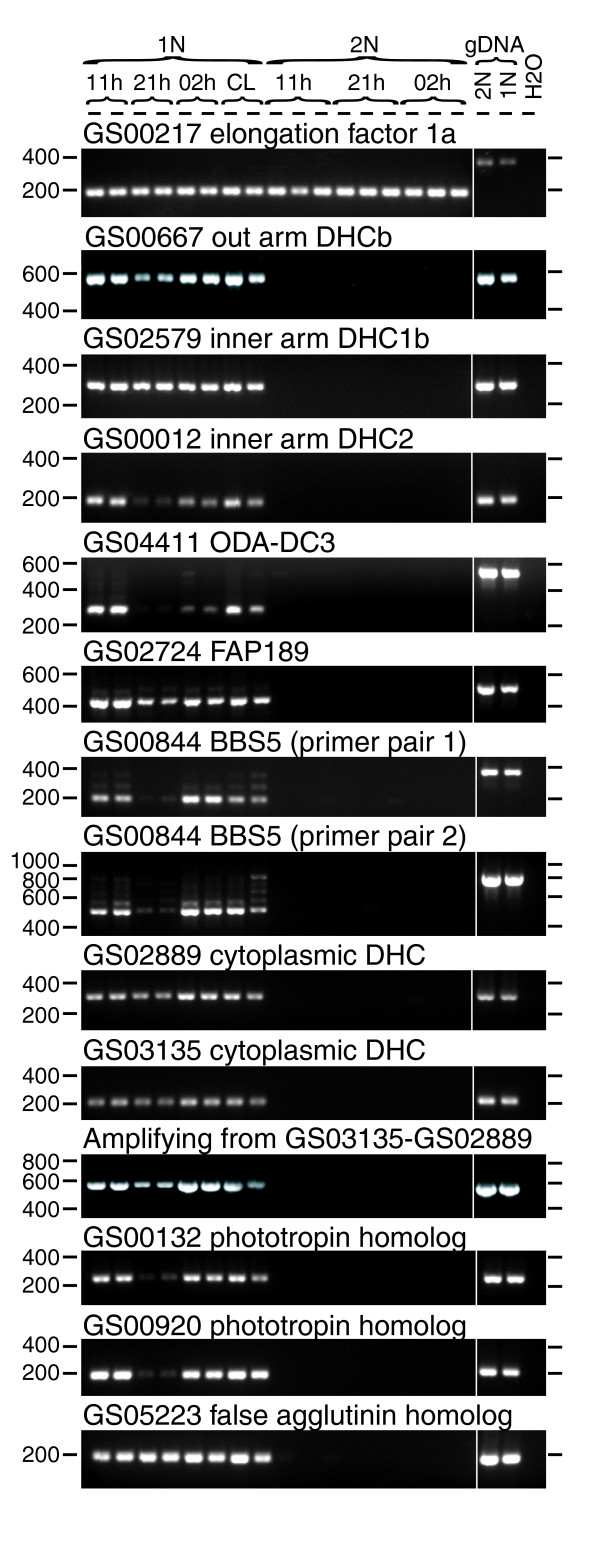
RT-PCR confirmation of expression of selected flagellar-related genes only in 1N cells. All reactions were run with the same RT+ cDNA samples. The RT-PCR shown at the top used the elongation factor 1α (GS000217) as a positive (loading) control showing successful cDNA amplification occurred in all samples. RT- control reactions prepared from the same RNA were run for nine of the PCRs shown here and no contaminating genomic DNA (gDNA) was ever found (see examples with RT- reactions included in Figure S6 in Additional data file 1). For clarity, RT- control reactions run simultaneously have been cut out here. Positions of molecular weight markers on each side of the gel are shown. The sample identifiers are listed for each lane at the top of the gel. 11 h, harvested at 11 h (late morning); 21 h, harvested at 21 h (early evening, time of S-phase); 02 h, harvested at 02 h (after cell division); CL, cultures (1N only) exposed to continuous light.

Six core structural components of the flagellar apparatus were chosen for RT-PCR tests (Figure 7). These included three flagellar dynein heavy chain (DHC) paralogs (GS00667, GS02579 and GS00012), a homolog of the outer dynein arm docking complex protein ODA-DC3 (GS04411), a homolog of FAP189 and FAP58/MBO2, highly conserved but poorly characterized coiled-coil proteins identified in the *C. reinhardti *flagellar proteome [27] (GS02724), and a homolog of the highly conserved basal body protein BBS5 (GS00844) [31]. All showed expression restricted to 1N cells; no signal could be detected for these five clusters in any 2N RNA samples. Curiously, three non-overlapping primer sets designed to GS000844 (BBS5) all detected evidence of incompletely spliced transcript products, suggesting its regulation by alternative splicing.

GS05223, containing three ESTs from the 1N library and none from the 2N, showed a significant sequence similarity to *C. reinhardtii *minus and plus agglutinins (BLASTX, E-values 3 × 10^-5 ^and 8 × 10^-6^, respectively), flagellar associated proteins involved in sexual adhesion [32]. RT-PCR confirmed that expression of GS05223 was highly specific to 1N cells, being undetectable in 2N cells (Figure 7). However, inspection of the BLASTX alignment between GS05223 and *C. reinhardtii *agglutinins revealed that the sequence similarity was associated with the translation of the reverse-complement of GS05223. We also found that all of the three ESTs in GS05223 contained poly-A tails, so must be expressed in the forward direction. Therefore, we concluded that GS05223 represents an unknown haploid-specific gene product that may not be related to flagellar functions.

Next we investigated four clusters that are homologous to proteins known to often have additional, non-flagellar roles in the cytoplasm, but that were represented only in the 1N library. Two clusters (GS02889 and GS03135) displayed homology to cytoplasmic dynein heavy chain (DHC), which is associated with flagella/cilia due to its role in intraflagellar transport. In animals and amoebozoa, it also has non-flagellar functions such as intracellular transport and cell division [33]; however, both clusters showed potential 1N-specific expression, being represented by two and five 1N ESTs and zero 2N ESTs, respectively, and RT-PCR confirmed the predicted highly 1N-specific expression pattern (Figure 7).

The flagellar-related clusters included five homologs of phototropin. In *C. reinharditii*, phototropin is found associated with the flagellum and plays a role in light-dependent gamete differentiation [34]. However, phototropin is a light sensor involved in the chloroplast-avoidance response in higher plants [35], so can have roles outside the flagellum. Clusters GS00132, GS01923, and GS00920 showed the highest similarities to the *C. reinharditii *phototoropin sequence (E-values 1 × 10^-22^, 1 × 10^-21^, and 1 × 10^-22^, respectively) and were all only represented in the 1N library (four, four, and three ESTs, respectively). In contrast, GS04170, which showed weaker homology to phototropins (E-value 3 × 10^-9^), was represented by four ESTs in the 2N library and zero from the 1N library. These four clusters all aligned well over the highly conserved LOV2 (light, oxygen, or voltage) domains [35,36] of *C. reinhardtii *and *Arabidopsis thaliana *phototropins (Figure S7 in Additional data file 1). The fifth phototropin homolog, GS01944, was represented by ESTs from both libraries. GS01944 did not correspond to the LOV2 domain. GS00132 and GS00920 were selected for RT-PCR validation, which confirmed that expression of these clusters was indeed highly restricted to 1N cells (Figure 7), as predicted by *in silico *comparison of the two libraries.

We found that several of the selected flagellar-related EST clusters (GS00012, GS04411, GS00844, GS00132 and GS00920) showed a strongly diminished RT-PCR signal in the samples collected during the time of S-phase (Figure 7). Because many genes tested in this study did not display this pattern (for example, GS00217, GS00508, and GS00234), it might be due to real differences in the circadian timing of flagellar gene expression.

#### Use of digital subtraction to identify other 1N- or 2N-specific transcripts

Fourteen of the 199 clusters predicted to be highly 1N-specific and 10 of the 89 clusters predicted to be highly 2N-specific were tested by RT-PCR (Tables 5, 6, 7 and 8; Tables S1 and S2 in Additional data file 7). Twenty-three out of these 24 clusters did show the predicted strong phase-specific expression pattern, confirming that *in silico *subtraction of the two libraries identifies true phase-specific transcripts with a high success rate. Two (the DHC homologs GS00667 and GS00012) were discussed previously and the remaining 22 are discussed in this and the following sections.

##### 1N-specific conserved flagellar-related cluster and 1N-specific possible signal transduction clusters

**Figure 8:**
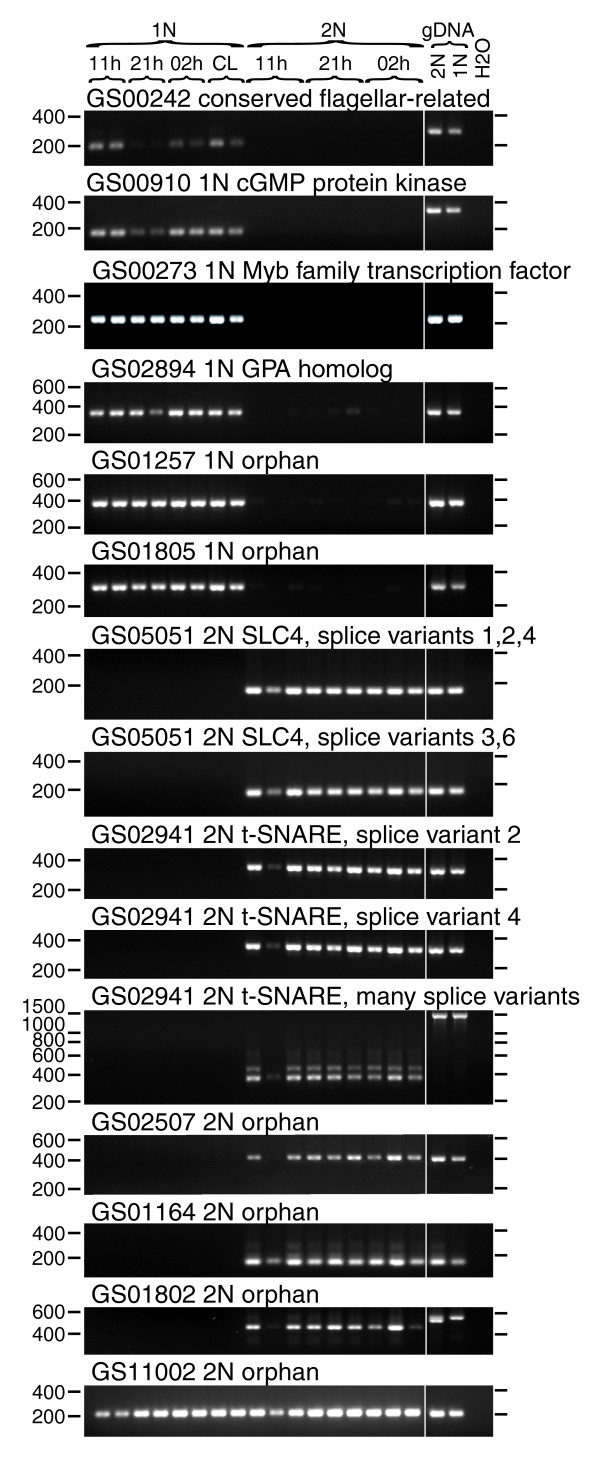
RT-PCR tests of expression patterns of selected genes chosen by digital subtraction. RT- control reactions prepared from the same RNA were run for six of the PCRs shown here and no contaminating genomic DNA (gDNA) was ever found. For clarity, RT- control reactions run simultaneously have been cut out here. Positions of molecular weight markers on each side of the gel are shown. The sample identifiers are listed for each lane at the top of the gel (as for Figure 7).

GS00242 had a moderate level of sequence similarity to the *C. reinhardtii *predicted protein A8J798 (E-value 4 × 10^-14^) and the human spermatogenesis-associated protein SPT17 (E-value 8 × 10^-11^). Although A8J798 is not among the previously confirmed flagellar protein components listed in Table S3 of Pazour *et al*. [27], these authors identified peptides derived from A8J798 in the *C. reinhardtii *flagellar proteome (listed as C-6350001). GS00242 was composed of eight 1N ESTs and zero 2N ESTs. We confirmed by RT-PCR that GS00242 could be detected in 1N RNA samples, but not in 2N RNA samples (Figure 8). GS00910 was classed by KOG as related to cGMP-dependent protein kinases and had a top Swiss-Prot hit to the *Drosophila melanogaster *protein KAPR2, a cAMP-dependent protein kinase type II regularory subunit. It was represented by 14 1N ESTs and 0 2N ESTs and detected by RT-PCR only in 1N RNA samples (Figure 8). The predicted highly 1N-specific expression of two further signal transduction-related clusters (GS00184, a putative protein kinase, and GS00234, a putative calmodulin-dependent kinase) was also confirmed by RT-PCR (Figure S8 in Additional data file 1).

##### 1N-specific Myb homologs

Myb transcription factors control cell differentiation in plants and animals [37-39]. Of the three Myb homologs predicted to be highly 1N-specific, GS00273 was chosen for validation because it had the highest homology to known Myb proteins (*Gallus gallus *c-Myb transcription factor; E-value 3 × 10^-34^). The amino acid sequence derived from GS00273 was readily aligned over the conserved R2-R3 DNA binding regions of Myb family members [37] (Figure S9 in Additional data file 1). RT-PCR confirmed that GS00273 was strongly differentially expressed in 1N cells (Figure 8).

##### 1N-specific cluster GS02894

Cluster GS02894 displayed a sequence similarity to the *E. huxleyi *'glutamic acid-proline-alanine' coccolith-associated glycoprotein (GPA) (E-value 7 × 10^-7^) and was represented by six ESTs from the 1N library and zero from the 2N library. RT-PCR confirmed that GS02894 was highly differentially expressed in 1N cells (Figure 8). Through visual inspection of alignment, we found that GS02894 in fact was aligned poorly with the GPA sequence (Figure S10 in Additional data file 1) and that the alignment did not cover the Ca^2+^-binding loops of the EF-hand motifs previously identified in GPA. GS02894 thus represents a haploid-specific gene product of unknown function.

##### Orphan 1N clusters

GS01257 and GS01805 were orphan clusters highly represented in the 1N library by 25 and 16 ESTs and none in the 2N library in either case (*P *= 1.50 × 10^-8 ^and 7.66 × 10^-6^, respectively). RT-PCR confirmed that both showed highly 1N-specific expression patterns (Figure 8). Both of these clusters showed multiple stop codons in every reading frame, the longest open reading frames on the forward strand being 36 and 35 codons, respectively (not shown). They might represent long 3' untranslated regions (UTRs) of genes that could be successfully identified with full-length sequencing or they might represent transcripts that do not encode proteins.

##### Other highly 1N-specific clusters tested by RT-PCR

A putative β-carbonic anhydrase (GS00157) and a putative cyclin (GS00508) both showed the predicted highly 1N-specific pattern of expression (Figure S8 in Additional data file 1). Two other predicted highly 1N-specific clusters (GS01285 and GS02990) were also confirmed by RT-PCR and are discussed in a later section.

##### 2N-specific SLC4 family homolog

GS05051 was a homolog of the Cl^-^/bicarbonate exchanger solute carrier family 4 proteins (SLC4) [40]. This cluster was represented by seven 2N ESTs and zero 1N ESTs, which comprised six separate mini-clusters that only partially overlapped; these might represent alternative transcripts. Primers designed to separate putative alternative transcripts both detected the expected products from 2N RNA samples but no product from 1N RNA samples in RT-PCR tests (Figure 8), confirming strong differential expression and the existence of alternatively spliced transcripts.

##### 2N-specific SNARE homolog

GS02941, represented by nine 2N ESTs and zero 1N ESTs, was homologous to the SNARE protein family syntaxin-1 involved in vesicle fusion during exocytosis [41]. GS02941 had a top UniProt hit to *Dictyostelium discoidium *Q54HM5, a t-SNARE family protein (E-value 3 × 10^-32^) and a top Swiss-Prot hit to the *Caenorhabditis elegans *syntaxin-1 homolog STX1A (E-value 2 × 10^-19^). RT-PCR confirmed that GS02941 expression was detectable exclusively in RNA from 2N cells using three independent primer sets (Figure 8). The cluster was composed of six different mini-clusters, representing possible different alternative transcripts. Primers designed to mini-cluster e02941.1, one potential alternative transcript form, successfully amplified the predicted 317-nucleotide product but also amplified at least one other product of approximately 400 nucleotides. Only a single approximately 1,500-nucleotide product was amplified from genomic DNA. This suggests that the gene encoding GS02941 contains several (or large) introns that might be subjected to alternative splicing.

##### Orphan 2N clusters

GS02507, GS01164, GS01802, and GS11002 were orphan clusters highly represented in the 2N library with no reads from the 1N library. The longest open reading frames were 171, 309, 236, and 87 amino acids, respectively. GS02507, GS01164, and GS01802 could only be detected from 2N RNA samples, and not at all in 1N RNA samples (Figure 8). In contrast, GS11002 was easily detected in both 1N and 2N RNA samples (Figure 8). PCR amplification of GS01802 from genomic DNA of 2N cells revealed two products, differing by about 50 nucleotides but both larger than the single 444 nucleotides product from cDNA. Only the larger band was visible from 1N genomic DNA. This suggests that two alleles of GS01802 exist in 2N cells, differentiated by the length of an intron, and that only the larger of these alleles was inherited by the clonal 1N cells.

##### Other highly 2N-specific clusters tested by RT-PCR

GS00451 represents a putative aquaporin-type transporter. GS03351 was weakly homologous to a putative arachidonate lipoxygenase previously identified in *E. huxleyi *but to no other proteins in the searched databases, so it may represent a protein of unknown function. Both clusters were confirmed by RT-PCR to be highly 2N-specific (Figure S8 in Additional data file 1). Two other predicted highly 2N-specific clusters, GS00463 and GS02435, are discussed in the next sections.

#### Ca^2+ ^and H^+ ^transport and potential biomineralization-related transcripts

**Figure 9:**
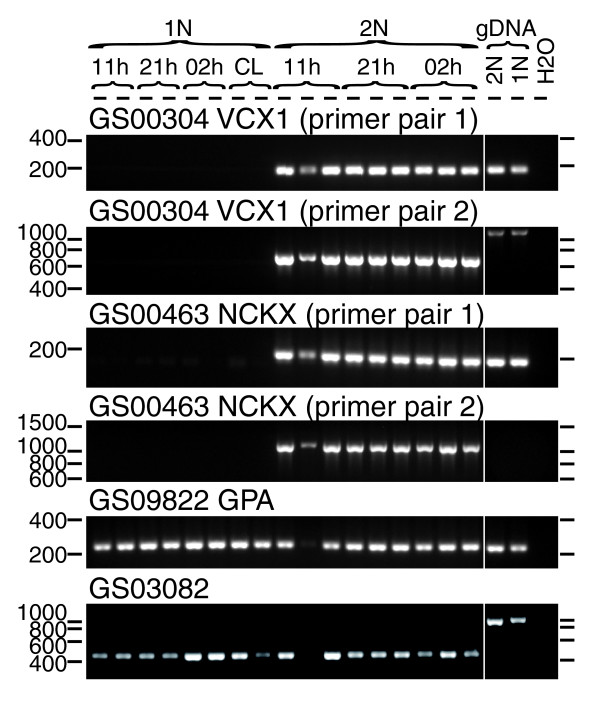
RT-PCR determination of expression patterns of selected genes potentially related to biomineralization. RT- control reactions prepared from the same RNA were run for all of the PCRs shown here and no contaminating genomic DNA (gDNA) was ever found. For clarity, these RT- control reactions run simultaneously have been cut out here. Positions of molecular weight markers on each side of the gel are shown. The sample identifiers are listed for each lane at the top of the gel (as for Figure 7).

We chose to specifically examine Ca^2+ ^and H^+ ^transporters that might play a role in calcification and to determine whether any of them might display highly 2N-specific expression (Table 11). Five clusters had homology to vacuolar-type Ca^2+^/H^+ ^antiporters (VCX1). Although these sequences were aligned with matching regions of known VCX1 proteins at the amino acid level (Figure S11 in Additional data file 1), these clusters could not be well aligned at the nucleotide level (not shown), indicating that they represent paralogs. Only one of these, GS00304, showed possible 2N-specific expression, being represented by four ESTs in the 2N library and zero in the 1N library. GS00304 had a top Swiss-Prot hit to the *A. thaliana *VCX1 homolog CAX2_ARATH (E-value 3 × 10^-60^). We confirmed by RT-PCR that GS00304 was strongly over-expressed in 2N cells using two independent primer sets (Figure 9).

**Table 11:** *E. huxleyi *EST clusters related to Ca^2+ ^and H^+ ^transporters

Cluster ID	Number of 1N clusters	Number of 2N clusters	*P*-value	Top Swiss-Prot hit	E-value
Ca^2+^/H^+ ^antiporter VCX1 and related proteins					
GS00019	7	1	0.020	CAX5_ARATH	2 × 10^-66^
**GS00304**	0	4	0.031	CAX2_ARATH	3 × 10^-60^
GS00617	3	0	0.063	VCX1_YEAST	3 × 10^-58^
GS00976	2	1	0.313	VCX1_YEAST	3 × 10^-31^
GS06500	0	1	0.250	CAX3_ORYSJ	4 × 10^-30^
					
Ca^2+ ^transporting ATPase					
GS07761	1	0	0.250	AT2A2_CHICK	4 × 10^-63^
GS01511	4	5	0.377	ECA4_ARATH	7 × 10^-31^
GS05702	0	1	0.250	ECA4_ARATH	3 × 10^-12^
					
K^+^-dependent Ca^2+^/Na^+ ^exchanger NCKX1 and related proteins					
GS05506	0	2	0.125	NCKX2_RAT	5 × 10^-24^
**GS00463**	0	8	0.002	NCKXH_DROME	1 × 10^-22^
GS04866	2	0	0.125	NCKX_DROME	2 × 10^-22^
GS02609	1	0	0.250	NCKX3_HUMAN	8 × 10^-20^
GS00834	4	3	0.364	NCKXH_DROME	2 × 10^-18^
GS03656	4	1	0.110	NCKX3_MOUSE	6 × 10^-7^
					
Vacuolar H^+^-ATPase V0 sector, subunit a					
GS01798	2	0	0.125	VPP4_HUMAN	2 × 10^-38^
GS02526	1	4	0.109	VPP4_HUMAN	9 × 10^-40^
GS12017	0	1	0.250	No hit	
GS04358	1	0	0.250	VATM_DICDI	2 × 10^-30^
GS08326	0	1	0.250	No hit	
					
Vacuolar H^+^-ATPase V0 sector, subunit c"					
GS01501	4	0	0.031	VATO_YEAST	4 × 10^-47^
					
Vacuolar H^+^-ATPase V0 sector, subunit d					
GS00290	7	5	0.291	VA0D_DICDI	1 × 10^-126^
					
Vacuolar H^+^-ATPase V0 sector, subunit M9.7 (M9.2)					
GS11177	0	2	0.125		
					
Vacuolar H^+^-ATPase V0 sector, subunits c/c'					
GS03783	1	0	0.250	VATL_PLECA	7 × 10^-38^
GS01934	3	5	0.254	VATL_PLECA	4 × 10^-38^
					
Vacuolar H^+^-ATPase V1 sector, subunit A					
GS01727	2	5	0.144	VATA_CYACA	3 × 10^-86^
					
Vacuolar H^+^-ATPase V1 sector, subunit B					
GS08492	0	1	0.250	VATB_ARATH	1 × 10^-62^
					
Vacuolar H^+^-ATPase V1 sector, subunit C					
GS00316	6	4	0.275	VATC1_XENTR	6 × 10^-41^
					
Vacuolar H+-ATPase V1 sector, subunit E					
GS00924	1	1	0.500	VATE_MESCR	1 × 10^-21^
					
Vacuolar H^+^-ATPase V1 sector, subunit F					
GS09780	0	4	0.031	VATF_ARATH	3 × 10^-32^
					
Vacuolar H^+^-ATPase V1 sector, subunit H					
GS01820	1	5	0.062	VATH_MANSE	4 × 10^-36^

Clusters are arranged by KOG hit classification. Clusters in bold were tested by RT-PCR.

Three clusters showed similarity to sarcoplasmic/endoplasmic membrane (SERCA)-type Ca^2+^-transporting ATPases (Table 11). However, none of these clusters showed strong evidence of differential expression by *in silico *comparison of the two libraries.

Six clusters displayed sequence similarities to the K^+^-dependent Na^+^/Ca^2+ ^exchanger (NCKX) family of Ca^2+ ^pumps. These clusters did not align well with each other at the nucleotide level, indicating that they are likely to be distant paralogs (and not alleles). Two of these (GS05506 and GS00463) were only present in the 2N library (two EST reads, *P *= 0.1249, and eight EST reads, *P *= 0.00195, respectively). 2N-specific expression of GS00463 was confirmed by RT-PCR with two independent primer sets (Figure 9).

Homologs of 11 out of the 14 subunits of vacuolar-type H^+^-ATPases were identified, comprising a total of 16 clusters. Seven of these clusters were represented by both 1N- and 2N-ESTs. Only two clusters showed potential differential expression. GS01501 (top Swiss-Prot hit to *Saccharomyces cerevisiae *V-ATPase V0 domain subunit c', E-value 4 × 10^-38^) was present only in the 1N library (four ESTs, *P *= 0.03129) whereas GS09780 (top Swiss-Prot hit to *A. thaliana *V1 domain subunit F) was represented only in the 2N library (four ESTs, *P *= 0.03129). Five clusters were homologous to V0 domain subunit a, the presumed path for proton transport. These clusters did not align at the nucleotide level, thus likely representing distant paralogs (and not alleles). Of the V0 domain subunit a homologs, three shared the highly conserved 20 amino acid motif that contains the R735 residue critical for H^+ ^transport (Figure S12 in Additional data file 1). The other two clusters, each represented by a single EST, were short and did not cover this conserved region. Clusters GS03783 and GS01934 were closely homologous (E-values 7 × 10^-38 ^and 4 × 10^-38^, respectively) to the V0 domain proteolipid subunit (subunit c/c') previously identified as a single-copy gene in the coccolithophore *Pleurochrysis carterae *[42]. These two clusters aligned poorly at the nucleotide sequence level and showed divergence at the amino acid sequence level, thus probably representing paralogs.

The glycoprotein GPA was previously identified to be closely associated with *E. huxleyi *coccoliths by biochemical and immunolocalization studies [43]. Cluster GS09822 was aligned perfectly over its entire length with the amino-terminal 86 codons of the previously sequenced GPA (AAD01505; Figure S10 in Additional data file 1), with minor differences in the 3' UTR (not shown). Surprisingly, GS09822 was represented by one 1N EST and one 2N EST, suggesting expression in both non-calcified 1N cells and calcifying 2N cells, and RT-PCR confirmed that this transcript was abundantly expressed in both calcifying 2N and non-calcifying 1N cells (Figure 9), as predicted from inter-library comparisons.

A previous study identified 45 transcripts with potential roles in biomineralization using microarrays and quantitative RT-PCR comparing expression levels in strain CCMP1516 under phosphate-replete (non-calcifying) and phosphate-limited (weakly calcifying) conditions and in calcifying cells of strain B39 [44]. We attempted to determine whether any of these transcripts might show highly 2N-specific expression patterns (see analysis in Additional data file 10). Of the 45 transcripts in Table 3 of Quinn *et al*. [44], only 23 could be unambiguously identified in public databases based on the provided information and three were each associated with more than one unique EST sequence in GenBank. Fifteen of these transcripts had BLAST matches to clusters in our dataset; ten of these clusters were represented by both 1N and 2N ESTs. Four of the remaining five were represented by only single ESTs from the 2N library. The last cluster, GS03082, similar to GenBank EST sequence DQ658351 from CCMP1516, was composed of two ESTs from the 2N library and zero from the 1N library. However, the transcript for GS03082 was easily detected in RNA from both 1N and 2N cells (Figure 9). Thus, we could not confirm 2N-specific expression of the transcripts described in [44].

#### Possible epigenetic regulation of 1N versus 2N differentiation by histones

**Figure 10:**
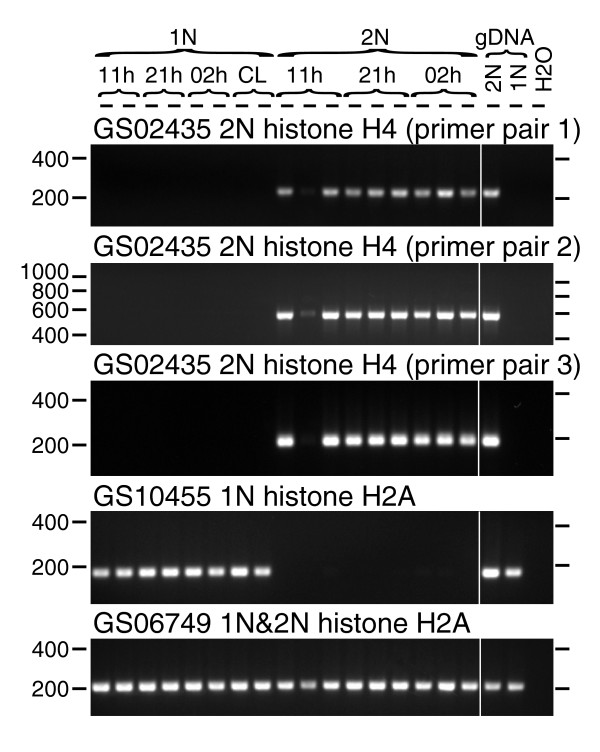
RT-PCR determination of expression patterns of selected histone genes. Positions of molecular weight markers on each side of the gel are shown. The sample identifiers are listed for each lane at the top of the gel (as for Figure 7).

We selected the KOG class 'chromatin structure and dynamics' for closer examination because chromatin packaging might differ between 2N cells and 1N cells as the cells are similar in size but contain different DNA quantities. Also, chromatin factors are known to regulate gene expression. Within this class, two clusters with homology to H4 histones were found to exhibit potential differential expression. GS02435 was composed of six ESTs from the 2N library and zero from the 1N library (*P *= 0.0078). In contrast, GS09138 was composed of 13 ESTs from the 1N library and 0 from the 2N library (*P *= 6 × 10^-5^). A sequence alignment analysis of GS09138 and two other H4 histone homologs (GS07034 and GS07988) showed that these shared high nucleotide identity over the coding region and 100% amino acid sequence identity (Figure S13 in Additional data file 1), suggesting that 1N and 2N cells may preferentially utilize alternative genes for what appear to be the same functional gene product. The 2N-specific GS02435 differed from other H4 histone homologs in the predicted amino acid sequence. The other H4 histone homologs were almost identical along their 103 amino acid predicted length to H4 histones from other eukaryotes but the longest reading frame of GS02435 exhibited an additional ≥ 50 residues in its amino-terminal sequence and lacked 3 carboxy-terminal conserved residues, making this predicted protein at least 27 amino acids longer (by taking the most downstream starting methionine codon) than the typical 103 amino acid residue H4 histones (Figure S13 in Additional data file 1). We confirmed by RT-PCR that GS02435 was detectable only in 2N RNA samples (Figure 10). Surprisingly, genomic DNA-positive controls showed that GS02435 was detected only in 2N genomic DNA and not in 1N genomic DNA (Figure 10). All of the other clusters examined in this study were detected in both 1N and 2N genomic DNA (Figures 7, 8, 9 and 10). The absence of GS02435 from the 1N genome was confirmed by PCR using three independent, non-overlapping PCR primer sets.

There were five clusters with homology to the H2A histone. Alignments of the predicted polypeptides with other eukaryotic H2A histones showed high conservation (Figure S14 in Additional data file 1). GS10455 and GS07154 were identical to each other across the predicted amino acid sequences, although they diverged in nucleotide sequence, particularly in the predicted 5' and 3' UTRs. GS06864 and GS07501 were also identical in predicted amino acid sequence but diverged in nucleotide sequence. GS06749 was divergent from all the other *E. huxleyi *predicted H2A homologs, yet it still grouped well within other eukaryotic histone H2As in preliminary phylogenetic analysis. In particular, it was grouped within the H2A variant class H2AV (Figure S15 in Additional data file 1). GS06749 was composed of four ESTs from the 1N library and three ESTs from the 2N library, and RT-PCR confirmed that it was well-expressed in both 1N and 2N RNA samples (Figure 10). Only one H2A histone homolog, GS10455, showed signs of differential transcription, albeit not statistically significant (two ESTs in the 1N library compared to zero in the 2N library, *P *= 0.1251). We confirmed by RT-PCR that GS10455 was highly expressed in 1N cells with no detection in 2N phase cells (Figure 10).

Two other possible factors in epigenetic control were predicted to be highly 1N-specific. GS01285 had top Swiss-Prot homology to mouse histone H3-K9 methyltransferase 3 (E-value 3 × 10^-13^). However, GS01285 had modestly higher homology scores (1 × 10^-16^) to bacterial ankyrin repeat-containing proteins, so its function is uncertain. Conserved Domains Database (CDD) homology identified a possible DNA N-6-adenine-methyltransferase domain (E-value 4 × 10^-9^) in GS02990. RT-PCR confirmed the prediction that both GS01285 and GS02990 were highly 1N-specific (Figure S8 in Additional data file 1).

## Discussion

### Potential use of the new EST dataset for environmental surveys and understanding the recent evolution of the *Emiliania huxleyi* morpho-species

Two EST datasets were already available from different *E. huxleyi *strains, but in both cases only 2N, day-phase transcripts were represented. The new *E. huxleyi *EST dataset, from 1N and 2N life phases integrated over the day-night cycle, dramatically expands the existing transcriptomic information of this species. The three EST datasets come from strains with widely different geographic origins and morphotypes. The average sequence identity among ESTs from different genetic backgrounds was ≥ 99.5%. Therefore, the limited overlap between the EST sets may be due to physiological or technical differences in the generation of cDNA libraries (for instance, the cDNA libraries here were integrated over the diel and cell cycles, whereas the other cDNA libraries were constructed only from cells harvested during the day, presumably in G1 phase), but is not likely due to sequence divergence within the *E. huxleyi *species-complex. This has several important implications. Practically, this suggests that EST and genomic sequence information from laboratory cultures can be successfully used to design probes for investigating *in situ *gene expression of *E. huxleyi *cells in environmental samples (for example, using microarrays or quantitative RT-PCR). Such probes will be particularly useful as this species frequently dominates phytoplankton communities. Second, the limited sequence variability among strains is consistent with the fossil records, indicating a very recent origin of *E. huxleyi*, which may have rapidly colonized and adapted to a wide range of ocean environments. Limited intra-strain sequence variability suggests that the adaptation perhaps instead involved changes in gene regulation and gain/loss of genes.

### Transcriptome differentiation of haploid and diploid cells

The dramatic phenotypic differentiation between 1N and 2N cells is reflected in the limited overlap between the 1N and 2N EST libraries. Both libraries were normalized, which suppresses highly abundant transcripts to enhance the probability that rare transcripts are sampled. However, the high rate of RT-PCR validation of potentially differentially expressed genes and the fact that homologs of motility-related proteins were distributed exactly as expected according to library origin supports the successful use of *in silico *subtraction of two normalized libraries in this case. The 82 EST clusters related to proteins known to be highly specific for flagella originated exclusively from the 1N library, consistent with the fact that only 1N cells synthesize these structures. In contrast, many clusters homologous to proteins that can have non-flagellar functions originated from both libraries. For example, six of the nine clusters homologous to actin included ESTs from the 2N library. Because of the use of normalization, we focused our analyses on estimates of presence/absence differences and differences in the representation of functional classes of genes, rather than quantitative expression differences of specific genes between the two transcriptomes. Our analysis likely underestimates the true transcriptomic difference between the two cell phases.

The two libraries were estimated to share only 50% of total transcript clusters (by the abundance-based Jaccard similarity index). Such a level of transcriptome differentiation has been seen between mammalian germ and somatic cells [45-47] but it is much greater than that seen in vascular plant germ cell development, where only less than 10% of transcripts in mature male pollen are exclusively expressed in that tissue [48,49].

The estimated transcriptomic richness of 2N cells was approximately 20% larger than that of 1N cells. The same tendency has been seen in our preliminary analysis of 2N versus 1N ESTs from the closely related coccolithophore *Gephryocapsa oceanica *(unpublished data). The 1N cells in this study are clonal. It cannot be ruled out that the 2N cells have undergone sexual recombination since isolation (as clones), because these cells can still produce 1N cells. However, we have never observed 2N cells to be formed in cultures of either clonal 1N cells or non-clonal 1N populations originating from the same 2N clonal parent, suggesting heterothally and/or strong barriers to inbreeding. Thus, we believe the 2N cells have remained clonal and the higher transcriptome richness in 2N cells is not due to increased diversity of genotypes present in these cultures.

The higher transcriptome richness of 2N cells compared to 1N cells has implications for life cycle function in coccolithophores in particular and for life cycle evolution in eukaryotes more broadly. A smaller transcriptome richness in haploid relative to diploid cells was also seen in studies of vascular plant gametophyte development: mature pollen grains express 40 to 50% fewer genes than the diploid progenitor tissues [48,49]. Likewise, the set of genes specifically expressed in post-meiotic spermatids in mammals is smaller than the set of genes specifically expressed in diploid tissues [45], suggesting a similar drop in transcriptome richness in the haploid stage. The large decrease in total expressed genes in the vascular plant pollen grain may mostly reflect that they represent a haploid gametophyte that does not live independently of the parent diploid and is capable of only a limited number of mitoses. A similar explanation would apply for highly specialized short-lived animal sperm that cannot undergo mitosis. This explanation would not apply to haploid coccolithophorid cells, which are capable of unlimited mitotic division and live independently of the diploids. An increase in transcriptome richness with ploidy has not previously been reported in studies of autopolyploid organisms [50,51]. Only one study (done in *S. cerevisiae *using microarrays) has compared global gene expression between haploid and diploid cells where both represent free-living life stages. No decrease in transcriptome richness of 1N cells was observed [50]. Proposed selective advantages allowing the maintenance of haplo-diplontic life cycles in eukaryotes include the ability for each life stage to adapt to alternative 'niches' [52], with 1N stages possibly better adapted to low-resource environments [14,53,54]. Available data on coccolithophore life stages is consistent with this hypothesis, as the holocolith-producing 1N stages of several species are associated with nutrient-poor waters compared to the heterococcolith-producing 2N stages of the same species [18]. Perhaps a reduced transcriptome allows 1N cells to be more streamlined to adapt to specific niches and an intrinsically more rich transcriptome allows 2N cells to be versatile in exploiting a variety of productive environments. There is a tendency of diploid cells to be the dominant building blocks of the most complex multicellular organisms, including animals, vascular plants, and some algae, albeit with many exceptions [13,14]. There might be a more general constraint, such as differential expression of alternative alleles due to heterozygosity, that permits diploid cells to express a larger number of genetic loci (counting alleles as a single entity), and hence a more complex transcriptome, than haploid cells.

### Enhanced motility and sensory systems of 1N cells

The 1N library displayed over-representation of signal transduction-related transcripts compared to the 2N library. This trend was seen when measured by the number of distinct EST clusters, by the number of ESTs, and also by the over-representation of predicted and validated '1N-specific' clusters in the 'signal transduction' functional class. Three clusters related to signal transduction processes were demonstrated to be highly specific to the 1N cells. The motility of 1N cells may require an enhanced repertoire for rapid signal perception and processing, leading to a more sophisticated behavioral repertoire.

A combination of homology analysis and digital subtraction successfully identified a large set of motility-related transcripts in the 1N library. Of the motility-related proteins identified in *C. reinhardtii*, 68% had identifiable homologs in our *E. huxleyi *EST datasets. Likewise, homologs for six of the nine BBS basal body proteins queried were identified, and the identification of 12 distinct flagellar DHC homologs and one cytoplasmic DHC homolog is similar to the number of total flagellar DHCs and cytoplasmic DHCs identified in other organisms [26,55,56]. These proportions are similar to what would be expected from the estimated sampling coverage of the 1N library (Table 4). We conclude that the flagellar elements are highly conserved between *C. reinhardtii *and *E. huxleyi*. Conserved core flagellar structural components, such as flagellar dyneins and basal body components, will probably be intriguing new targets for phylogenomic studies of deep relationships among the eukaryotes, as most eukaryote branches contain flagellated members, and the 'unikont/bikont' split is originally based on flagellar/basal body characters [57].

Motility and sensory systems also appear to account, in part, for the over-representation of clusters with highest homology to metazoans and *C. reinhardtii *among the 1N-unique clusters. *C. reinhardtii *is the only member of the Viridiplantae represented in the KEGG database that has an independently reproducing motile stage. Clusters best-hitting to the moss *Physcomitrella patens *were not over-represented among 1N-unique clusters. Mosses have a flagellated gamete stage but it does not reproduce asexually and moss flagella are lacking components present in *C. reinhardtii *[58]. The gene content differentiation between eukaryotes that have ciliated/flagellated cells in the dominant phase of the life cycle and those that do not is thus reflected in the transcriptome content differentiation between 1N and 2N *E. huxleyi*. This result suggests that the transcriptional differentiation of *E. huxleyi *might involve regulation of functional modules with distinct phylogenetic distribution among diverse eukaryotes.

### Light sensing

The three 1N-specific phototropin homologs might have a role in the pronounced phototaxis exhibited by 1N cells. Although no information is yet available on the environmental cues that might lead to syngamy (fusion of 1N cells to produce 2N cells) or meiosis (formation of 1N cells from 2N cells), light could be an important trigger to examine, given the identification of four phototropin-related genes in this study. Phototropin has been shown to mediate the light-regulation of gamete differentiation, light re-activation of gametes, and zygote development in *C. reinhardtii *[34], and light is also a key regulator of centric diatom gametogenesis [59] and diploid cyst germination in dinoflagellates [60]. The absence of any detectable rhodopsin homologs in the *E. huxleyi *EST database further suggests that phototropin-like proteins might represent the major light-sensing proteins in coccolithophores.

### Identification of new putative elements involved in biomineralization of 2N cells

Coccolith production has been calculated to require a massive sustained flux of Ca^2+ ^from outside the cell into the specialized Golgi-derived coccolith-deposition vesicles where calcification occurs; a major question in coccolithophore cell biology is how the 2N cells can sustain such ion fluxes while avoiding Ca^2+ ^toxicity [61]. The diffusive gradient is very strong for Ca^2+ ^to enter from seawater ([Ca^2+^] ≈ 10 mM) to the cytoplasm (average free [Ca^2+^] << 1 μM), but Ca^2+ ^must then be pumped into the Golgi vesicles against an equally powerful gradient if Ca^2+ ^were to transit through the cytoplasm. We did not identify any candidate 2N-specific Ca^2+^-ATPases, but we found one Ca^2+^/H^+ ^VCX1-type antiporter specific to the 2N calcifying cells. Curiously, we also identified several NCKX family members, at least one of which was highly specific to 2N calcifying cells. NCKX proteins are usually involved in rapid efflux of Ca^2+ ^in cell types that experience frequent large fluctuations in cytoplasmic [Ca^2+^] [62]. If Ca^2+ ^enters immediately into the peripheral endoplasmic reticulum after transiting only a very limited portion of the cytoplasm, as suggested by Berry *et al*. [61], then NCKXs could be important pumps for removing Ca^2+ ^that has leaked into the general cytoplasm from the coccolith deposition vesicle. Alternatively, if coccolith secretion involves Ca^2+^-dependent exocytosis, NCKXs could serve to return cytoplasmic [Ca^2+^] to a resting state after these regular exocytosis events.

Calcification also releases H^+ ^and requires alkalinization of the coccolith deposition vesicles, so cells must simultaneously manage Ca^2+ ^and H^+ ^fluxes [61]. The 2N-specific VCX1 (P-type) Ca^2+^/H^+ ^exchanger might participate in such a function. However, P-type Ca^2+^-stimulated ATPase activity was localized to plasma membrane fractions rather than coccolith vesicle or Golgi vesicle fractions in the coccolithophore *Pleurochrysis *sp. [63]. Vacuolar-type (V-type) H^+ ^ATPase activity has previously been located to the *Pleurochrysis *sp. coccolith vesicle [63] and the V0 subunit c/c' has been immunolocalized there as well [42]. Those studies assumed that the coccolith vesicle must pump H^+ ^into the cytoplasm, functioning in reverse to other eukaryotic V-type H^+ ^ATPases, which pump protons out of the cytoplasm. Here we identified homologs of five out of the six subunits of the V0 domain and six out of the eight subunits of the V1 domain known in yeast. Many of these subunits were represented by multiple homologs but, in general, were found in both the 1N and the 2N libraries; there was no clear candidate for a highly 2N-specific V-type H^+ ^ATPase. This suggests that the same genes are utilized for both forward H^+ ^pumping (out of the cytosol) and possible reverse H^+ ^pumping (into the cytosol from the coccolith vesicle) as there is no obvious reason for the non-calcifying 1N cells to engage in reverse H^+ ^pumping. Alternatively, it may be that VCX1 activity does localize to the coccolith deposition vesicle in *E. huxleyi*, differing from *Pleurochrysis *sp. VCX1-mediated Ca^2+^/H^+ ^exchange has been estimated to occur with a stoichiometry of 3H^+^:1Ca^2+ ^[64,65], whereas calcification releases one H^+ ^for every Ca^2+ ^precipitated [61]. In this scenario, VCX1 would pump excess H^+ ^out of the vesicle and V-type H^+ ^ATPases would function in the normal direction (H^+ ^into the vesicle).

We detected a highly 2N-specific homolog of SLC4 Cl^-^/bicarbonate exchangers, which are well known to play roles in intracellular pH regulation in animal cells [40]. The SLC4 homolog might function to maintain optimal balance of pH and carbonate/bicarbonate in the coccolith deposition vesicle for calcification. None of the 12 carbonic anhydrases identified showed evidence for highly 2N-specific expression in inter-library comparisons. The putative carbonic anhydrase identified with highly 1N-specific expression, GS00157, was a member of the β-family (closest homology to a putative carbonic anhydrase identified in *Pleurochrysis *sp.). Members of this family can localize to either the mitochondria, the chloroplast, or potentially other locations in *C. reinhardtii *[66]. Target prediction was not possible with available EST sequence from GS00157, so its function remains unclear. Identification of the Ca^2+^, H^+ ^and carbonate transport-related transcripts will allow quantitative comparisons of their activities and functions to understand how 2N cells control the large Ca^2+ ^and H^+ ^fluxes involved in calcification.

The 2N-specific SNARE homolog could likely play a role in coccolith secretion, as SNARE proteins are the machinery involved in fusion of secretory vesicles with the plasma membrane during exocytosis [41]. The secretion of coccoliths to the cell surface is a massive and highly coordinated exocytosis event specific to 2N cells: each coccolith is an ellipsoidal plate with axial dimensions ranging from 2 to 4 μm, a substantial fraction of the dimension of an individual *E. huxleyi *cell (4 to 5 μm) [7]. In contrast, 1N cells produce much smaller ellipsoid organic scales with dimensions of 0.4 to 0.6 μm [11].

The glycoprotein GPA was previously found associated with coccolith polysaccharides and displays Ca^2+^-binding activity, so it likely plays a role in biomineralization [43]. However, the GPA gene (GS09822) is well expressed by both calcified 2N cells and non-calcified 1N cells. The set of transcripts hypothesized by Quinn *et al*. [44] to be involved in biomineralization based on differential expression between calcifying and non-calcifying cells (that were presumably 2N) showed a similar pattern. Although Quinn *et al*. did not provide sufficient information to retrieve all of these transcripts from public databases, most of the ones that could be obtained were represented in both of our libraries. Those genes might still be subjected to differential expression or post-transcriptional control. It is also intriguing to speculate that GPA (and perhaps other biomineralization proteins) might have a structural role in non-calcified 1N cells, potentially as a component of 1N-specific organic scales.

### Possible mechanisms for control of the transcriptome

The identification of 1N-specific Myb-like transcription factors and alternative histones provides the first insight into transcriptional and epigenetic regulatory mechanisms in haptophytes. Myb transcription factors are known to control a large variety of cell differentiation processes in plants, including sperm formation [38,39]. Histone variants are differentially expressed in different mammalian tissues [67] and exert epigenetic control in both animals and plants [68,69]. Of the several H2 homologs identified, GS10455 was detected only in 1N cells. It was identical in amino acid sequence to GS07154, expressed in both cells. Both grouped phylogenetically within the canonical H2A histone group. GS06749 was expressed in both 1N and 2N cells yet it was associated phylogenetically with the H2AZ and H2AV variant histones involved in epigenetic controls in plants, yeast, and animals [69]. Therefore GS06749 might be involved in epigenetic controls in *E. huxleyi*. We did not identify a histone H2A variant highly specific to either phase. The potentially 2N-specific H4 histone variant GS02435 is intriguing because H4 histones are nearly 100% identical across all eukaryotes [67,70], yet this H4 variant is unusually long with an extended amino-terminal region with no homology in other eukaryotic H4s. The amino-terminal region of histones, including H4, is the site of specific acetylations and methylations that are thought to influence transcriptional activity of the bound DNA [71]. A strange aspect of GS02435 was its absence from the 1N genome. GS02435 could have been lost from RCC1217 during the culture of this clonal isolate or it could represent a mutated allele present in one of the sets of chromosomes of the 2N genome that was not transmitted to the 1N genome. Alternatively, *E. huxleyi *might be heterothallic (as mentioned above). If the GS02435 gene is on a sex-specific genome segment, half of the 1N cell products of meiosis would not contain it.

## Conclusions

The expanded EST dataset on *E. huxleyi *represents an essential community resource for the on-going annotation and manual curation of the whole genome sequence of *E. huxleyi *and for improved understanding of coccolithophorids and haptophytes. The greatest gap in current understanding of the ecology and natural history of *E. huxleyi *and other coccolithophores is lack of knowledge of the occurrence, distribution, and role of 1N cells. Currently, there are no techniques that can reliably recognize the presence of 1N *E. huxleyi *cells in plankton samples. In addition to revealing potent new hints about specific elements of coccolithophorid cell biology (for example, calcification, motility, and signaling), our dataset will help create useful molecular markers of the occurrence of 1N cells in the field. For example, quantitative RT-PCR using primers specific to *E. huxleyi *flagellar genes might detect the presence of active 1N cells in natural plankton samples.

The heteromorphic haplo-diplontic life cycle and excellent fossil record of coccolithophores makes them an especially attractive group in which to study the influence of the life cycle on gene evolution. An interesting observation was that clusters shared between both life cycle phases were more likely to have homologs among many taxonomic groups whereas clusters unique to one phase or the other were more likely to be orphans. The coming availability of the whole genome sequence of *E. huxleyi *will allow the generation of longer transcript models. This will increase the number of genes for which homologs can be identified, permitting true phylogenomic analyses to track gene origins and more confidence in assignment of genes as orphans. At the same time, genomic and EST resources are being developed for other coccolithophores and haptophytes. It will then be possible to unravel how life cycles influence which genes are maintained or laterally transferred and to test whether new gene families (haptophyte-specific, coccolithophore-specific, or *E. huxleyi*-specific) have tended to arise as specific adaptations for one life cycle phase.

## Materials and methods

### Strain origins and preparation of axenic cultures

*E. huxleyi *strain RCC1216 (= TQ26) was initiated by micropipette isolation of a single cell from a sample from the Tasman Sea off New Zealand in October 1998. A clonal 1N strain (RCC1217) was isolated from RCC1216 following a partial phase change (2N to 1N; Table 1). RCC1217 cultures were made axenic by the following treatments: cells were washed gently on sterile 1.2 μm pore-size filters (Millipore, Billerica, MA, USA) with sterile (autoclaved and 0.2 μm-filtered) seawater, and incubated for 4 days in K/2 (-Tris, -Si) medium [72] mixed with 0.1 volumes Provosoli's antibiotic mix (Sigma-Aldrich P8029, St Louis, Missouri, USA). Cells were re-washed with sterile seawater and allowed to recover in K/2 medium for >4 weeks before washing again and incubating in 50 μg ml^-1 ^ampicillin, 50 μg ml^-1 ^streptomycin dissolved in K/2 medium, incubating for 4 days, and then transferring to antibiotic-free medium. Treatment of RCC1216 (2N) cells was similar but involved first collecting heavily calcified cells on sterile 5 μm pore-size filters (Millipore) before washing, and brief acidification after washing to dissolve calcite coccoliths before antibiotic treatment. Absence of bacteria was confirmed by inoculation in bacterial test media (protocols available at the Center for Culture of Marine Phytoplankton [73]) and by extensive epifluorescence examination of samples fixed in 1% formaldehyde, 0.05% glutaraldehyde, stained with Sybr Green I (Invitrogen, Carlsbad, California, USA), and collected onto 0.2 μm pore-size filters (Millipore). These tests were repeated several times, always in comparison to cultures of the original non-axenic RCC1216 and RCC1217 strains as positive controls. Both inverted light microscopy and flow cytometry confirmed that cultures did not contain any cells of the opposite phase.

### Experimental conditions

Cultures were grown at 17°C, 80 μmol·photons·m^-2 ^on a 14:10 light-dark cycle in a modified K-based medium (iK/5) containing 115 μM nitrate, 20 μM ammonium, 7.2 μM phosphate, trace metals at half the concentration of K/2, and full-strength K/2 vitamins. Analysis of nutrient concentrations showed that cultures had not substantially depleted available nitrogen or phosphorus at time of harvesting and cells thus were in a nutrient-replete state. The moderate light level permits a near maximum growth rate of both 1N and 2N cells without causing photoinhibition of growth seen at higher light levels in 1N cells [15]. A 14:10 light-dark cycle is similar to that experienced during spring blooms and permits phased cell division and an extensive period for dark metabolism to occur. Cultures were not aerated because continuous agitation has been shown to inhibit the growth rate of 1N but not 2N cells of other coccolithophores [74]. Cultures were acclimated to these conditions in semi-continuous culture for 2 weeks prior to the inoculation of 8-L cultures. Mid-exponential phase cells were harvested at eight time points over a 24-h period beginning the first day that cell abundance increased above 10^5 ^ml^-1 ^(11 h, 15 h, 19 h, 21 h, 23 h30 on day 1 and 02 h, 05 h, 08 h on day 2).

### Measurement of cell properties, cell cycle, and photosynthetic efficiency

Cell abundance was measured daily and at each harvesting time point using a Becton-Dickinson FACSort flow cytometer equipped with a 488 nm laser (BD Biosciences, San Jose, California, USA). Cell optical properties were characterized by forward scatter (proportional to protoplast size), side scatter (proportional to optical complexity), and chlorophyll fluorescence. A 3.8% NaCl sheath fluid was used for whole cell measurements to match the refractive index of seawater permitting measurements of forward scatter [75]. Cell cycle phase was determined at each point in harvesting by flow cytometric analysis of Sybr Green I-stained extracted nuclei. Nuclear extraction using previously published protocols [20,76] was successful on 1N cells but not on 2N cells. Photosynthetic efficiency was measured as Fv/Fm with a PhytoPAM pulse-amplitude modulated fluorometer.

### RNA isolation, cDNA library construction, and sequencing

Cells were harvested by filtration onto 1.2 μm pore-size membrane filters (Millipore) and extracted following the Trizol protocol. Ethanol-washed total RNA pellets were resuspended in RNA-free H_2_O, treated with DNase I and further purified using an RNeasy Minikit following the manufacturer's recommendations (Qiagen, Valencia, California, USA). RNA quantity and quality was assessed using a Nanodrop spectrophotometer (Thermo Fisher Scientific, Waltham, Massachussets, USA) and a Bioanalyzer (Agilent Technologies, Inc., Santa Clara, California, USA). The 260:280 ratios were typically greater than 2.2 and absence of degradation was evidenced by sharp 18 s and 28 s bands. Equal amounts of total RNA from each time point were pooled before sending to Vertis Biotechnologies (Freising-Weihenstephan, Germany) for library construction following a protocol for full-length enriched cDNA that includes two successive poly-A RNA purifications, first-strand cDNA synthesis at 42°C for 45 minutes, followed by a ramp up in temperature for a final 10 minutes at 55°C. cDNA was amplified by 19 cycles of PCR. cDNA was normalized by one cycle of denaturation-reassociation followed by separation of reassociated double-stranded cDNA from normalized single-stranded cDNA by hydroxylapatite chromatography. Normalized cDNA was amplified with nine cycles of PCR followed by limited exonuclease digestion. The >0.5 kb size fraction was isolated by agarose gel electrophoresis and directionally ligated into the *Eco*R1 and *Bam*H1 sites of the pBS II sk+ vector. Ligations were electroporated into the T1 Phage resistant TransforMax EC100-T1R (Epicentre Biotechnologies, Madison, WI, USA) electrocompetent *E. coli *cells. Libraries were then sent to Genoscope (Evry, France) for 5' Sanger sequencing.

### EST sequence processing and analysis

Sequences derived from the 5'-end reads were trimmed according to Phred TRIM values [77,78]. Vector, adaptor and polyA sequences were removed using in-house software and the NCBI/UniVec database [79]. The longest high quality regions of each read were used as ESTs. At this stage, we found no obvious contaminations in our EST dataset from other organisms using BLASTN against NCBI/GenBank [80]. ESTs ≥ 50 nucleotides long were selected for further analysis. Initial single-linkage clustering was performed using BLAT version 34 [81] following the criteria that there was ≥ 98% identity across the BLAT alignment and an additional constraint on the alignment (that is, either the alignment was ≥ 150 nucleotides or ≥ 90% of the length of the shorter trimmed read or was long enough that the extremities of the alignment were within a few bases from the end of one of the ESTs in comparison). Next we used the CAP3 program (the version as of December 2007) [82] to generate one or more 'mini-clusters' for each of the initial clusters. A consensus sequence was simultaneously obtained for each mini-cluster. We assigned identifiers (such as 'e00001.1') to mini-clusters. Finally, we performed a third round of clustering based on the overlap of consensus sequences after BLAT mapping on the JGI *E. huxleyi *draft genomic sequences [10] as well as pairwise sequence similarity criteria as above, provided that the latter were consistent with the BLAT genomic mapping data. If the longest consensus sequence of the mini-clusters composing a cluster was shorter than 90 nucleotides, we discarded the corresponding cluster. The clusters finally obtained, each containing one or more mini-clusters, were denoted by distinct identifiers (for example, 'GS00001') in this study. All the EST sequences determined in the present study were deposited in the EMBL database with the assigned accession numbers provided in Additional data file 2. At the final stage of the present study, we noticed a possible contamination of yeast cloning vector (GS12427 with 15 ESTs matching to yeast expression vector pYAA-ZP-MCS EU882163.1). We re-performed BLASTN searches of all mini-cluster consensus sequences against the latest version of GenBank, and found no additional possible contaminations in our EST dataset. Thus, we concluded that possible contaminations did not affect our conclusions in this study. The GS12427 cluster was removed from all lists of clusters in this manuscript but not from Additional data file 2.

EST consensus sequences were searched against the UniProt/Swiss-Prot sequence databases [83] using BLASTX (E-value ≤ 10^-5^), and selected genomes of the KEGG database [84] using BLASTX (E-value ≤ 10^-10^) and against NCBI/KOG [83] and NCBI/CDD using RPS-BLAST (E-value ≤ 10^-5^) after translating EST sequences. For a cluster with multiple mini-clusters (thus, multiple consensus sequences), we recoded the best scoring hit for each of the homology searches against UniProt, Swiss-Prot and KOG. Those clusters with no detectable similarity (E-value ≤ 10^-5^) in these databases were referred to as 'orphans' in this study. We automatically associated *E. huxleyi *EST clusters with *C. reinhardtii *flagellar-related proteins [27,28,30,31], when the EST clusters had a better BLASTX score to one of the flagellar-related proteins than to other predicted protein sequences from the *C. reinhardtii *genome [85]. The automatically generated list of *E. huxleyi *flagellar-related sequences was carefully examined by additional BLAST searches and alignment analysis against specific flagellar or basal body or closely related proteins [26-28,30,31,36,55,56,86-94] (more details are provided in Additional data files 8 and 9).

Transcriptome complexity, or an estimate of the total number of expressed genes represented in each library and in the combined library, was assessed with the Chao1 estimator [24], which has been recommended for estimating microbial diversity in rDNA libraries [95] and has previously been used for analysis of EST libraries [96], and using a ML estimator developed for EST analysis [23]. The ML analysis was performed by artificially dividing the ESTs into two sets based on the time of sequencing (that is, the first and the second rounds of sequencing) and by counting the number of clusters represented by either or both of the two sets. The ML analysis assumes a uniform distribution for the probability of finding an object (in our case, an EST cluster). This assumption may not exactly apply to our dataset, although our ESTs were derived from normalized libraries and the distribution of EST reads per cluster followed a negative exponential curve characteristic of Poisson processes (Figure S3 in Additional data file 1). The ML estimates should thus be considered as qualitative. Chao1 and transcriptome diversity were calculated using EstimateS [97]. Sampling coverage of the libraries was estimated to be >50% for both libraries according to the ratio of total clusters to the estimates of transcriptome richness (Table 4). Slightly lower estimates were obtained using the 'approximately unbiased estimate of coverage' discussed by Susko and Roger [98] (50.4%, 44.8%, and 51.3% for 1N, 2N, and combined libraries, respectively) but the same trends between libraries were seen. Empirical estimates of coverage based on identification of well-known and highly conserved flagellar-related genes were between the two higher coverage estimates, so those are used in Table 4. Shannon diversity H is a function of the total number of genes detected and the distribution of ESTs among the genes [99]. H is maximal when every gene that is detected is represented by an equal number of ESTs: Hmax = lnS (where *S *is the total number of genes that are represented in the library). When H is close to Hmax, it suggests that a new EST generated has a nearly equal probability of being assigned to any of the genes that exist in the library, as expected after normalization. The abundance based estimator of Jaccard similarity index, which estimates the overlap between the 1N and 2N libraries taking sample coverage into account [100], and the Shannon diversity of each library and the combined library were also calculated using EstimateS. Statistical analysis for differential representation between libraries, for both KOG classes and individual clusters, was performed using the method by Audic and Claverie [25]. MUSCLE alignment and phylogenetic analysis werer performed with the web service Phylogeny.fr [101].

### DNA isolation

We filtered 25 ml of dense but growing cultures of RCC1216 (2N) and RCC1217 (1N) onto 25 mm 1.2 μm pore filters (Millipore) and DNA from these were extracted using the Qiagen DNeasy Plant Minikit.

### Primer design, reverse transcription and PCR for confirming gene expression patterns

Oligonucleotide primers were designed using on-line software Primer3 [102] and double-checked for possible self and primer-primer interactions using the on-line Oligo Analysis and Plotting Tool (Operon) [103]. Custom primers were constructed by Eurogentec (Angers, France) and are provided in Table S1 in Additional data file 7.

All RNA samples were diluted to 37.5 ng μl^-1 ^prior to reverse transcription (RT; final reaction concentration 16.9 ng μl^-1^) using the Thermoscript RT-PCR system (Invitrogen) with oligo-dT 20 mers following the manufacturer's protocol with the following temperature selections: RNA was denatured with primer and dNTPs at 65°C for 5 minutes followed by transfer to ice immediately prior to addition of enzyme and buffer. RT was performed at 55°C for 10 minutes, 60°C for 30 minutes, 65° for 10 minutes, and terminated at 85°C for 5 minutes. RT-negative (RT-) reactions were performed in parallel, substituting water for enzyme. Following the RT termination step, samples were treated with RNase following the manufacturer's recommended protocol. All RT+ cDNA and RT- samples were diluted 1:10 prior to testing by PCR. PCRs were performed using the GoTaq PCR Core System I kit (Promega, Madison, Wisconsin, USA) with 1 mM MgCl_2_, 0.2 mM dNTPs, and 0.2 μM of forward and reverse primers. The thermocycler protocol included an initial 2-minute denaturation at 95°C followed by 35 cycles of 45 s denaturation at 95°C, 30 s annealing at 60°C, 90 s extension at 72°C. When preliminary PCR tests showed that the product from genomic DNA was less than 1 kb, the extension was typically shortened to 60 s.

## Abbreviations

BBS: Bardet-Biedl syndrome; CDD: Conserved Domains Database; DHC: flagellar dynein heavy chain; EST: expressed sequence tag; Fv/Fm: maximum quantum yield of photosytem II; GPA: 'glutamic acid-proline-alanine' coccolith-associated glycoprotein; KEGG: Kyoto Encyclopedia of Genes and Genomes; KOG: NCBI eukarote orthologous group; ML: maximum likelihood; NCKX: K^+^-dependent Na^+^/Ca^2+ ^exchanger; RT: reverse transcription; RT-PCR: reverse transcription PCR; SLC4: Cl^-^/bicarbonate exchanger solute carrier family 4; UTR: untranslated region; VCX1: vacuolar-type Ca^2+^/H^+ ^antiporter; V-type: vacuolar-type.

## Authors' contributions

PvD, CdV, and IP together conceived the project. PvD prepared axenic cultures of *E. huxleyi*, managed all experimental work, and wrote the manuscript. IP provided the initial non-axenic *E. huxleyi *cultures and assisted with initial experimental work. PW and CDS managed sequencing at Genoscope. HO, implemented the bioinformatics pipeline for EST processing, clustering, and BLAST searching, and made major contributions at all stages of manuscript preparation. HO, SA, JMC, and PvD performed statistical analyses.

## Additional data files

The following additional data files are available with the online version of this paper: Figures S1 to S15 (Additional data file 1); an Excel-format list of all clusters with component mini-clusters, cDNA clones, and associated EMBL accession numbers (Additional data file 2); an Excel-format list of clusters and mini-clusters with read numbers by library, and top homologies in UniProt, Swiss-Prot, KOG, and CDD (Additional data file 3); an Excel-format list of all clusters predicted by statistical analysis to be 1N-specific, including all orphan clusters and clusters with reads in the 2N library that have *P *< 0.01 associated with the difference in read number between 1N and 2N libraries (Additional data file 4); an Excel-format list of all clusters predicted by statistical analysis to be 2N-specific, including all orphan clusters and clusters with reads in the 1N library that have *P *< 0.01 associated with the difference in read number between 1N and 2N libraries (Additional data file 5); an Excel-format list of selected KEGG genomes used for the taxonomic search (Additional data file 6); a list of all oligonucleotide primers and a summary of RT-PCR results in Tables S1 and S2 (Additional data file 7); a detailed description of identification and analysis of flagellar-related homologs (Additional data file 8); an Excel file spreadsheet indicating the results of automatic queries with *C. reinhardtii *flagellar-related proteins against *E. huxleyi *EST clusters, and an analysis of these clusters compared to the KEGG database (Additional data file 9); an Excel-format spreadsheet detailing results of the search of Table 3 of Quinn *et al*. [44], suspected biomineralization-related transcripts (Additional data file 10).

## Supplementary Material

Additional data file 1 Figures S1 to S15. Click here for file

Additional data file 2 All clusters with component mini-clusters, cDNA clones, and associated EMBL accession numbers. Click here for file

Additional data file 3 Clusters and mini-clusters with read numbers by library, and top homologies in UniProt, Swiss-Prot, KOG, and CDD. Click here for file

Additional data file 4 Clusters predicted by statistical analysis to be 1N-specific, including all orphan clusters and clusters with reads in the 2N library that have *P *< 0.01 associated with the difference in read number between 1N and 2N libraries. Click here for file

Additional data file 5 Clusters predicted by statistical analysis to be 2N-specific, including all orphan clusters and clusters with reads in the 1N library that have *P *< 0.01 associated with the difference in read number between 1N and 2N libraries. Click here for file

Additional data file 6 Selected KEGG genomes used for the taxonomic search. Click here for file

Additional data file 7 Tables S1 and S2 list all oligonucleotide primers and a summary of RT-PCR results. Click here for file

Additional data file 8 Detailed description of identification and analysis of flagellar-related homologs. Click here for file

Additional data file 9 Results of automatic queries with *C. reinhardtii *flagellar-related proteins against *E. huxleyi *EST clusters, and an analysis of these clusters compared to the KEGG database. Click here for file

Additional data file 10 results of the search of Table 3 of Quinn *et al*. [44], suspected biomineralization-related transcripts. Click here for file

## References

[B1] de Vargas C Aubry M-P Probert I Young J Falkowski PG, Knoll AH Origin and evolution of coccolithophores: From coastal hunters to oceanic farmers. Evolution of aquatic photoautotrophs 2007 New York: Academic Press 251 285

[B2] Andersen RA Biology and systematics of heterokont and haptophyte algae. Am J Bot 2004 91 1508 1522 10.3732/ajb.91.10.1508 21652306

[B3] Young JR Geisen M Probert I A review of selected aspects of coccolithophore biology with implications for paleodiversity estimation. Micropaleontology 2005 51 1 22

[B4] Burki F Shalchian-Tabrizi K Pawlowski J Phylogenomics reveals a new 'megagroup' including most photosynthetic eukaryotes. Biol Lett 2008 4 366 369 18522922 10.1098/rsbl.2008.0224 PMC2610160

[B5] Sanchez-Puerta MV Delwiche CF A hypothesis for plastid evolution in chromalveolates. J Phycol 2008 44 1097 1107 10.1111/j.1529-8817.2008.00559.x 27041706

[B6] Thierstein HR Geitzenauer KR Molfino B Global synchroneity of late quaternary coccolith datum levels - validation by oxygen isotopes. Geology 1977 5 400 404

[B7] Paasche E A review of the coccolithophorid *Emiliania huxleyi *(Prymnesiophyceae), with particular reference to growth, coccolith formation, and calcification-photosynthesis interactions. Phycologia 2001 40 503 529

[B8] Iglesias-Rodriguez MD Halloran PR Rickaby REM Hall IR Colmenero-Hidalgo E Gittins JR Green DRH Tyrrell T Gibbs SJ von Dassow P Rehm E Armbrust EV Boessenkool KP Phytoplankton calcification in a high-CO2 world. Science 2008 320 336 340 18420926 10.1126/science.1154122

[B9] Riebesell U Zondervan I Rost B Tortell PD Zeebe RE Morel FMM Reduced calcification of marine plankton in response to increased atmospheric CO_2_. Nature 2000 407 364 367 11014189 10.1038/35030078

[B10] *Emiliania huxleyi *CCMP1516 Main Genome Assembly v1.0 (Joint Genome Institute) http://genome.jgi-psf.org/Emihu1/Emihu1.home.html

[B11] Green JC Course PA Tarran GA The life-cycle of *Emiliania huxleyi*: A brief review and a study of relative ploidy levels analysed by flow cytometry. J Marine Syst 1996 9 33 44

[B12] Klaveness D *Coccolithus huxleyi *(Lohm.) Kamptn. II. The flagellate cell, aberrant cell types, vegetative propagation and life cycles. Br Phycol J 1972 7 309 318

[B13] Coelho SM Peters AF Charrier B Roze D Destombe C Valero M Cock JM Complex life cycles of multicellular eukaryotes: new approaches based on the use of model organisms. Gene 2007 406 152 170 17870254 10.1016/j.gene.2007.07.025

[B14] Mable BK Otto SP The evolution of life cycles with haploid and diploid phases. Bio Essays 1998 20 453 462

[B15] Houdan A Probert I Van Lenning K Lefebvre S Comparison of photosynthetic responses in diploid and haploid life-cycle phases of *Emiliania huxleyi *(Prymnesiophyceae). Mar Ecol Prog Ser 2005 292 139 146

[B16] Mella-Flores D Biologie comparative des cycles de vie chez les coccolithophores. Masters thesis 2007 Université Pierre et Marie Curie, Sciences de l'Universe, Environment et Ecologie

[B17] Frada M Not F Probert I de Vargas C CaCO_3 _optical detection with fluorescent *in situ *hybridization: a new method to identify and quantify calcifying microorganisms from the oceans. J Phycol 2006 42 1162 1169

[B18] Cros L Planktonic coccolithophores of the NW Mediterranean. PhD thesis 2002 Universitat de Barcelona, Departament d'Ecologia

[B19] Frada M Probert I Allen MJ Wilson WH de Vargas C The "Cheshire Cat" escape strategy of the coccolithophore *Emiliania huxleyi *in response to viral infection. Proc Natl Acad Sci USA 2008 105 15944 15949 18824682 10.1073/pnas.0807707105 PMC2572935

[B20] Houdan A Billard C Marie D Not F Sáez AG Young JR Probert I Holococcolithophore-heterococcolithophore (Haptophyta) life cycles: flow cytometric analysis of relative ploidy levels. Systematics Biodiversity 2004 1 453 465

[B21] van Bleijswik JDL Kempers RS Veldhuis MJ Cell and growth characteristics of types A and B of *Emiliania huxleyi *(Prymnesiophyceae) as determined by flow cytometry and chemical analyses. J Phycol 1994 30 230 241

[B22] Wahlund TM Hadaegh AR Clark R Nguyen B Fanelli M Read BA Analysis of expressed sequence tags from calcifying cells of marine coccolithophorid (*Emiliania huxleyi*). Marine Biotechnol 2004 6 278 290 10.1007/s10126-003-0035-3 15136914

[B23] Claverie JM Browne MJ, Thurlby PL Exploring the vast territory of uncharted ESTs. Genomes, Molecular Biology, and Drug Discovery 1996 London: Academic Press 55 71

[B24] Chao A Nonparametric estimation of the number of classes in a population. Scand J Stat 1984 11 265 270

[B25] Audic S Claverie JM The significance of digital gene expression profiles. Genome Res 1997 7 986 995 9331369 10.1101/gr.7.10.986

[B26] Kamiya R Functional diversity of axonemal dyneins as studied in *Chlamydomonas *mutants. Int Rev Cytol 2002 219 115 155 12211628 10.1016/s0074-7696(02)19012-7

[B27] Pazour GJ Agrin N Leszyk J Witman GB Proteomic analysis of a eukaryotic cilium. J Cell Biol 2005 170 103 113 15998802 10.1083/jcb.200504008 PMC2171396

[B28] Stolc V Samanta MP Tongprasit W Marshall WF Genome-wide transcriptional analysis of flagellar regeneration in *Chlamydomonas reinhardtii *identifies orthologs of ciliary disease genes. Proc Natl Acad Sci USA 2005 102 3703 3707 15738400 10.1073/pnas.0408358102 PMC553310

[B29] Ansley SJ Badano JL Blacque OE Hill J Hoskins BE Leitch CC Kim JC Ross AJ Eichers ER Teslovich TM Mah AK Johnsen RC Cavender JC Lewis RA Leroux MR Beales PL Katsanis N Basal body dysfunction is a likely cause of pleiotropic Bardet-Biedl syndrome. Nature 2003 425 628 633 14520415 10.1038/nature02030

[B30] Inglis PN Boroevich KA Leroux MR Piecing together a ciliome. Trends Genet 2006 22 491 500 16860433 10.1016/j.tig.2006.07.006

[B31] Li JB Gerdes JM Haycraft CJ Fan Y Teslovich TM May-Simera H Li H Blacque OE Li L Leitch CC Lewis RA Green JS Parfrey PS Leroux MR Davidson WS Beales PL Guay-Woodford LM Yoder BK Stormo GD Katsanis N Dutcher SK Comparative genomics identifies a flagellar and basal body proteome that includes the *BBS5 *human disease gene. Cell 2004 117 541 552 15137946 10.1016/s0092-8674(04)00450-7

[B32] Ferris PJ Waffenschmidt S Umen JG Lin HW Lee JH Ishida K Kubo T Lau J Goodenough UW Plus and minus sexual agglutinins from *Chlamydomonas reinhardtii*. Plant Cell 2005 17 597 615 15659633 10.1105/tpc.104.028035 PMC548829

[B33] Karki S Holzbaur EL Cytoplasmic dynein and dynactin in cell division and intracellular transport. Curr Opin Cell Biol 1999 11 45 53 10047518 10.1016/s0955-0674(99)80006-4

[B34] Huang KY Beck CF Phototropin is the blue-light receptor that controls multiple steps in the sexual life cycle of the green alga *Chlamydomonas reinhardtii*. Proc Natl Acad Sci USA 2003 100 6269 6274 12716969 10.1073/pnas.0931459100 PMC156361

[B35] Christie JM Salomon M Nozue K Wada M Briggs WR LOV (light, oxygen, or voltage) domains of the blue-light photoreceptor phototropin (nph1): Binding sites for the chromophore flavin mononucleotide. Proc Natl Acad Sci USA 1999 96 8779 8783 10411952 10.1073/pnas.96.15.8779 PMC17593

[B36] Briggs WR Beck CF Cashmore AR Christie JM Hughes J Jarillo JA Kagawa T Kanegae H Liscum E Nagatani A Okada K Salomon M Rudiger W Sakai T Takano M Wada M Watson JC The phototropin family of photoreceptors. Plant Cell 2001 13 993 997 11424903 10.1105/tpc.13.5.993 PMC1464709

[B37] Martin C PazAres J MYB transcription factors in plants. Trends Genet 1997 13 67 73 9055608 10.1016/s0168-9525(96)10049-4

[B38] Ramsay NA Glover BJ MYB-bHLH-WD40 protein complex and the evolution of cellular diversity. Trends Plant Sci 2005 10 63 70 15708343 10.1016/j.tplants.2004.12.011

[B39] Rotman N Durbarry A Wardle A Yang WC Chaboud A Faure JE Berger F Twell D A novel class of MYB factors controls sperm-cell formation in plants. Curr Biol 2005 15 244 248 15694308 10.1016/j.cub.2005.01.013

[B40] Romero MF Fulton CM Boron WF The SLC4 family of HCO3- transporters. Pflugers Archiv Eur J Physiol 2004 447 495 509 10.1007/s00424-003-1180-2 14722772

[B41] Chen YA Scheller RH Snare-mediated membrane fusion. Nat Rev Mol Cell Biol 2001 2 98 106 11252968 10.1038/35052017

[B42] Corstjens PLAM Araki Y Gonzalez EL A coccolithophorid calcifying vesicle with a vacuolar-type ATPase proton pump: Cloning and immunolocalization of the V-0 subunit c(1). J Phycol 2001 37 71 78

[B43] Corstjens PLAM Kooij A van der Linschooten C Brouwers GJ Westbroek P de Vrind-de Jong EW GPA, a calcium-binding protein in the coccolithophorid *Emiliania huxleyi *(Prymnesiophyceae). J Phycol 1998 34 622 630

[B44] Quinn P Bowers RM Zhang YY Wahlund TM Fanelli MA Olszova D Read BA cDNA microarrays as a tool for identification of biomineralization proteins in the coccolithophorid *Emiliania huxleyi *(Haptophyta). Appl Environ Microbiol 2006 72 5512 5526 16885305 10.1128/AEM.00343-06 PMC1538752

[B45] Chalmel F Rolland AD Niederhauser-Wiederkehr C Chung SSW Demougin P Gattiker A Moore J Patard JJ Wolgemuth DJ Jegou B Primig M The conserved transcriptome in human and rodent male gametogenesis. Proc Natl Acad Sci USA 2007 104 8346 8351 17483452 10.1073/pnas.0701883104 PMC1864911

[B46] Kocabas AM Crosby J Ross PJ Otu HH Beyhan Z Can H Tam WL Rosa GJM Halgren RG Lim B Fernandez E Cibelli JB The transcriptome of human oocytes. Proc Natl Acad Sci USA 2006 103 14027 14032 16968779 10.1073/pnas.0603227103 PMC1599906

[B47] Shima JE McLean DJ McCarrey JR Griswold MD The murine testicular transcriptome: Characterizing gene expression in the testis during the progression of spermatogenesis. Biol Reprod 2004 71 319 330 15028632 10.1095/biolreprod.103.026880

[B48] Honys D Twell D Transcriptome analysis of haploid male gametophyte development in *Arabidopsis*. Genome Biol 2004 5 R85 15535861 10.1186/gb-2004-5-11-r85 PMC545776

[B49] Ma J Skibbe D Fernandes J Walbot V Male reproductive development: gene expression profiling of maize anther and pollen ontogeny. Genome Biol 2008 9 R181 19099579 10.1186/gb-2008-9-12-r181 PMC2646285

[B50] Galitski T Saldanha AJ Styles CA Lander ES Fink GR Ploidy regulation of gene expression. Science 1999 285 251 254 10398601 10.1126/science.285.5425.251

[B51] Doyle JJ Flagel LE Paterson AH Rapp RA Soltis DE Soltis PS Wendel JF Evolutionary genetics of genome merger and doubling in plants. Annu Rev Genet 2008 42 443 461 18983261 10.1146/annurev.genet.42.110807.091524

[B52] Valero M Richerd S Perrot V Destombe C Evolution of alternation of haploid and diploid phases in life-cycles. Trends Ecol Evol 1992 7 25 29 10.1016/0169-5347(92)90195-H 21235940

[B53] Adams J Hansche PE Population studies in microorganisms I. Evolution of diploidy in *Saccharomyces cerevisiae*. Genetics 1974 76 327 338 4595645 10.1093/genetics/76.2.327 PMC1213069

[B54] Lewis WM Nutrient scarcity as an evolutionary cause of haploidy. Am Nat 1985 125 692 701

[B55] Asai DJ Koonce MF The dynein heavy chain: structure, mechanics and evolution. Trends Cell Biol 2001 11 196 202 11316608 10.1016/s0962-8924(01)01970-5

[B56] Asai DJ Wilkes DE The dynein heavy chain family. J Eukaryot Microbiol 2004 51 23 29 15068262 10.1111/j.1550-7408.2004.tb00157.x

[B57] Cavalier-Smith T The phagotrophic origin of eukaryotes and phylogenetic classification of Protozoa. Int J Syst Evol Microbiol 2002 52 297 354 11931142 10.1099/00207713-52-2-297

[B58] Merchant SS Prochnik SE Vallon O Harris EH Karpowicz SJ Witman GB Terry A Salamov A Fritz-Laylin LK Marechal-Drouard L Marshall WF Qu LH Nelson DR Sanderfoot AA Spalding MH Kapitonov VV Ren QH Ferris P Lindquist E Shapiro H Lucas SM Grimwood J Schmutz J Cardol P Cerutti H Chanfreau G Chen CL Cognat V Croft MT Dent R The Chlamydomonas genome reveals the evolution of key animal and plant functions. Science 2007 318 245 251 17932292 10.1126/science.1143609 PMC2875087

[B59] Chepurnov VA Mann DG Sabbe K Vyverman W Experimental studies on sexual reproduction in diatoms. Int Rev Cytol 2004 237 91 154 15380667 10.1016/S0074-7696(04)37003-8

[B60] Binder BJ Anderson DM Green light-mediated photomorphogenesis in a dinoflagellate resting cyst. Nature 1986 322 659 661

[B61] Berry L Taylor AR Lucken U Ryan KP Brownlee C Calcification and inorganic carbon acquisition in coccolithophores. Funct Plant Biol 2002 29 289 299 10.1071/PP01218 32689476

[B62] Lytton J Na+/Ca2+ exchangers: three mammalian gene families control Ca2+ transport. Biochem J 2007 406 365 382 17716241 10.1042/BJ20070619

[B63] Araki Y González EL V- and P-type Ca^2+^-stimulated ATPases in a calcifying strain of *Pleurochrysis *sp. (Haptophyceae). J Phycol 1997 34 79 88

[B64] Blackford S Rea PA Sanders D Voltage sensitivity of H+/Ca2+ antiport in higher plant tonoplast suggests a role in vacuolar calcium accumulation. J Biol Chem 1990 265 9617 9620 2351660

[B65] Sze H Liang F Hwang I Curran AC Harper JF Diversity and regulation of plant Ca2+ pumps: insights from expression in yeast. Annu Rev Plant Physiol Plant Mol Biol 2000 51 433 462 11543429 10.1146/annurev.arplant.51.1.433

[B66] Moroney JV Ynalvez RA Proposed carbon dioxide concentrating mechanism in *Chlamydomonas reinhardtii*. Eukaryotic cell 2007 6 1251 1259 17557885 10.1128/EC.00064-07 PMC1951128

[B67] Doenecke D Albig W Bode C Drabent B Franke K Gavenis K Witt O Histones: genetic diversity and tissue-specific gene expression. Histochem Cell Biol 1997 107 1 10 9049636 10.1007/s004180050083

[B68] Bhan S May W Warren SL Sittman DB Global gene expression analysis reveals specific and redundant roles for H1 variants, H1c and H1(0), in gene expression regulation. Gene 2008 414 10 18 18372120 10.1016/j.gene.2008.01.025 PMC2706510

[B69] Deal RB Topp CN McKinney EC Meagher RB Repression of flowering in *Arabidopsis *requires activation of FLOWERING LOCUS C expression by the histone variant H2A.Z. Plant Cell 2007 19 74 83 17220196 10.1105/tpc.106.048447 PMC1820970

[B70] Jaenisch R Bird A Epigenetic regulation of gene expression: how the genome integrates intrinsic and environmental signals. Nat Genet 2003 33 245 254 12610534 10.1038/ng1089

[B71] Phanstiel D Brumbaugh J Berggren WT Conard K Feng X Levenstein ME McAlister GC Thomson JA Coon JJ Mass spectrometry identifies and quantifies 74 unique histone H4 isoforms in differentiating human embryonic stem cells. Proc Natl Acad Sci USA 2008 105 4093 4098 18326628 10.1073/pnas.0710515105 PMC2393763

[B72] Keller MD Selvin RC Claus W Guillard RRL Media for the culture of oceanic ultraphytoplankton. J Phycol 1987 23 633 638

[B73] Center for Culture of Marine Phytoplankton (CCMP) https://ccmp.bigelow.org/

[B74] Houdan A Probert I Zatylny C Véron B Billard C Ecology of oceanic coccolithophores. I. Nutritional preferences of the two stages in the life cycle of *Coccolithus braarudii *and *Calcidiscus leptoporus*. Aquat Microb Ecol 2006 44 291 301

[B75] Cucci TL Sieracki ME Effects of mismatched refractive indices in aquatic flow cytometry. Cytometry 2001 44 173 178 11429767 10.1002/1097-0320(20010701)44:3<173::aid-cyto1109>3.0.co;2-5

[B76] Vaulot D Birrien JL Marie D Casotti R Veldhuis MJW Kraay GW Chretiennotdinet MJ Morphology, ploidy, pigment composition, and genome size of cultured strains of *Phaeocystis *(Prymnesiophyceae). J Phycol 1994 30 1022 1035

[B77] Ewing B Hillier L Wendl MC Green P Base-calling of automated sequencer traces using *Phred*. I. Accuracy assessment. Genome Res 1998 8 175 185 9521921 10.1101/gr.8.3.175

[B78] Ewing B Green P Base-calling of automated sequencer traces using *Phred*. II. Error probabilities. Genome Res 1998 8 186 194 9521922

[B79] NCBI Univec Database http://www.ncbi.nlm.nih.gov.gate1.inist.fr/VecScreen/UniVec.html

[B80] Altschul SF Madden TL Schaffer AA Zhang J Zhang Z Miller W Lipman DJ Gapped BLAST and PSI-BLAST: a new generation of protein database search programs. Nucleic Acids Res 1997 25 3389 3402 9254694 10.1093/nar/25.17.3389 PMC146917

[B81] Kent WJ BLAT - the BLAST-like alignment tool. Genome Res 2002 12 656 664 11932250 10.1101/gr.229202 PMC187518

[B82] Huang X Madan A CAP3: A DNA sequence assembly program. Genome Res 1999 9 868 877 10508846 10.1101/gr.9.9.868 PMC310812

[B83] Consortium U The Universal Protein Resource (UniProt) 2009. Nucleic Acids Res 2009 37 D169 174 18836194 10.1093/nar/gkn664 PMC2686606

[B84] Kanehisa M Araki M Goto S Hattori M Hirakawa M Itoh M Katayama T Kawashima S Okuda S Tokimatsu T Yamanishi Y KEGG for linking genomes to life and the environment. Nucleic Acids Res 2008 36 D480 484 18077471 10.1093/nar/gkm882 PMC2238879

[B85] *Chlamydomonas reinhardtii *Genome Assembly v4.0 (Joint Genome Institute). http://genome.jgi-psf.org/Chlre4/Chlre4.home.html

[B86] Barr FA Gruneberg U Cytokinesis: placing and making the final cut. Cell 2007 131 847 860 18045532 10.1016/j.cell.2007.11.011

[B87] Briggs WR Olney MA Photoreceptors in plant photomorphogenesis to date. Five phytochromes, two cryptochromes, one phototropin, and one superchrome. Plant Physiol 2001 125 85 88 11154303 10.1104/pp.125.1.85 PMC1539332

[B88] Ceulemans H Bollen M Functional diversity of protein phosphatase-1, a cellular economizer and reset button. Physiol Rev 2004 84 1 39 14715909 10.1152/physrev.00013.2003

[B89] Chin D Means AR Calmodulin: a prototypical calcium sensor. Trends Cell Biol 2000 10 322 328 10884684 10.1016/s0962-8924(00)01800-6

[B90] Doble BW Woodgett JR GSK-3: tricks of the trade for a multi-tasking kinase. J Cell Sci 2003 116 1175 1186 12615961 10.1242/jcs.00384 PMC3006448

[B91] Janssens V Goris J Protein phosphatase 2A: a highly regulated family of serine/threonine phosphatases implicated in cell growth and signalling. Biochem J 2001 353 417 439 11171037 10.1042/0264-6021:3530417 PMC1221586

[B92] Morano KA New tricks for an old dog: the evolving world of Hsp70. Ann N Y Acad Sci 2007 1113 1 14 17513460 10.1196/annals.1391.018

[B93] Ni CZ Wang HQ Xu T Qu Z Liu GQ AtKP1, a kinesin-like protein, mainly localizes to mitochondria in *Arabidopsis thaliana*. Cell Res 2005 15 725 733 16212879 10.1038/sj.cr.7290342

[B94] Levy YY Lai EY Remillard SP Heintzelman MB Fulton C Centrin is a conserved protein that forms diverse associations with centrioles and MTOCs in Naegleria and other organisms. Cell Motil Cytoskeleton 1996 33 298 323 8801035 10.1002/(SICI)1097-0169(1996)33:4<298::AID-CM6>3.0.CO;2-5

[B95] Hughes JB Hellmann JJ Ricketts TH Bohannan BJM Counting the uncountable: Statistical approaches to estimating microbial diversity. Appl Environ Microbiol 2001 67 4399 4406 11571135 10.1128/AEM.67.10.4399-4406.2001 PMC93182

[B96] Suga K Welch DM Tanaka Y Sakakura Y Hagiwara A Analysis of expresed sequence tags of the parthenogenetic rotifer *Brachionus plicatilis*. PLoS One 2007 2 e671 17668053 10.1371/journal.pone.0000671 PMC1925144

[B97] Colwell RK EstimateS: Stastical Estimation of Species Richness and Shared Species from Samples. Version 7.5. User's Guide and Application 2005 http://viceroy.eeb.uconn.edu/estimates

[B98] Susko E Roger AJ Estimating and comparing the rates of gene discovery and expressed sequence tag (EST) frequencies in EST surveys. Bioinformatics 2004 20 2279 2287 15059814 10.1093/bioinformatics/bth239

[B99] Shannon CE A mathematical theory of communication. Bell System Technical J 1948 27 379 423

[B100] Chao A Chazdon RL Colwell RK Shen TJ A new statistical approach for assessing similarity of species composition with incidence and abundance data. Ecol Lett 2005 8 148 159

[B101] Dereeper A Guignon V Blanc G Audic S Buffet S Chevenet F Dufayard JF Guindon S Lefort V Lescot M Claverie JM Gascuel O Phylogeny.fr: robust phylogenetic analysis for the non-specialist. Nucleic Acids Res 2008 36 W465 469 18424797 10.1093/nar/gkn180 PMC2447785

[B102] Rozen S Skaletsky HJ Primer3 on the WWW for General Users and for Biologist Programers 2000 Totowa, NJ: Humana Press 10.1385/1-59259-192-2:365 10547847

[B103] Oligo Analysis and Plotting Tool http://www.operon.com

