# Supplementary material for: Transcriptome analysis of functional differentiation between haploid and diploid cells of Emiliania huxleyi, a globally significant photosynthetic calcifying cell
Source: Genome Biol. 2009 Oct 15;10(10):R114. doi: 10.1186/gb-2009-10-10-r114 (PMC2784329; doi:10.1186/gb-2009-10-10-r114)
Supplement: Additional data file 1 — Figures S1 to S15. [file gb-2009-10-10-r114-S1.pdf]

Additional data file 1. Supplementary Figures:

Figure S1. Cell division concentrations in cultures over the day-night cycle during the period of harvesting. Each symbol type represents a separate culture. The x-axis shows time (in hours) since dawn (lights on) on the first day of harvesting. The dark period (lights off) is shown by the grey box. A) Two 1N cultures grown on 14:10 light:dark over the whole experiment. RNA pools from one of these was used in 1N library construction and samples from both were used in RT-PCR tests. B) Four 2N cultures grown on 14:10 LD. RNA pools from one of these was used in 2N library construction and three were chosen at from each time point for RT-PCR testing. C) Two 1N cultures grown on 14:10 LD that experienced a failure of the light regulation one day prior to harvesting, causing them to grown in continuous light. These were not used in library construction but both were used for RT-PCR testing. Only the first 8 time points were used in pools of RNA for library construction and equal quantities of total RNA were mixed to give 100 μg of total RNA in each pool.

Figure S2. Bioanalyzer virtual gel image showing high quality of total RNA. RNA was tested after isolation, DNase treatment, and purification using the Qiagen RNeasy kit RNA clean-up protocol. The distinct 28s, 18s rRNA bands show lack of detectable degradation.

Figure S3. Histogram of EST reads per cluster. Distribution of ESTs across all clusters represented in the 1N library and all clusters represented in the 2N library.

Figure S4. Distribution of clusters by KOG functional class and library for clusters composed of ≥2 EST reads. * indicates KOG classes displaying significant differences between 1N-unique and 2N-unique clusters and @ indicates KOG classes displaying significant differences between 1N-unique and shared clusters.

Figure S5. Taxonomic distribution of clusters "best-hitting" to either Viridiplantae, Stramenopiles, or Metazoa. In each column of numbers, the top row refers to shared clusters, the second row refers to 1N-unique clusters, and the bottom row refers to 2N-unique clusters. Significant differences determined by Fishers Exact Test are marked (*, shared clusters different from both 1N unique and 2N unique; $^{+}$, 1N-unique clusters different from both shared clusters and 2N-unique clusters; ^, 2N-unique clusters different from both shared clusters and 1N-unique clusters).

Figure S6. Example images of full gels showing absence of detectable genomic contamination in RT- samples. Top: GS00217 (Elongation factor 1 α), expressed in both cell types, amplified with primer pair e00217F2, e00217R2. Middle: GS09822 (GPA), expressed in both cell types, amplified with primer pair e09822F2/e09822R1. Bottom, GS02894, strongly differential expression in 1N (amplified with primer pair e02904F1/e02993). RT- reactions were tested in parallel with RT+ reactions with a total of 26 different primer combinations and in no case was a signal detected from RT- reactions.

Figure S7. MUSCLE alignment of phototropin homologs over the LOV2 domain Included also is a sensor hybrid histidine kinase from the planctomycete bacterium *Gemmata obscuriglobus* identified as the second highest hit to the GS00132 predicted amino acid sequence in the nr protein database (E-value = 8e-22). The top hit to GS00132 was a PAS domain-containing predicted protein from *Ostreococcus tauri* (emb|CAL55375.1|) (E-value = 4e-23) but it was less conserved over the LOV2 domain. The following sequences were obtained from Genbank: At-PHOT2 (>gi|145362057|ref|NP_851212.2| PHOT2 (NON PHOTOTROPIC HYPOCOTYL 1-LIKE); kinase [Arabidopsis thaliana]), At-PHOT1 (>gi|15231245|ref|NP_190164.1| PHOT1 (phototropin 1); kinase [Arabidopsis thaliana]), Gemob (>gi|168702150|ref|ZP_02734427.1| multi-sensor hybrid histidine kinase [Gemmata obscuriglobus UQM 2246]). Chlre3 (>estExt_fgenesh2_kg.C_190109 [Chlre3:183965]) was obtained from the *C. reinhardtii* protein catalog.

Figure S8. RT-PCR tests of expression pattern of further genes chosen by digital expression and not shown in Fig. 7-10 of the main test.

Figure S9. MUSCLE alignment of GS00273 and Myb transcription factor family members. The following sequences were obtained from Swissprot: gi|127591|sp|P01103.1|MYB_CHICK, gi|1709195|sp|P52550.1|MYBA_CHICK , gi|417333|sp|Q03237.1|MYBB_CHICK , gi|75336839|sp|Q9S7L2.1|MYB98_ARATH, gi|75336831|sp|Q9S7G7.1|MB3R1_ARATH, gi|127590|sp|P01104.2|MYB_AVIMB. Conserved regularly repeating tryptophan residues are marked with arrows. The fourth tryptophan (the first in the R3 domain) has been substituted conservatively by phenylalanine in GS00273. This tryptophan is often substituted by other aromatic or hydrophobic residues in other plants (Martin and Paz-Ares, 1997).

Figure S10. CLUSTAL alignments of predicted amino acid sequences of clusters with GPA homology against GPA. A) Frame 3 ORF in GS09822 against GPA. B) Longest ORF of GS02894 translated frame 1 against GPA. C) A very strong homology to GPA is found over a 30 amino acid section of translated frame 2 of GS02894 against GPA, yet this frame is interrupted by multiple stop codons (marked in red), including two surrounding a methionine (potential start, marked in green). * marks perfect match, : marks strong conservation, and . marks weak conservation. The predicted EF-hand motif in GPA is underlined. The GPA sequence used was obtained from Genbank (>gi|4102565|gb|AAD01505.1| putative calcium binding protein [Emiliania huxleyi]).

Figure S11. MUSCLE alignment of VCX1 homologs. The following sequences were obtained from Swissprot: VCX1_SCHPO (>gi|74582235|sp|O59768.1|VCX1_SCHPO RecName: Full=Vacuolar calcium ion transporter), VCX1_YEAST (>gi|74623660|sp|Q99385.1|VCX1_YEAST RecName: Full=Vacuolar calcium ion transporter; AltName: Full=Vacuolar Ca(2+)/H(+) exchanger), CAX2_ARATH (>gi|122056116|sp|Q39254.2|CAX2_ARATH RecName: Full=Vacuolar cation/proton exchanger 2), CAX5_ARATH (>gi|75154113|sp|Q8L783.1|CAX5_ARATH RecName: Full=Vacuolar cation/proton exchanger 5).

Figure S12. MUSCLE alignment of partial sequences of V-type ATPase V0a subunits. The highly conserved arginine (R735 in VPH1_YEAST) is marked with an arrow. Two other clusters, GS08326 and GS12017, had hits against the KOG database to V-type ATPase V0a subunits but did not have strong homologs in the Uniprot or Swissprot databases and did not align over the conserved region shown here.The following sequences were obtained from Swissprot: gi|1711568|sp|P37296.2|STV1_YEAST, gi|418296|sp|P32563.3|VPH1_YEAST, gi|3929395|sp|Q01290.1|VPH1_NEUCR, gi|3929385|sp|O13742.1|VPH1_SCHPO, gi|182702220|sp|Q54E04.2|VATM_DICDI, gi|59803038|sp|Q93050.3|VPP1_HUMAN, gi|172046607|sp|Q9Y487.2|VPP2_HUMAN, gi|12643719|sp|Q13488.2|VPP3_HUMAN, gi|38372616|sp|Q9HBG4.1|VPP4_HUMAN, gi|15226542|ref|NP_179736.1| VHA-A2 [Arabidopsis thaliana], gi|30683925|ref|NP_850122.1| VHA-A1 [Arabidopsis thaliana], gi|18420373|ref|NP_568051.1| VHA-A3 [Arabidopsis thaliana], gi|74502607|sp|Q5JDS2.1|VATI_PYRKO [Thermococcus kodakarensis].

Figure S13. MUSCLE alignment of histone H4 homologs. Possible initiator methionines of GS02435 are marked with arrows. The following sequences were obtained from Swissprot: gi|28202123|sp|P59259.2|H4_ARATH [Arabidopsis thaliana], gi|51317339|sp|P62805.2|H4_HUMAN, >gi|74752149|sp|Q99525.1|H4G_HUMA, gi|122107|sp|P02309.2|H4_YEAST, gi|59799579|sp|P69152.2|H42_TETTH [Tetrahymena thermophila]. To increase phylogenetic coverage the following sequences were obtained from the Genbank nr protein dataset: gi|72389584|ref|XP_845087.1| histone H4 [Trypanosoma brucei TREU927], gi|219116983|ref|XP_002179286.1| histone H4 isoform 1b [Phaeodactylum tricornutum CCAP 1055/1], gi|159464912|ref|XP_001690685.1| histone H4 [Chlamydomonas reinhardtii], gi|159480048|ref|XP_001698098.1| histone H4 variant [Chlamydomonas reinhardtii].

Figure S14. Partial MUSCLE alignment of histone H2A homologs. The C terminals, which are extremely variable in length and sequence, are not included. Two mini-clusters that composed cluster GS07501 differed slightly in predicted aa sequence. These are shown by e07501.1 and e07501.2. The following *Arabidopsis thaliana, Homo sapiens, C. reinhardtii*, and *Tetrahymena thermophila* sequences were obtained from Swissprot: gi|75306451|sp|Q94F49.1|H2A5_ARATH, gi|75276926|sp|O04848.1|H2AXA_ARATH, gi|75313113|sp|Q9S9K7.1|H2AXB_ARATH, gi|75308805|sp|Q9C681.1|H2A1_ARATH, gi|75311179|sp|Q9LHQ5.1|H2A2_ARATH, gi|75311051|sp|Q9LD28.1|H2A6_ARATH , gi|75279005|sp|O81826.1|H2A3_ARATH, gi|75309136|sp|Q9FJE8.1|H2A7_ARATH, gi|75311717|sp|Q9LZ46.1|H2A4_ARATH, gi|75313476|sp|Q9SII0.1|H2AV2_ARATH, gi|75277395|sp|O23628.1|H2AV1_ARATH, gi|75308904|sp|Q9C944.1|H2AV3, gi|75314165|sp|Q9T0H7.1|H2A8_ARATH, gi|74752099|sp|Q96QV6.3|H2A1A_HUMAN, gi|74750623|sp|Q8IUE6.3|H2A2B_HUMAN, gi|121992|sp|P16104.2|H2AX_HUMAN, gi|12643341|sp|Q93077.3|H2A1C_HUMAN, gi|47117890|sp|Q16777.4|H2A2C_HUMAN, gi|74733131|sp|Q9BTM1.1|H2AJ_HUMAN, gi|74751984|sp|Q96KK5.3|H2A1H_HUMAN,

gi|74757558|sp|Q6FI13.3|H2A2A_HUMAN, gi|12585257|sp|Q99878.3|H2A1J_HUMAN, gi|83288406|sp|P0C0S8.2|H2A1_HUMAN, gi|74749897|sp|Q7L7L0.3|H2A3_HUMAN, >gi|121978|sp|P20671.2|H2A1D_HUMAN, gi|124028530|sp|P04908.2|H2A1B_HUMAN, gi|90110023|sp|O75367.4|H2AY_HUMAN, gi|12585260|sp|Q9P0M6.3|H2AW_HUMAN, gi|74749787|sp|Q71UI9.3|H2AV_HUMAN, gi|83288408|sp|P0C0S5.2|H2AZ_HUMAN, gi|161784333|sp|P0C5Z0.1|H2AB2_HUMAN, gi|161784332|sp|P0C5Y9.1|H2AB1_HUMAN, gi|1708102|sp|P50567.1|H2A_CHLRE, gi|462229|sp|P35064.2|H2A1_TETTH, gi|462230|sp|P35065.2|H2A2_TETTH, gi|121991|sp|P08992.2|H2AV_TETTH. Four diatom sequences were obtained from *Thalassiosira pseudonana* and *Phaeodactylum tricornutum* via the Genbank nr protein database. H2A_Tpseud (gi|209585850|gb|ACI64535.1|), H2A-1_Ptric (gi|219122004|ref|XP_002181345.1| histone H2A isoform 1), H2A-2_Ptric (gi|219119185|ref|XP_002180358.1| histone H2A isoform 2), H2A-3_Ptric (gi|219116815|ref|XP_002179202.1| histone H2A isoform 3a).

Figure S15. Phylogenetic tree classifying different H2A homologs. MUSCLE-aligned sequences were curated with GBLOCKS and aligned using the PhyML method and the Approximate Likelihood Ratio Test. Branch nodes with support <50% have been collapsed.

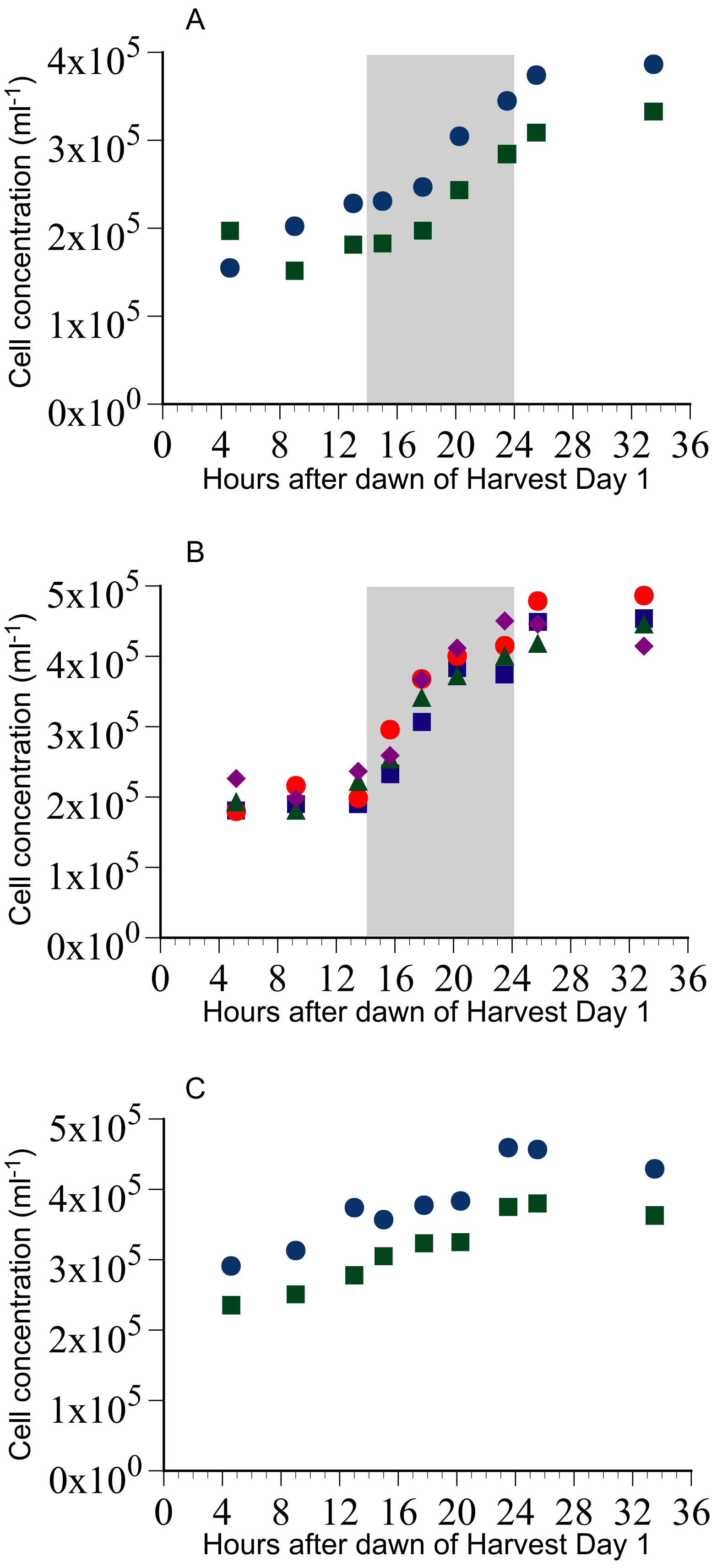

Supplementary Figure S1.

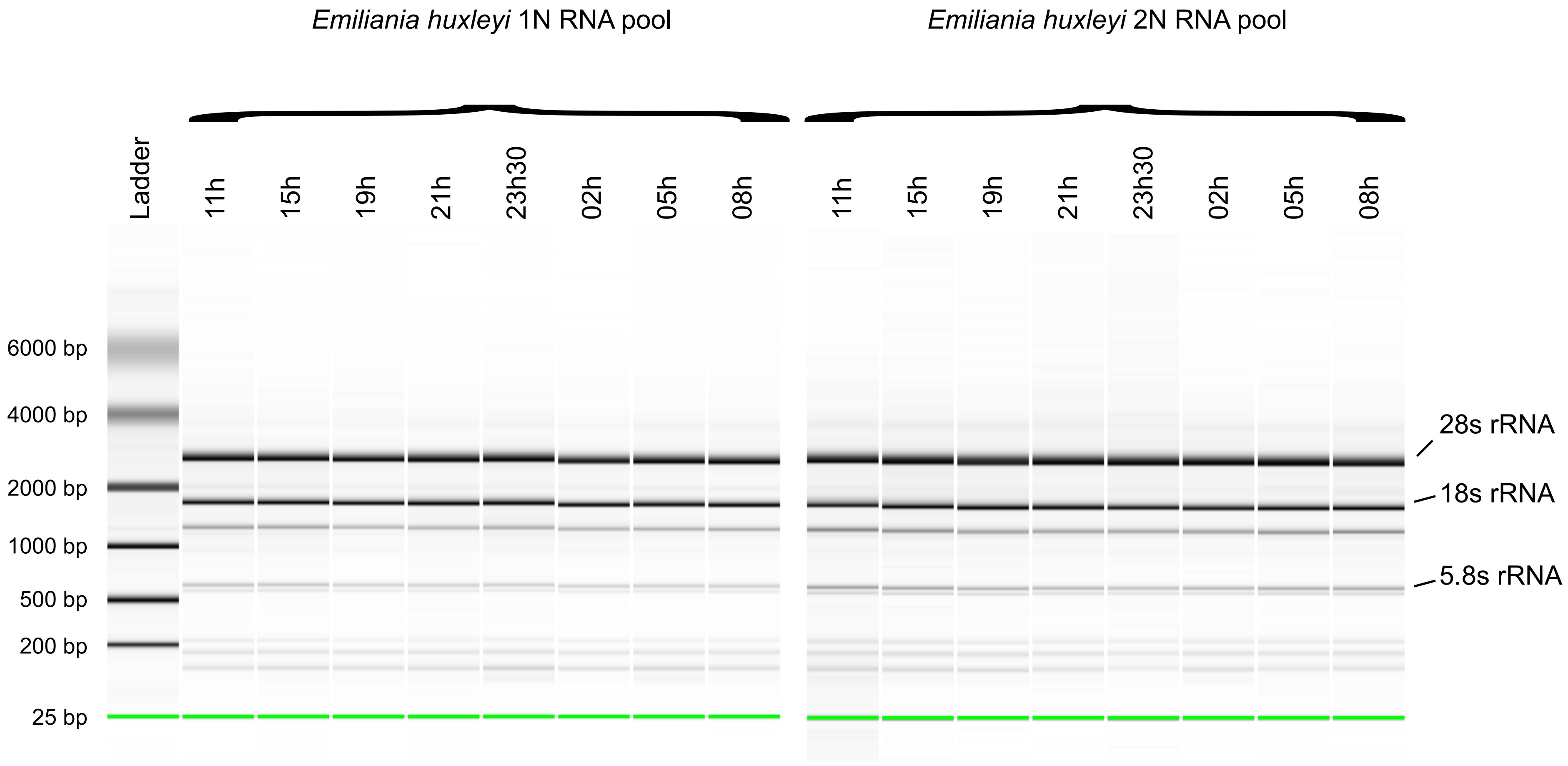

Supplementary Figure S2. Bioanalyzer analysis of total purified RNA samples pooled for construction of 1N and 2N libraries.

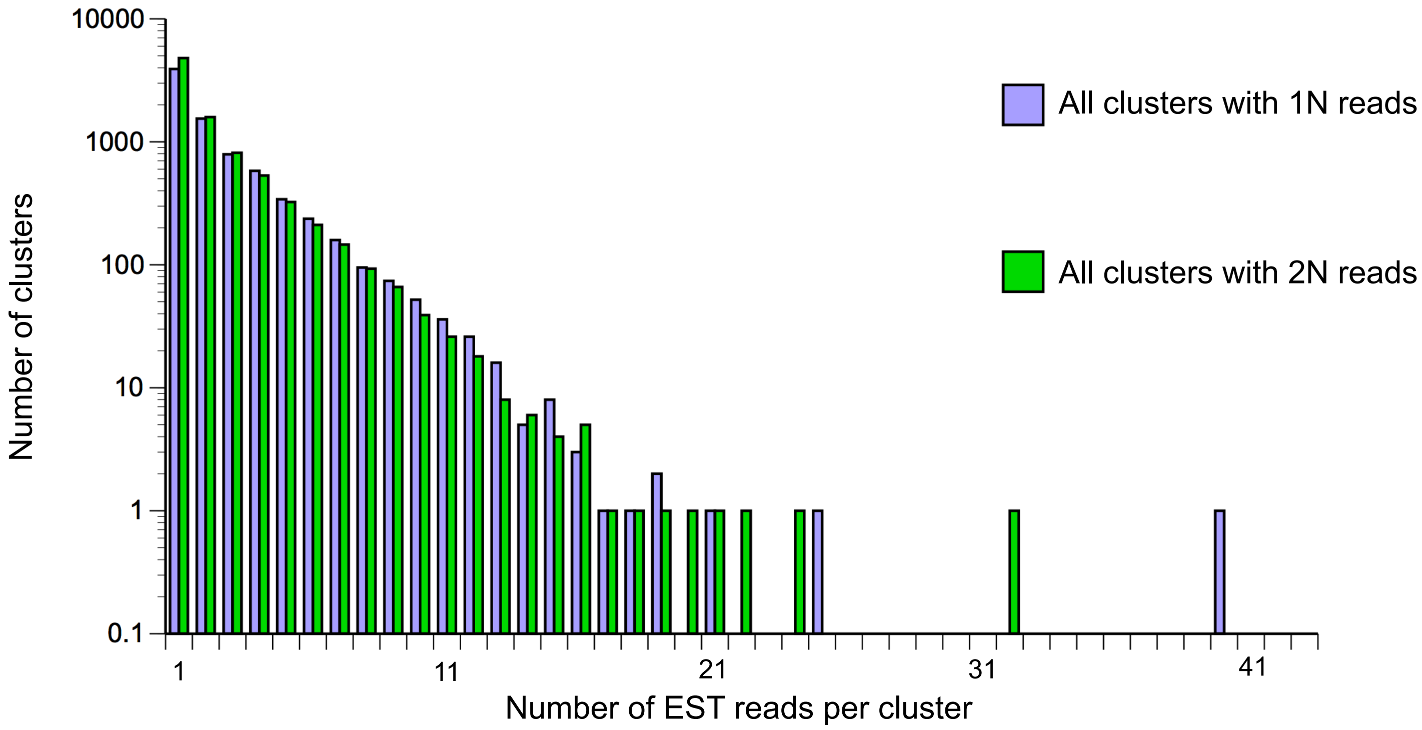

Supplementary Figure S3.

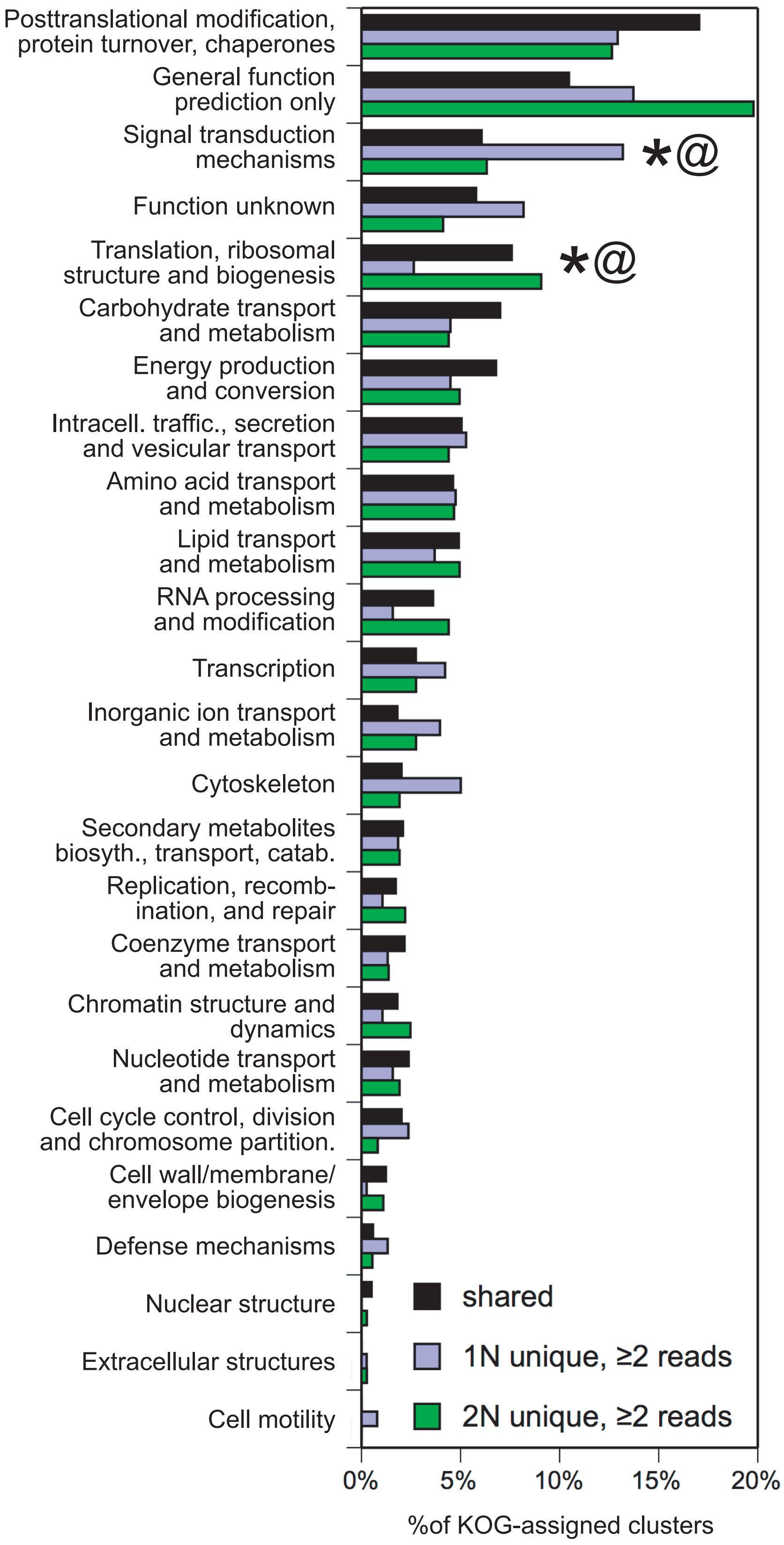

Supplementary Figure S4.

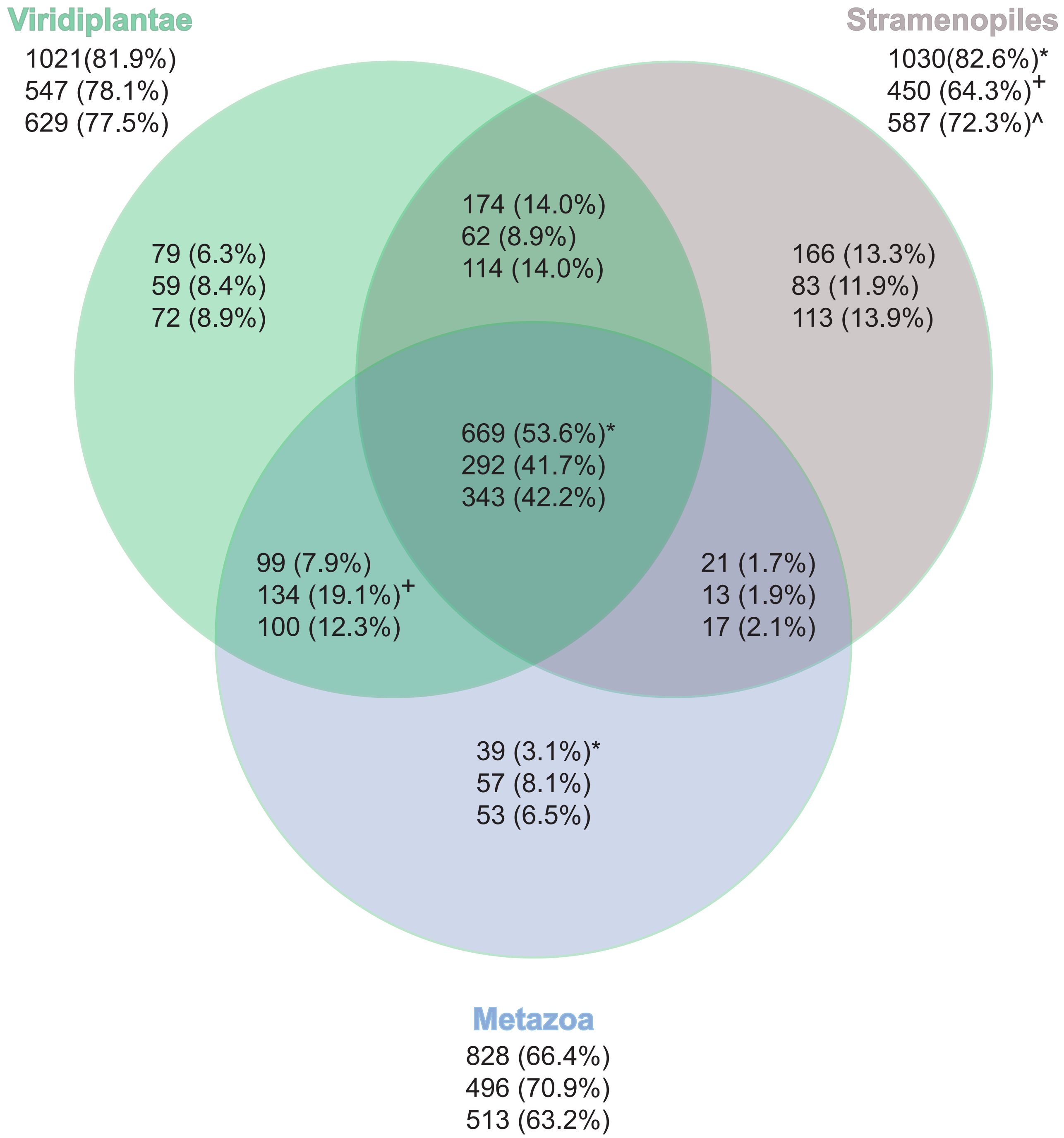

Supplementary Figure S5.

GS00217 elongation factor 1α, primer set 2

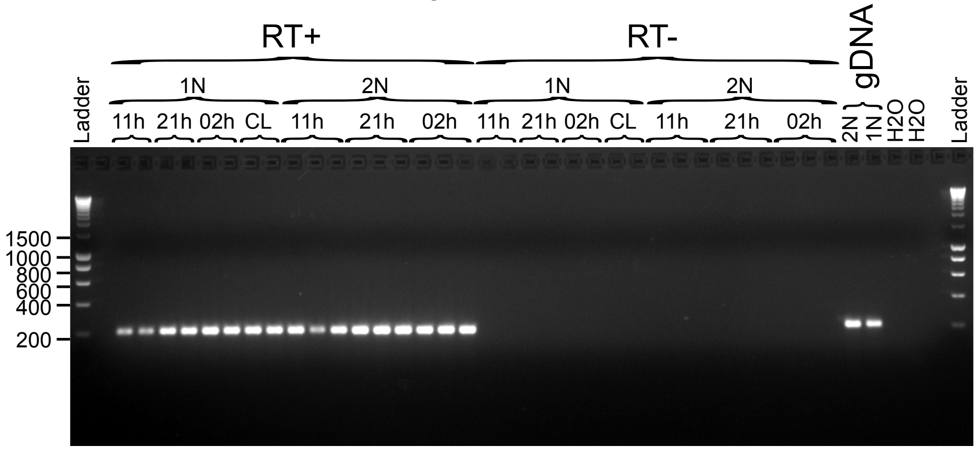

GS09822 GPA

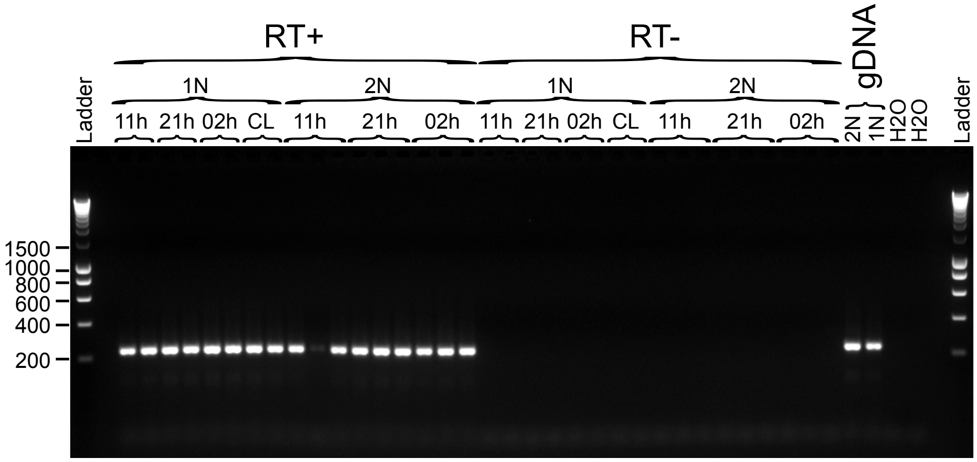

GS02894 1N-specific false GPA homolog

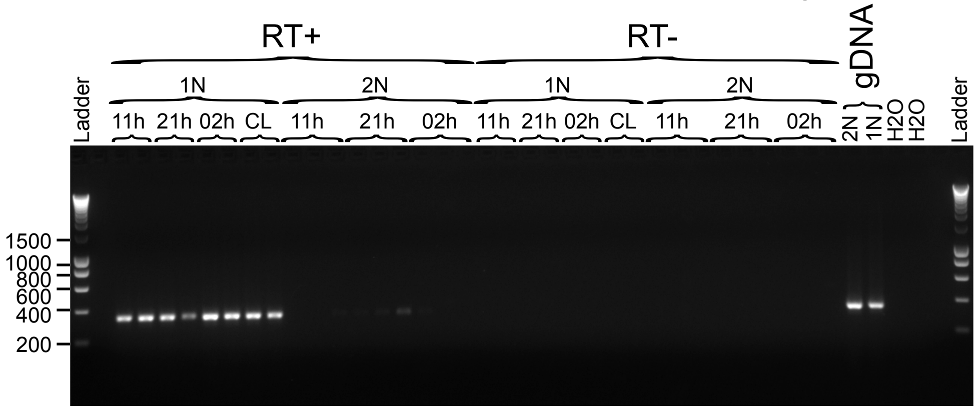

GS00844 BBS5 (primer pair 2)

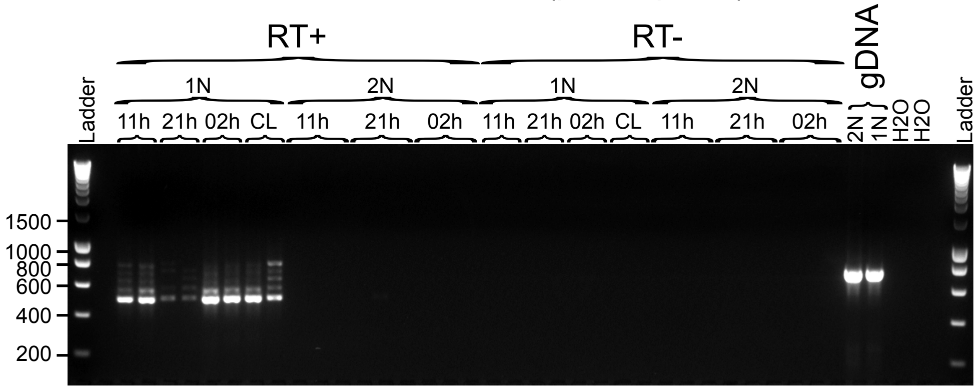

Supplementary Figure S6.

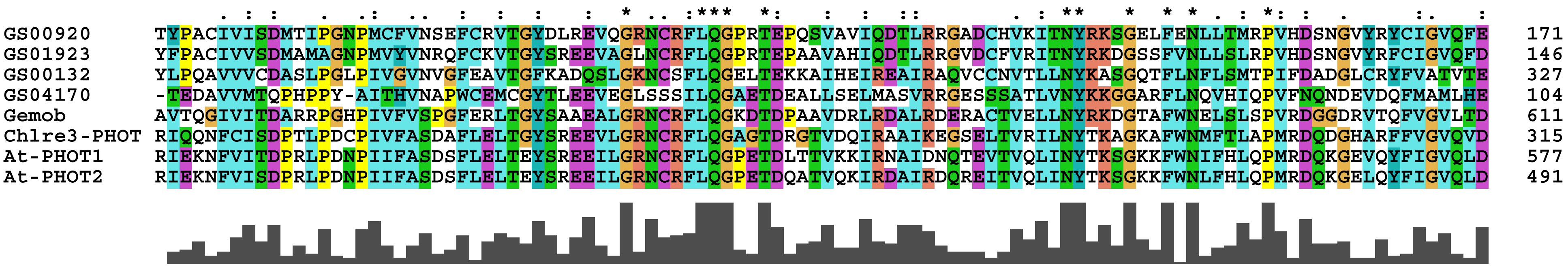

Supplementary Figure S7.

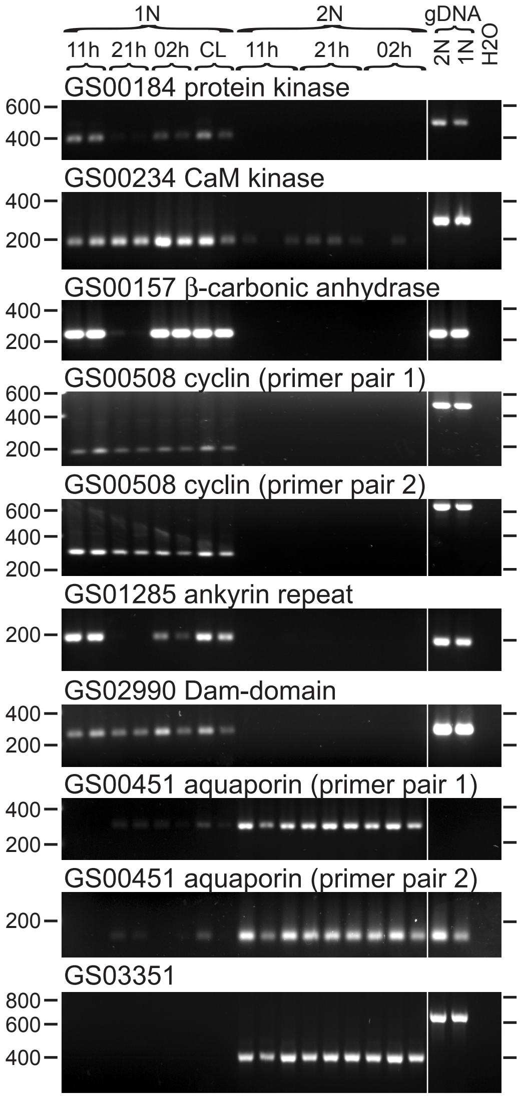

Supplementary Figure S8.

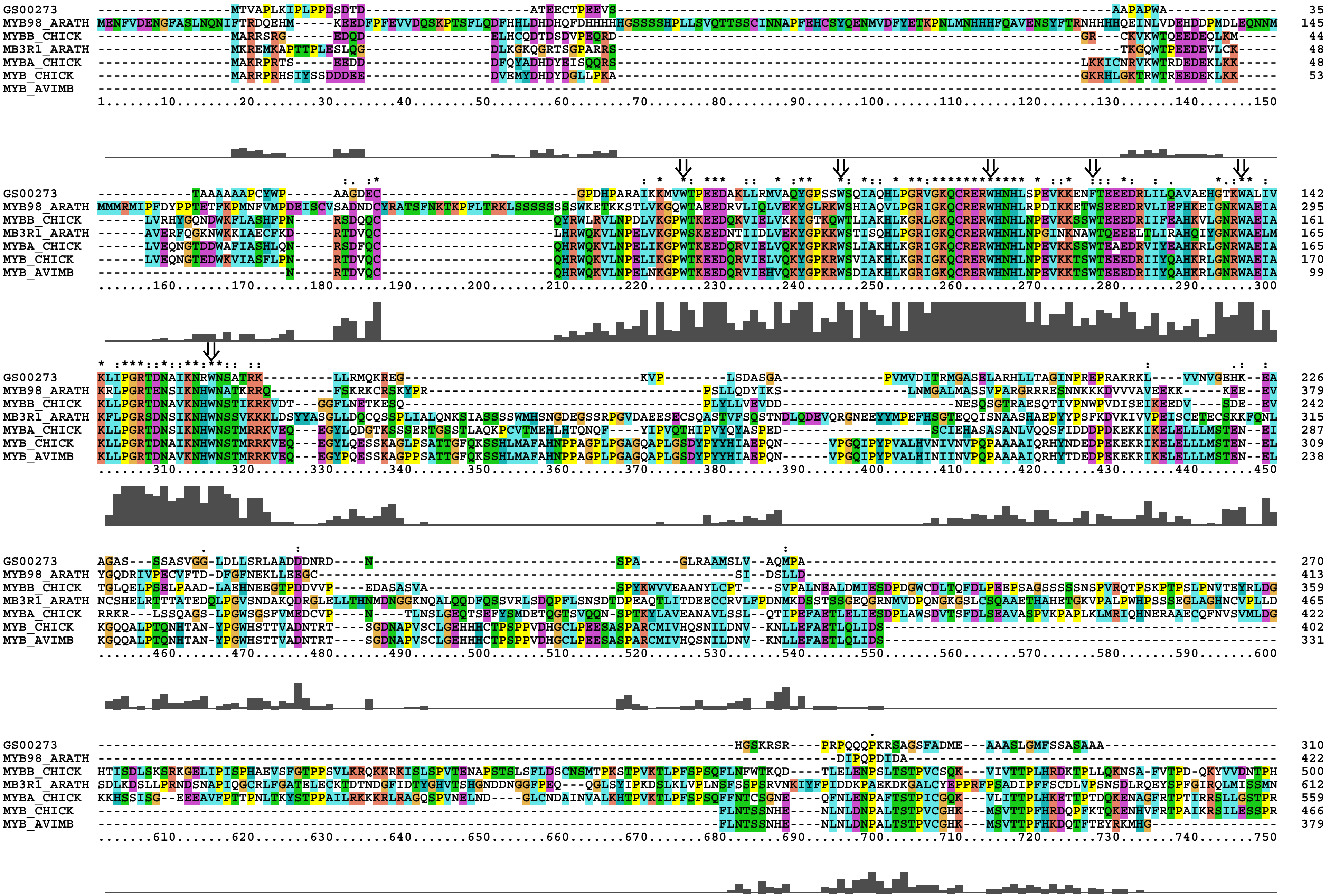

Supplementary Figure S9.

A

```
GS09822         ------------------------------------------------------------
GPA             MGVCSSTPKDQPVAEPTYDPAPAPAPEVPEVPEVPEGAVDTAIDKLETKVGSDLDGDGQV

GS09822         ------------------------------------------------------------
GPA             AGVPVEEVPADAPAEAADAVEPAEDKNILQKAADYVKSFSGRVEPEPAAEEPAVEPEAAP

GS09822         ------------------------------------------------------------
GPA             EGGYEAPKEEASAEEAPAEEAPEPEPETASFLDKVKSYLSPRPEVPEGAVDTAIDKLETK

GS09822         ------------------------------------------------------------
GPA             VGSDLDGDGEVAGVPVEAAAEEVEAATEAAAEEVPVEAAAEAVEAAVEAAPAVPEKKESY

GS09822         -----------------------------------EPVAEAEAEAAPEVAPEAEEEKPSL
GPA             LGKVASILSPRKKAASDASAADAPAADDAAEAAEAEPVAEAEAEAAPEVAPEAEEEKPSL
                                                   *************************

GS09822         IQKLSKAFSSIIPATPQCLPGRPSESTLNASETSADAPSAAEEEEEAAPAYKAEAPAEEVK
GPA             IQKLSKAFSSIIPATPQCLPGRPSESTLNASETSADAPSAAEEEEEAAPAYKAEAPAEEVK
                ************************************************************

GS09822         A
GPA             A
                *
```

B

```
GS02894frame1   MGCSSGK---------MATAEPATALHLRTMGTCSSKSINNTIADPVKPEQIDTPADATI
GPA             MGVCSSTPKDQPVAEPTYDPAPAPAPEVPEVPEVPEGAVDTAIDKLETKVGSDLDGDGQV
                ** .*..            . **.* .:  :   .. :::.:* .  .    *  .*. :

GS02894frame1   VDATTDIAPTEMPADAAP------------------------------------------
GPA             AGVPVEEVPADAPAEAADAVEPAEDKNILQKAADYVKSFSGRVEPEPAAEEPAVEPEAAP
                .....: .*:: **:**

GS02894frame1   -SDYAKPEAEVAVAEEPEALG------SAAFLWERLGFLTR-------------------
GPA             EGGYEAPKEEASAEEAPAEEAPEPEPETASFLDKVKSYLSPRPEVPEGAVDTAIDKLETK
                 ..*  *: *.:. * *   .      :*:** :  .:*:

GS02894frame1   ------------------------------------------------------------
GPA             VGSDLDGDGEVAGVPVEAAAEEVEAATEAAAEEVPVEAAAEAVEAAVEAAPAVPEKKESY

GS02894frame1   ---------------------SAEAAPEAAPEAAPEVAVEAAPEVAVEAAPEAAVEEALA
GPA             LGKVASILSPRKKAASDASAADAPAADDAAEAAEAEPVAEAEAEAAPEVAPEAEEEKPSL
                                     .* ** :**  * .* ..** .*.* *.****  *:.

GS02894frame1   VHHLDR------------------------------------------------------
GPA             IQKLSKAFSSIIPATPQCLPGRPSESTLNASETSADAPSAAEEEEEAAPAYKAEAPAEEVK
                :::*.:

GS02894frame1   -
GPA             A
```

Supplementary Figure S10.

C

```
GS02894frame2   -------------------------------KEVSQDVVCAGFGCLVX------------
GPA             MGVCSSTPKDQPVAEPTYDPAPAPAPEVPEVPEVPEGAVDTAIDKLETKVGSDLDGDGQV
                                                **.:..* :.:. *

GS02894frame2   ------------------FCTPLFAGNLWAVRAERWRPPNRRLLFIYEPWVPAAPSRSTT
GPA             AGVPVEEVPADAPAEAADAVEPAEDKNILQKAADYVKSFSGRVEPEPAAEEPAVEPEAAP
                                     *    *:    *:  :. . *:     .  **. ..::.

GS02894frame2   PSPIRXSRSRSTLPQTPRSSTPRPTSRLPRCPPMRRPRITQSPRQRX-------------
GPA             EGGYEAPKEEASAEEAPAEEAPEPEPETASFLDKVKSYLSPRPEVPEGAVDTAIDKLETK
                 .  . .:..::  ::* ..:*.* .. .      :. ::  *.

GS02894frame2   ---------RWQRSPRRSAQPRSCGSASASSHGLLRPLLRPLLRLRPRWRLRLRPR---W
GPA             VGSDLDGDGEVAGVPVEAAAEEVEAATEAAAEEVPVEAAAEAVEAAVEAAPAVPEKKESY
                         .    * .:*  .  .::.*::. :        :.   .    :  :   :

GS02894frame2   RLRLRPRLRSRRRSRSITWIANVIKAGSFVVDGEHTPELIDEVDE--------EEDKPTL
GPA             LGKVASILSPRKKAASDASAADAPAADDAAEAAEAEPVAEAEAEAAPEVAPEAEEEKPSL
                  :: . * .*::: * :  *:.  *.. .  .*  *    *.:         **:**:*

GS02894frame2   VQKLSKAFSNILPAAPKCMPSHP-----ELNEEPADAPTAAXSRQVCVCAVAVIXTYQCL
GPA             IQKLSKAFSSIIPATPQCLPGRPSESTLNASETSADAPSAAEEEEAAPAYKAEAPAEEVK
                :********.*:**:*:*:*.:*     : .* .****:** ..:.. .  *   : :

GS02894frame2   LC
GPA             A-
```

Supplementary Figure S10 (continued).

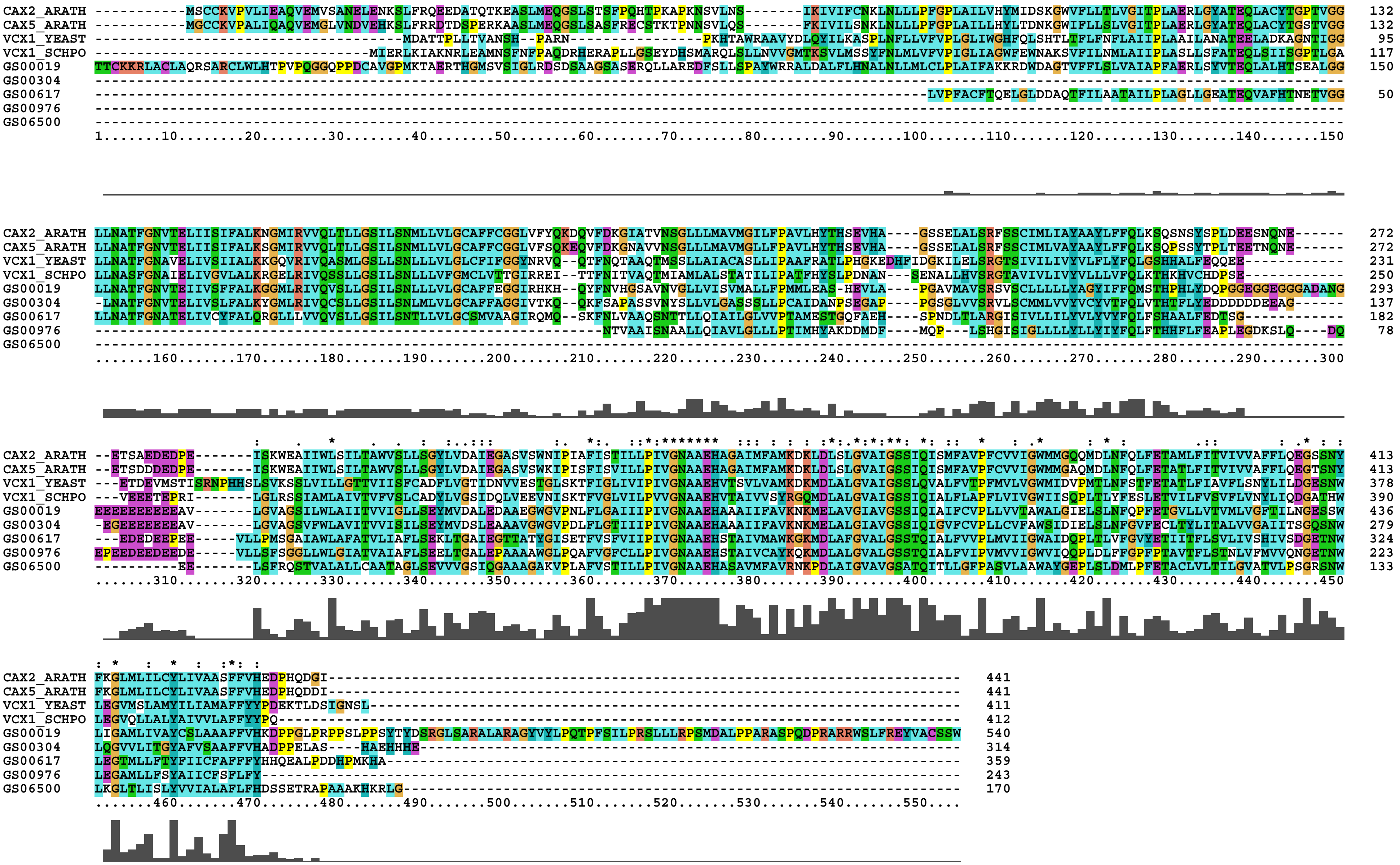

Supplementary Figure S11.

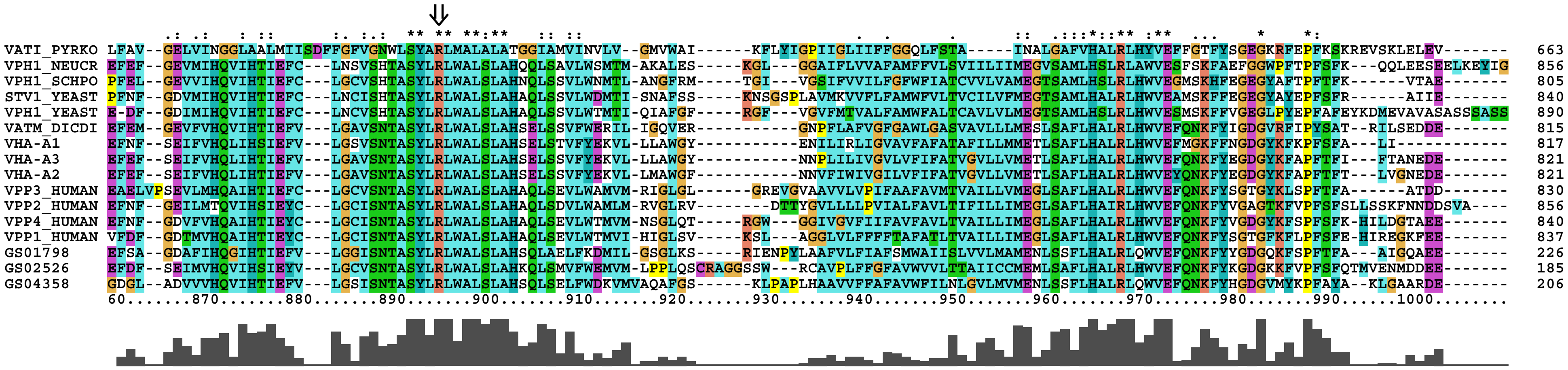

Supplementary Figure S12.

```
GS02435          PHTTSQYMATMALLPLSVLAMAPALLPCDPEPLSAQLAGTAASPDDHADVQTLDRLMSANLDPDFKASLFLGSDYVEGSLELDDAVWQVAQAAAEYALATMFSDAASILEKEPTLFATPSEAADDQF---DKAFKATAFLFKEEKGLKKT  147
GS09138          ------------------------------------------------------------------------------------------------------------------------MSGRGKG---GKGLG---------KGGAKR   18
GS07034          ------------------------------------------------------------------------------------------------------------------------MSGRGKG---GKGLG---------KGGAKR   18
GS07988          ------------------------------------------------------------------------------------------------------------------------MSGRGKG---GKGLG---------KGGAKR   18
H4_ARATH         ------------------------------------------------------------------------------------------------------------------------MSGRGKG---GKGLG---------KGGAKR   18
H4_Crein         ------------------------------------------------------------------------------------------------------------------------MSGRGKG---GKGLG---------KGGAKR   18
H4variant_Crein  ------------------------------------------------------------------------------------------------------------------------MSGIDKCLGPGRGIG----------KGGKR   20
H4_HUMAN         ------------------------------------------------------------------------------------------------------------------------MSGRGKG---GKGLG---------KGGAKR   18
H4G_HUMAN        ------------------------------------------------------------------------------------------------------------------------MSVRGKA---GKGLG---------KGGAKC   18
H4_YEAST         ------------------------------------------------------------------------------------------------------------------------MSGRGKG---GKGLG---------KGGAKR   18
H42_TETTH        ------------------------------------------------------------------------------------------------------------------------MAG-GKG---GKGMG---------KVGAKR   17
H41b_Ptric       ------------------------------------------------------------------------------------------------------------------------MSGRGKG---GKGLG---------KGGAKR   18
H4_Tbruc         ------------------------------------------------------------------------------------------------------------------------MAKGKKS---GEAKG-----------SQKR   16
                 1.......10........20........30........40........50........60........70........80........90.......100.......110.......120.......130.......140.......150
```

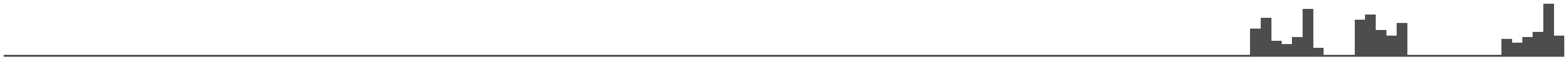

```
                  .    .:.  .:*:  :*****  :*****:  ..:*::  *  *::  *:*  ::    :  :  **:::::  *.  :  *:  .*:::*:  *
GS02435          TAGTAPSENIQ-GITKPAIRRLADRGGVKRISGLIYEETRGVLKVFLENVIRDAVTYTEHARRKTVTAMDVVYALKRQGRTLYR----  230
GS09138          H-RKVLRDNIQ-GITKPAIRRLARRGGVKRISGLIYEETRGVLKVFLENVIRDAVTYTEHARRKTVTAMDVVYALKRQGRTLYGFGG-  103
GS07034          H-RKVLRDNIQ-GITKPAIRRLARRGGVKRISGLIYEETRGVLKVFLENVIRDAVTYTEHARRKTVTAMDVVYALKRQGRTLYGFGG-  103
GS07988          H-RKVLRDNIQ-GITKPAIRRLARRGGVKRISGLIYEETRGVLKVFLENVIRDAVTYTEHARRKTVTAMDVVYALKRQGRTLYGFGG-  103
H4_ARATH         H-RKVLRDNIQ-GITKPAIRRLARRGGVKRISGLIYEETRGVLKIFLENVIRDAVTYTEHARRKTVTAMDVVYALKRQGRTLYGFGG-  103
H4_Crein         H-RKVLRDNIQ-GITKPAIRRLARRGGVKRISGLIYEETRTVLKTFLENVIRDSVTYTEHARRKTVTAMDVVYALKRQGRTLYGFGG-  103
H4variant_Crein  H-RKVLRESINTALTQGSIRRLARRGGVKRLSGLVYNEIRSVLRGFLEALVRDTITYTEHARRQHVKLMDVMYALKRQGRTLYGFGVF  107
H4_HUMAN         H-RKVLRDNIQ-GITKPAIRRLARRGGVKRISGLIYEETRGVLKVFLENVIRDAVTYTEHAKRKTVTAMDVVYALKRQGRTLYGFGG-  103
H4G_HUMAN        H-RKVLSDNIQ-GITKCTIRRLARHGGVKRILGLIYEETRRVFKVFLENVIWYAVTNTEHAKRKTVTAMAVVYVLKRQGRTL------   98
H4_YEAST         H-RKILRDNIQ-GITKPAIRRLARRGGVKRISGLIYEEVRAVLKSFLESVIRDSVTYTEHAKRKTVTSLDVVYALKRQGRTLYGFGG-  103
H42_TETTH        HSRKSNKASIE-GITKPAIRRLARRGGVKRISSFIYDDSRQVLKSFLENVVRDAVTYTEHARRKTVTAMDVVYALKRQGRTLYGFGG-  103
H41b_Ptric       H-RKVLRDNIQ-GITKPAIRRLARRGGVKRISGLIYEETRGVLKVFLENVIRDSVTYTEHARRKTVTAMDVVYALKRQGKTLYGFGG-  103
H4_Tbruc         Q-KKVLRENVR-GITRGSIRRLARRGGVKRISGVIYDEVRGVLKSFVEGVVRDATAYTEYSRKKTVTAVDVVNALRKRGKILYGYA--  100
                 .......160.......170.......180.......190.......200.......210.......220.......230........
```

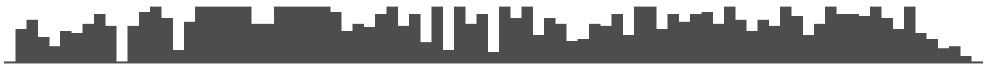

Supplementary Figure S13.

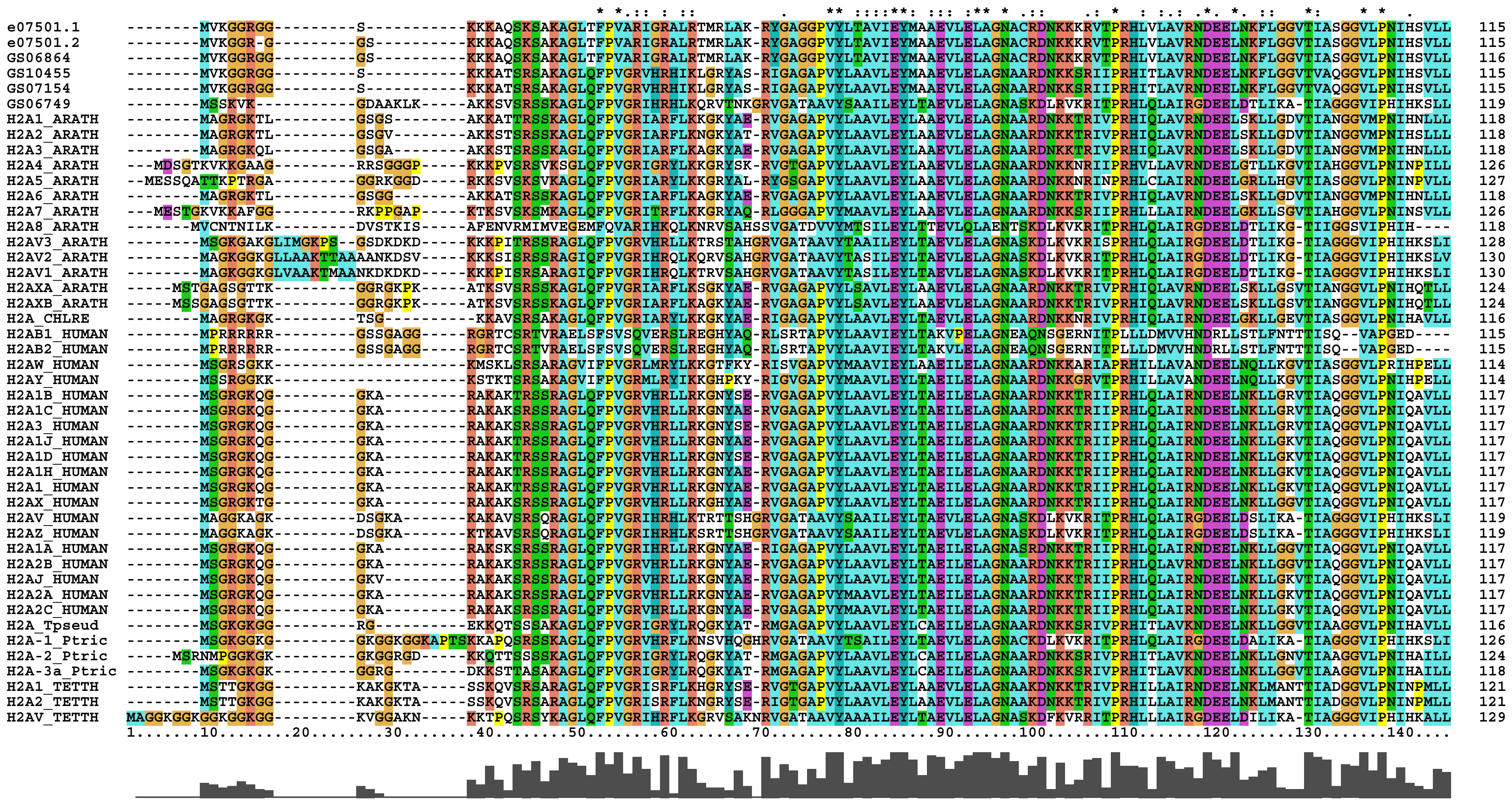

Supplementary Figure S14.

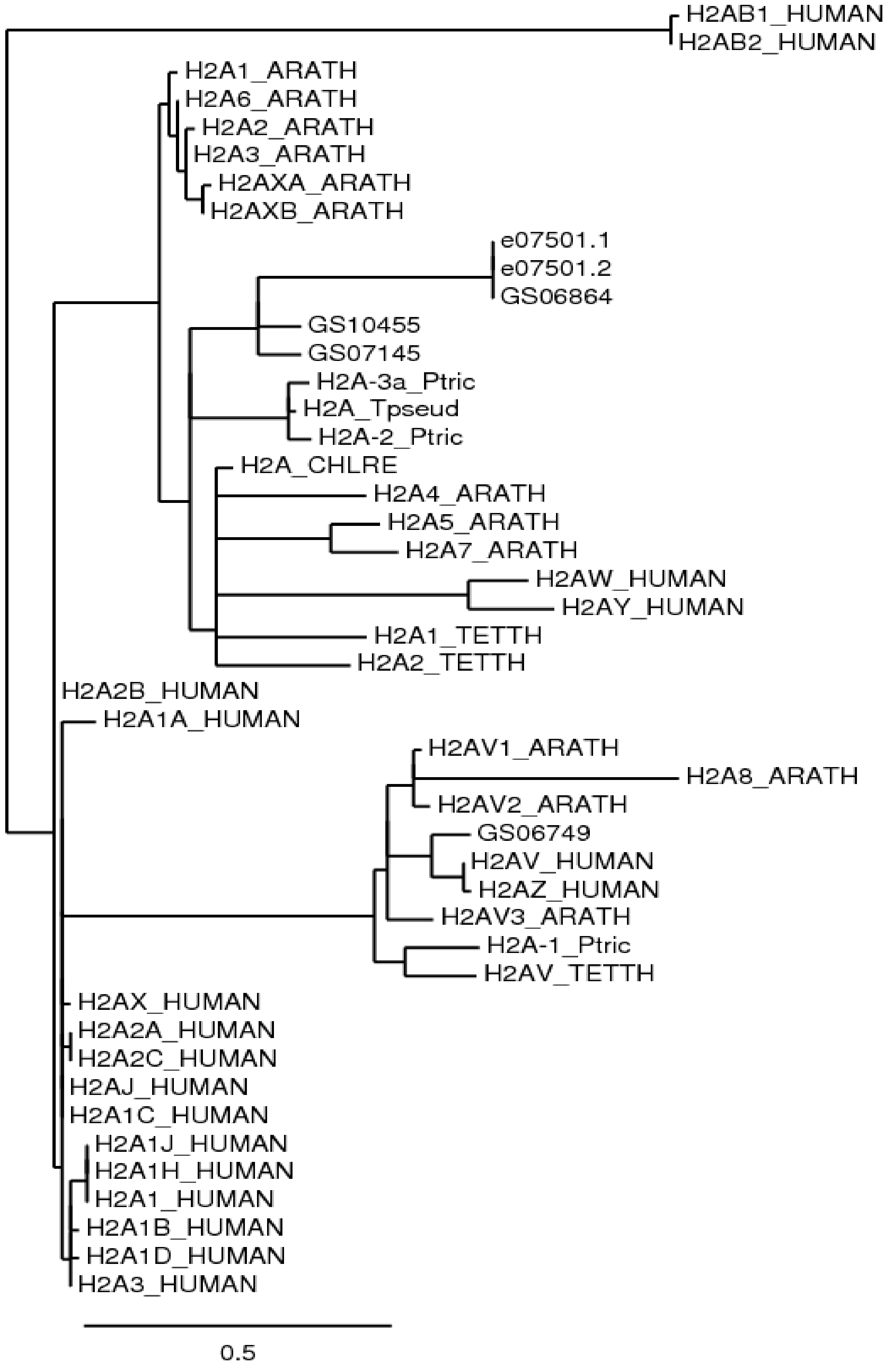

Supplementary Figure S15.
