# Supplementary material for: Transcriptome analysis of functional differentiation between haploid and diploid cells of Emiliania huxleyi, a globally significant photosynthetic calcifying cell
Source: Genome Biol. 2009 Oct 15;10(10):R114. doi: 10.1186/gb-2009-10-10-r114 (PMC2784329; doi:10.1186/gb-2009-10-10-r114)
Supplement: Additional data file 7 — Tables S1 and S2 list all oligonucleotide primers and a summary of RT-PCR results. [file gb-2009-10-10-r114-S7.doc]

**RT-PCR results.** In many cases multiple primer sets were used to interrogate each cluster. Supplementary Table S1 provides a detailed summary, including the primers used and information on products obtained for all PCR reactions performed. Supplementary Table S2 provides an abbreviated summary comparing the results of prediction by inter-library comparison (based on 1N and 2N read numbers) to the combined result of RT-PCR tests using one or more primer pairs.

**Supplementary Table S1. Primers used and summary of RT-PCR tests.** Predicted product size is based on the EST cluster or mini-cluster consensus sequence. Results are summarized with the following codes: “+”, detected; “+, m”, detected with ≥1 distinct products higher than predicted size based on cluster or mini-cluster consensus sequence (possibly resulting from multiple transcript products due to alternative splicing); “+, >”, detected with a size larger than predicted based on EST cluster or mini-cluster consensus sequence (due to introns in genomic DNA); “+, >>”, detected with size much larger than predicted based on EST cluster or mini-cluster consensus sequence (due to one or more introns in genomic DNA); “+/-“, very faint product detected; ;“-“, not detected in samples; “- *”, not detected but genomic DNA product is likely larger than amplifiable in PCR conditions used.

| **Primer name** | **Primer sequences** | **Predicted product size** | **1N cDNA** | **2N cDNA** | **1N gDNA** | **2N gDNA** |
| --- | --- | --- | --- | --- | --- | --- |
| ***Predicted to be expressed well in both 1N and 2N cells:*** | | | | | | |
| GS00217 elongation factor 1α | | | | | | |
| e00217F1 | AAGTTCAAGGTCGGCAAGG | 207 | + | + | +, > | +, > |
| e00217R1 | CTTCTGGATGACGGACACG |  |  |  |  |  |
| e00217F2 | AGGTCGGCTGGAAGAAGG | 191 | + | + | +, > | +, > |
| e00217R2 | GCAGAAGTCGTTGAGGCACT |  |  |  |  |  |
| GS09822 GPA | | | | | | |
| e09822F1 | GGAGAAGCCGAGCCTGAT | 236 | + | + | +, = | +, = |
| e09822R1 | ATGTCCCAGCCCGAAGAG |  |  |  |  |  |
| GS06749 histone H2A | | | | | | |
| e06749F1 | AAGGACTGAGCACACCAACA | 204 | + | + | +, = | +, = |
| e06749R1 | TACAGCCTCACATGGACGAC |  |  |  |  |  |
| ***Predicted to be highly 1N-specific (0 2N reads, p<0.01)*** | | | | | | |
| GS00508 1N-specific putative cyclin | | | | | | |
| e00508.1F1 | CCGAAGAGATGATCCTGGAA | 299 | +, < | -, < | +, >> | +, >> |
| e00508.1R1 | AGCGTGTACGTGCACTTGAG |  |  |  |  |  |
| e00508.1F2 | AGCCTACCTGGACCGCTAC | 290 | + | - | +, >> | +, >> |
| e00508.1R2 | TCTCTTCGGCTATCCTCTGC |  |  |  |  |  |
| GS01285 1N-specific ankaryin-repeat protein | | | | | | |
| e01285F1 | GGGCTTGACACTTTTGCTCT | 198 | + | - | +, = | +, = |
| e01285R1 | CGAACAGGAACTCAGAACCAG |  |  |  |  |  |
| GS00910 possible cGMP protein kinase | | | | | | |
| e00910F1 | GCGAAATCCTCTCCATCAAC | 181 | + | - | +, > | +, > |
| e00910R1 | CTACCCCCTACCCGCAAT |  |  |  |  |  |
| GS00234 1N-specific calcium/calmodulin-dependent protein kinase | | | | | | |
| e00234F1 | CGACCTACCACCTCTGCTTC | 199 | + | +/- | +, = | +, = |
| e00234R1 | GCGTCCTTCTCCACGATG |  |  |  |  |  |
| GS00184 1N-specific protein kinase | | | | | | |
| e00184F1 | TTGCCACGATGAGTTTATGC | 267 | + | +/- | +, > | +, > |
| e00184R1 | TAGAGGAGCGAGGTGAAGGA |  |  |  |  |  |
| GS00273 Myb superfamily transcription factor | | | | | | |
| e00273F1 | ACGGTTTCCCTGCTCTGC | 249 | + | - | +, = | +, = |
| e00273R1 | CGTCCACACCATCTTCTTGA |  |  |  |  |  |
| GS00667 outer arm DHC-beta | | | | | | |
| GS00667F1 | TGAACCTCGTCCTCAACACA | 543 | + | - | +, = | +, = |
| GS00667R1 | GAATCATCGGCATCACTGG |  |  |  |  |  |
| GS00242 conserved flagellar-related protein | | | | | | |
| e00242F1 | GCTCTGAAGCAGCAGGAGAT | 205 | + | - | +, > | +, > |
| e00242R1 | GATAGAATGGCTCCGTCGTC |  |  |  |  |  |
| GS00012 inner arm DHC2 | | | | | | |
| e00012-F1 | GCAGCGAGAAGGACTACGAG | 205 | + | - | +, = | +, = |
| e00012-R1 | CGCGAGCACACTCACATTAT |  |  |  |  |  |
| GS02894 false GPA homolog | | | | | | |
| e02894F2 | AGAGGTAGCGGTGGCAGAG | 349 | + | +/- | +, = | +, = |
| e02894R2 | GCGGGCAGAATGTTAGAGAA |  |  |  |  |  |
| GS00157 1N-specific putative beta-carbonic anhydrase | | | | | | |
| GS00157F1 | GCAAGGTCCGATTCTTCG | 243 | + | - | +, = | +, = |
| GS00157R1 | GCCGCTACATCCCTCAAAC |  |  |  |  |  |
| GS02990 1N-specific putative DNA N-6-adenine-methyltransferase | | | | | | |
| e02990F1 | GATGGGCGGTAGACTTTGAC | 258 | + | - | +, = | +, = |
| e02990R1 | ACGATTGGTGCGAGAGCA |  |  |  |  |  |
| GS01257 1N-specific orphan | | | | | | |
| e01257F2 | GACGCTGACAAGGGGATGT | 378 | + | +/- | +, = | +, = |
| e01257R2 | GACGACGCAGAGGTAGCC |  |  |  |  |  |
| GS01805 1N-specific orphan | | | | | | |
| e001805F1 | CCAAGGAGGACGAGCAGTA | 300 | + | +/- | +, = | +, = |
| e001805R1 | TGCGGTCTGGGAATCTGA |  |  |  |  |  |
| e001805F2 | ACCACTCGCACGCAACAC | 309 | + | - | +, = | +, = |
| e001805R2 | ACACAGAACGCACGGACAC |  |  |  |  |  |
| ***Predicted to be highly 2N-specific (0 1N reads, p<0.01)*** | | | | | | |
| GS00451 2N-specific putative aquaporin | | | | | | |
| e00451F1 | TGGGCACCTTTCTCCTCAT | 295 | +/- | + | - | - |
| e00451R1 | ATCATCACCAGCCAGACCAT |  |  |  |  |  |
| e00451F2 | TGTCGCTATGACGCTATTCG | 158 | +/- | + | +, = | +, = |
| e00451R2 | CAGAGGTGTGGGCAGTCG |  |  |  |  |  |
| GS02435 2N-specific histone H4 variant | | | | | | |
| e02435F2 | AGCCACACACAACCTCACAG | 228 | - | + | - | +, = |
| e02435R2 | CGTAATCGCTCCCAAGAAAG |  |  |  |  |  |
| e02435F4 | CTCTCGGTCCTCGCAATG | 574 | - | + | - | +, = |
| e02435R4 | CGTGCTCCGTGTAGGTGAC |  |  |  |  |  |
| e02435F5 | GCTGGCAACGATGTTCAGT | 192 | - | + | - | +, = |
| e02435R5 | GTGATGCCCTGGATGTTTTC |  |  |  |  |  |
| GS00463 NCKX | | | | | | |
| e00453AF1 | GAGGAGGAGGAGAAGGAGGA | 198 | - | + | +, = | +, = |
| e00453AR1 | GACCATGACGAAGGAGAGGA |  |  |  |  |  |
| e00453,2BF1 | TGCTCATCGTCAAGAACGAC | 976 | - | + | - * | - * |
| eOO453,2BR1 | CCGTAGACACAACCGCACT |  |  |  |  |  |
| GS05051 SLC4 homolog | | | | | | |
| e05051,4F1 | CGCTCTTCCAGTTCTCCTG | 191 | - | + | +, = | +, = |
| e05051,4R1 | GGCTCCCATCCCTGTTTACT |  |  |  |  |  |
| e05051,3F1 | AAGGGGAAGAAGCCCATC | 184 | - | + | +, = | +, = |
| e05051,3R1 | AGAGGCAGGCGAAGAAGAG |  |  |  |  |  |
| GS02941 t-SNARE homolog | | | | | | |
| e02941,1F1 | CGGGCAGGAGGAGGTCTT | 321 | - | +, M | +, >> | +, >> |
| e02941,1R1 | ACGACAATCAGCAGCGTCA |  |  |  |  |  |
| e02941,2F1 | TCACTGGCTCGTCTCTTCAC | 317 | - | + | +, = | +, = |
| e02941,2R1 | CGTCCATCGCCTTGAGTT |  |  |  |  |  |
| e02941,4F1 | AGGAGGTGAATGGCGTGA | 340 | - | + | +, = | +, = |
| e02941,4R1 | CGGTGCTTGGATTTGAACC |  |  |  |  |  |
| GS03351 2N-specific unknown protein | | | | | | |
| GS03351F1 | GGGCAAGACGGAGCAGTA | 390 | - | + | +, > | +, > |
| GS03351R1 | CAGGCGAGTGAAAAAGTGC |  |  |  |  |  |
| GS02507 2N-specific orphan | | | | | | |
| e02507F2 | GCGAGTCCACCAAATAGGAA | 397 | - | + | +, = | +, = |
| e02507R2 | CGCCTTGCGAACCATCTC |  |  |  |  |  |
| GS11002 orphan present in 2N library | | | | | | |
| e11002F1 | CCAAGAAGAAGTAAGCGACCTG | 218 | + | + | +, = | +, = |
| e11002R1 | CGACAGACCGAGAACCCTAT |  |  |  |  |  |
| GS01164 2N-specific orphan | | | | | | |
| GS01164F1 | CGAAGAGCCCGAGATGTA | 176 | - | + | +, = | +, = |
| GS01164R1 | CAAAGCCGTAGCAGGTCAAC |  |  |  |  |  |
| GS01802 2N-specific orphan | | | | | | |
| GS01802F1 | GAAAACCTCCCCACGATTG | 444 | - | + | +, > | +, > |
| GS01802R1 | GTCCCAGTGTTGTGCTGTTG |  |  |  |  |  |
| ***Others tested for 1N-specificity*** | | | | | | |
| GS04411 ODA-DC3 | | | | | | |
| 4602AF1 | AGTTTCTGGGGTGCAAGATG | 292 | + | - | +, > | +, > |
| 4602AR1 | TTTTCCTCCTCGAAGACAGC |  |  |  |  |  |
| GS02579 inner arm DHC1b | | | | | | |
| EH001948BF1 | GCTTTCTCACTGCGCTCAT | 293 | + | - | +, = | +, = |
| EH001948BR1 | GTAGAGCGGGCACGAGTACA |  |  |  |  |  |
| GS02889-GS03135 cytoplasmic DHC | | | | | | |
| EH001876BF1 | GTCGTCGTAAGTGGCCTTG | 296 | + | - | +, = | +, = |
| EH001876BR1 | GTGCGTTATTGCGTTCACTC |  |  |  |  |  |
| e03135F1 | GCGGACTATTTGCGTGCTAT | 215 | + | - | +, = | +, = |
| e03135R1 | TGCCACAGCGACAGGACT |  |  |  |  |  |
| e03135F2 | GTGGCTGACTACCGAGCAG | 416 | + | - | +, = | +, = |
| e03135R2 | GCAGGAAGGAATGGAGCAG |  |  |  |  |  |
| e03135F1 | GCGGACTATTTGCGTGCTAT | ≈560 | + | + | +, = | +, = |
| e02889F3R | GAGAGAGCAAGCCACCACC | (measured) |  |  |  |  |
| GS02724 FAP58/FAp189 conserved flagellar protein | | | | | | |
| 1926AF1 | ATTGCGGAGAAGGACAAGC | 426 | + | - | +, = | +, = |
| 1926AR1 | GCACACAGGACTCGTAGCAT |  |  |  |  |  |
| GS00844 conserved basal body protein BBS5 | | | | | | |
| e00844F1 | TTGCCACAGACGACGAGA | 210 | +, m | - | +, > | +, > |
| e00844R1 | TCAAGAAGAAGGTGCCCAAG |  |  |  |  |  |
| e00844F2 | TTTTGCCACAGACGACGAG | 271 | +, m | - | +, > | +, > |
| e00844R2 | TGGGATGGAGACATTGAAGC |  |  |  |  |  |
| e00844F3 | GAGTCAAAGGCTGCTTCTCC | 485 | +, m | - | +, > | +, > |
| e00844R3 | CCCTCTCCTCGCTGTCATC |  |  |  |  |  |
| GS00132 phototropin homolog | | | | | | |
| e00132F1 | TCACCCTGCTCAACTACAAGG | 241 | + | - | +, = | +, = |
| e00132R1 | CTCACGACACCCGACATTC |  |  |  |  |  |
| e00132F2 | TTCCCGACCTTCATTTTCTC | 401 | + | - | +, = | +, = |
| e00132R2 | CTCGCCTTGTAGTTGAGCAG |  |  |  |  |  |
| GS00920 phototropin homolog | | | | | | |
| e00920F1 | CGAAGTCTCCAGCCAAGC | 202 | + | - | +, = | +, = |
| e00920R1 | GCCAACCTGAGCAACCTT |  |  |  |  |  |
| e00920F2 | AACCCGATGTGCTTTGTCA | 369 | + | - | +, = | +, = |
| e00920R2 | GGCTTGGCTGGAGACTTC |  |  |  |  |  |
| GS05223 false agglutinin homolog | | | | | | |
| e05223F1 | GACAGTCCACACACCGACAG | 203 | + | - | +, = | +, = |
| e05223R1 | CCTCGCATTGAAGACTTGGT |  |  |  |  |  |
| e05223F2 | CAGGTTGGAGAGGAAAAGGA | 202 | + | - | +, = | +, = |
| e05223R2 | CGAAAGGGGTAGCGGTTAGT |  |  |  |  |  |
| e05223F2 | See above | 242 | + | - | +, = | +, = |
| e05223R1 | See above |  |  |  |  |  |
| GS10455 1N-specific histone H2A variant | | | | | | |
| e10455F1 | AACAAGAAGTCGCGCATCA | 187 | + | - | +, = | +, = |
| e10455R1 | GAAACACCTGTGGGGAGAGA |  |  |  |  |  |
| e10455F2 | GCCGTCCTTGAGTACATGG | 299 | + | - | +, = | +, = |
| e10455R2 | GAGAGCGGAAGAACACATCC |  |  |  |  |  |
| ***Others tested for 2N-specificity*** | | | | | | |
| GS00304 VCX1 | | | | | | |
| e00304F1 | TCACCAAGCAGCAAAAGTTC | 185 | - | + | +, = | +, = |
| e00304R1 | GCAGACATAGACGACGAGCA |  |  |  |  |  |
| e00304F2 | TGCTCGTCGTCTATGTCTGC | 629 | - | + | +, > | +, > |
| e00304R2 | CTCCTACTCGTGATGGTGCTC |  |  |  |  |  |
| GS03082 predicted biomineralization transcript (Quinn et al. 2006) | | | | | | |
| e03082.1+2F1 | CAGTTCGTCGCCAAGTTTTC | 433 | + | + | +, > | +, > |
| e03082.1+2R1 | TGTAGGTGTCTCCCCAGCA |  |  |  |  |  |

**Supplementary Table S2.** Abbreviated summary table comparing EST read numbers from each library with summarized RT-PCR results for each cluster.

| Cluster | 1N EST # | 2N EST # | RT-PCR  1N | RT-PCR 2N |
| --- | --- | --- | --- | --- |
| Predicted to be expressed in both phases |  |  |  |  |
| GS00217 | 4 | 7 | + | + |
| GS09822 | 1 | 1 | + | + |
| GS06749 | 4 | 3 | + | + |
| Predicted to be highly 1N-specific |  |  |  |  |
| GS00508 | 6 | 0 | + | - |
| GS01285 | 6 | 0 | + | - |
| GS00910 | 14 | 0 | + | - |
| GS00234 | 7 | 0 | + | +/- |
| GS00184 | 6 | 0 | + | +/- |
| GS00273 | 8 | 0 | + | - |
| GS00667 | 7 | 0 | + | - |
| GS00242 | 8 | 0 | + | - |
| GS00012 | 9 | 0 | + | - |
| GS02894 | 6 | 0 | + | +/- |
| GS00157 | 12 | 0 | + | - |
| GS02990 | 15 | 0 | + | - |
| GS01257 | 25 | 0 | + | +/- |
| GS01805 | 16 | 0 | + | +/- |
| Predicted to be highly 2N-specific |  |  |  |  |
| GS00451 | 0 | 7 | +/- | + |
| GS02435 | 0 | 6 | - | + |
| GS00463 | 0 | 8 | - | + |
| GS05051 | 0 | 7 | - | + |
| GS02941 | 0 | 9 | - | + |
| GS03351 | 0 | 14 | - | + |
| GS11002 | 0 | 16 | + | + |
| GS02507 | 0 | 12 | - | + |
| GS01164 | 0 | 10 | - | + |
| GS01802 | 0 | 10 | - | + |
| Others tested for 1N-specificity |  |  |  |  |
| GS04411 | 2 | 0 | + | - |
| GS02579 | 4 | 0 | + | - |
| GS03135 | 2 | 0 | + | - |
| GS02889 | 5 | 0 | + | - |
| GS02724 | 5 | 0 | + | - |
| GS00920 | 4 | 0 | + | - |
| GS00132 | 3 | 0 | + | - |
| GS00844 | 2 | 0 | + | - |
| GS05223 | 3 | 0 | + | - |
| GS10455 | 2 | 0 | + | - |
| Others tested for 2N-specificity |  |  |  |  |
| GS00304 | 0 | 4 | - | + |
| GS03082 | 0 | 2 | + | + |
