# Supplementary material for: Transcriptome analysis of functional differentiation between haploid and diploid cells of Emiliania huxleyi, a globally significant photosynthetic calcifying cell
Source: Genome Biol. 2009 Oct 15;10(10):R114. doi: 10.1186/gb-2009-10-10-r114 (PMC2784329; doi:10.1186/gb-2009-10-10-r114)
Supplement: Additional data file 8 — Detailed description of identification and analysis of flagellar-related homologs. [file gb-2009-10-10-r114-S8.doc]

**Identification of *E. huxleyi* clusters homologous to a conservative set of known protein components of the eukaryotic flagellum/cilium and basal bodies**

There have been several comparative genomics and large scale expression analyses to identify conserved protein components of the eukaryotic flagella/cilia or genes/proteins involved in its construction [26-28,30,31]. However, we chose to base our analysis on Pazour et al. (2005) Table S3 because this provides the most recent summary of genes and proteins identified by classical biochemical and genetic techniques and thus should be more conservative. We collected all publicly available sequences identifiable from Pazour et al. (2005) Table S3 [3] in Genebank or version 2 of the *C. reinhardtii* genome assembly [84]. Five of the sequences could not be identified in public sequence databases (IA1-IC97, RSP15, IFT27, IFT46, and IFT144). We updated the sequences with the latest version of the *C. reinhardtii* genome assembly [84] resulting in a total of 100 proteins. When predicted protein sequences showed minor difference between the *C. reinhardtii* gene catalog and the nr database for ostensibly the same protein, both sequences were used as query. These sequences were then used to query our EST database and clusters with E-value homology scores better than 1x10^-4^ were considered “hits”. These “hits” were then further classified by blast search against the version 3 *C. reinhardtii* assembly protein catalog. “Best hits” were classified as clusters that had better homology scores to the *C. reinhardtii* flagellar-associated protein set than to any *C. reinhardtii* protein model not in the flagellar-associated set. In most cases, “hits” that were not also “best hits” were clusters with higher homology to proteins not specifically associated with the flagella but that are distant paralogs of flagellar-associated proteins. In certain cases, clusters classified as “hits” that were not “best hits” had higher homology to *C. reinhardtii* paralogs of the query sequence that have been identified as also being flagellar-associated since 2005. Such clusters were also considered to represent likely flagellar-related transcripts. The query sequence identifiers and query results are given in the excel file Supp8.xls in worksheet “query results” and the summary after supplementation by manual inspection is in the worksheet “Ehux flagellar-related clusters”.

We could identify clear homologues for 64 out of 100 total *C. reinhardtii* flagellar-associated proteins, covered by a 132 total EST clusters (i.e., an average of two *E. huxleyi* EST clusters per homolog). Many flagellar-related proteins are close paralogs of each other and this likely causes the figure 64/100 to be an underestimate of the true proportion of *C. reinhardtii* flagellar-associated proteins with homologs in the *E. huxleyi* database; often more than one distinct cluster would match best to a *C. reinhardtii* gene while close *C. reinhardtii* paralogs received no best matches in the *E. huxleyi* EST databases. In such cases, further sequence length and phylogenetic analysis might reveal that both *C. reinhardtii* paralogs were represented in the *E. huxleyi* ESTs. The list of likely flagellar-associated clusters was manually supplemented by searching for clusters with top Uniprot or Swissprot hits with key words “flagella”, “dynein”, “basal body” or “Bardet-Biedl”. Bardet-Biedl syndrome proteins have recently been identified to be a non-homologous group of proteins involved in basal body formation [29, 31]. Including these manually identified clusters raised the number of total homologs of flagellar-related proteins to 153 (see Supp8.xls, worksheet “Ehux flagellar-related clusters”).

17 were clusters homologous to dynein heavy chains (DHCs), highly conserved motor proteins (≈4000 amino acid residues) that are well characterized in both *C. reinhardtii* and animal flagella and cilia [55, 56]. The DHC-related clusters were composed of a total of 64 ESTs exclusively from the 1N library. We identified homologs of outer arm, inner arm, and cytoplasmic DHCs among the *E. huxleyi* EST clusters. 1N-specific expression was confirmed by RT-PCR for two clusters displaying homology to inner arm DHCs (GS02579 and GS00012, represented by 4 and 9 1N-ESTs, respectively). Two clusters displayed homology to cytoplasmic DHC: GS03135 was represented by 2 ESTs from the 1N library and 0 from the 2N library and had a top BLAST hit in the *C. reinhardtii* database to cytoplasmic DHC1b (E-value 8x10^-62)^ and in Swissprot to *Tripneustes gratilla* (sea urchin) cytoplasmic DHC2 (DYHC2_TRIGR, E-value 1x10^-61^). GS02889 was represented by 5 reads in the 1N library and 0 in the 2N library (*p=*0.01565) and also had a top BLAST hit in the *C. reinhardtii* database to cytoplasmic DHC1b (E-value 2x10^-30^), likely involved in intraflagellar transport, and also in Swissprot to DYHC2_TRIGR (E-value 3x10^-30^). Both GS03135 and GS02889 were detected only in RNA from 1N cells and not from RNA from 2N cells, suggesting they might be associated with flagellar transport in *E. huxleyi*.

We investigated how many of the distinct DHC-related EST clusters might in fact represent distinct genes. Normally, the EST clusters should map to the 3’ end of genes. Rare prematurely terminated ESTs will form distinct mini-clusters but be grouped into the same cluster as 3’-mapping ESTs during our bioinformatics pipeline. However, the DHC genes are so large that we suspected ESTs representing transcripts originating from these genes would be prone to clustering failure, i.e., where ESTs originating from the same locus were not assigned to the same final cluster (either due to the size of the DHC loci themselves or due to gaps in the whole genome assembly). We examined BLASTX alignments of DHC-related clusters to top hits to *C. reinhardtii* proteins in the nr database (see analysis at end of this document). GS06462 and GS00667 both had top hits to the same outer arm DHCα (ODA11) protein (XP_001695733) (E-values 3x10^-52^ and 7x10^-14^, respectively), yet GS00667 matched the N-terminus (residues 4231-4497) whereas GS06462 matched in the middle of the same DHC1a (1319-1506. GS06462 may represent a prematurely terminated transcript originating from the same gene as GS00667. GS00095 and GS01613 both mapped by BLASTX to the N-terminus of outer arm DHCβ (ODA4) (XP_001695126). The three mini-clusters composing GS01613 mapped to residues 3955-4547 while GS00095 mapped to residues 4252-4499, but with distinct predicted amino acid sequences (nucleotide sequences of GS00095 also did not align with GS01613). In contrast, GS03181 also had a best BLASTX hit to XP_001695126, but to residues 2910-3055. It is likely that GS03181 is originates from the same gene as either GS00095 or GS01613, but GS00095 and GS01613 appear to be distinct. A similar analysis revealed that GS00730 might be a version of the same gene as GS02579, a top homolog of the *C. reinhardtii* inner arm DHCb (DHC1b/IDA2). Finally, GS02889 mapped to amino acid residues 4138-4332 of *C. reinhardtii* cytoplasmic DHC1b but GS03135 mapped to residues 3805-4063. PCR successfully amplified a product of ≈560 nt from both cDNA and genomic DNA using a forward primer designed to the 3’ end of GS03135 and a reverse primer to the 5’ end of GS02889, confirming that these two clusters arise from the same gene. This analysis suggests that the 17 DHC-related clusters may represent 13 distinct DHC genes.

We also chose two other clusters related to central structural components of the eukaryotic flagellum for RT-PCR assay. Clusters GS02246 and GS04411 were both homologous to the *C. reinhardtii* outer dynein arm docking complex protein ODA-DC3 (ODA14) (E-values 2x10^-26^ and 2E-22). Cluster GS02246 was represented by 5 1N-ESTs and no EST from the 2N library (*p=*0.01565). Cluster GS04411 was detected by 2 ESTs from the 1N library and 0 from the 2N. Therefore GS04411 was selected for testing by RT-PCR, which confirmed that expression of GS04411 was detectable only from 1N cells. Three clusters showed strong homology to FAP189 and to FAP58/MBO2, highly conserved but poorly characterized coiled-coil proteins identified in the *C. reinhardti* flagellar proteome. In total they were represented by 12 ESTs from the 1N library and 0 ESTs from the 2N library. GS02724, represented by 5 ESTs from 1N (*p=*0.01565), had the highest homology to *C. reinhardtii* FAP189 (E-value=8x10^-68^) and was confirmed by RT-PCR to be detectable only from 1N cells.

Eight clusters had homology to basal body components, many of which have begun to be studied for their involvement in human diseases, e.g., Bardet-Biedl Syndrome (BBS) proteins. Homologs of three BBS proteins could not be identified (BBS4, BBS6, BBS8). Cluster GS00844 had high homology to the well conserved BBS5 protein (top Swissprot hit to *Danio rerio* BBS5, E-value 3x10^-91^). It was represented by only 2 EST reads from 1N and none from 2N. RT-PCR confirmed that it was indeed expressed only in 1N cells. Curiously, three non-overlapping primer sets designed to GS000844 all detected evidence of incompletely spliced transcript products.

The following flagellar-associated proteins were considered as having well-known roles in the cytoplasmic body, independent of other flagellar roles:

Tubulin [85], actin [85], caltractin/centrin [93, kinesin-like protein [92], phosphatase 1 [87], microtubule-associated protein, glycogen synthase kinase 3 [89], calmodulin [88], HSP70 [91], phototropin [86], protein phosphatase 2a [90]. In addition, Cluster GS09611, classed as deflagellation inducible protein, 13KD (DIP13) is considered a cytoplasmic protein because it had a slightly higher homology to the human protein Sjoegren syndrome nuclear autoantigen 1 (SSNA1) (6x10^-27^) than to the *C. reinhardtii* flagellar associated protein. Cluster GS01953, originally identified as related to *C. reinhardtii* flagellar-associated protein A8JDM7 (E-value 3x10^-44^) was classed by KOG as a homolog of the mitochondrial protein prohibitin, so is considered likely to have a role outside of the flagella. Cluster GS00260 had high homology *C. reinhardtii* flagellar associated protein (A8JC09_CHLRE) (E-value 5x10^-96^) but also high homology to the *S. cerevisiea* protein ARB1_YEAST (E-value 5x10^-78^), an ABC transporter. Thus it is likely to represent a protein with cytoplasmic roles.

There were 93 clusters that appeared to be homologs of flagellar-related proteins with no obvious cytoplasmic role outside the flagella. These were represented by 281 reads from the 1N library and only 9 reads from the 2N library.

The manual inspection of the list identified a few cases of likely false positive identification of clusters as potentially homologous to flagellar-related proteins, as flagged in the tables:

1. GS01656 is “best hit” to *C. reinhardtii* flagellar dynein heavy chain 9 but it has a relatively low score (1x10^-18^) and it’s top Uniprot/Swissprot hits are to uracil phosphoribosyltransferases (4x10^-46^ and 5x10^-46^) in bacteria.
2. Radial spoke protein 2. GS03282 could be a false positive identification. Blast of *C. reinhardtii* protein against nr database retrieves only the query protein. E-score with *E. huxleyi* EST data looks satisfactory (1x10^-7^). It may represent a protein only found in *C. reinhardtii* and *E. huxleyi*. However, the *C. reinhardtii* protein is very repeat rich.
3. Radial spoke protein 10. Like many radial spoke proteins, it contains the common MORN repeat domain but this domain is also found in many proteins not related to flagella. Pfam describes the MORN domain as originally identified in *Toxoplasma* and thought to be involved in cytoskeletal-membrane linkages. The GS07344 and GS02465 may be false positive identification because the homology is based on the MORN domain. GS02465 received high hits to other MORN repeat proteins including from bacteria (ref|ZP_00956819.1| MORN repeat protein [Sulfitobacter sp. EE-36], 3x10^-23^), so likely a false positive.
4. Radial spole protein 16. GS12118 is “best hit” to RSP16 but may be a false positive identification because best Uniprot and Swissprot hits are to DNAJ/HSP40 in other organisms (e.g. ref|NP_001027731.1| UniGene infoGene info heat shock protein 40 [Ciona intestinalis], 7x10^-22^, and emb|CAO50141.1| unnamed protein product [Vitis vinifera]), 3x10^-21^) , better hits than to *C. reinhardtii* RSP16 (1x10^-20^).
5. Radial spoke protein 23. GS02958 is “best hit” to RSP23 (1x10^-29^) but other high Uniprot/Swissprot hits are not to flagellar-specific proteins, such as animal nucleotide diphosphate kinase 7 (GENE ID: 100196066 ndk7 | Nucleoside diphosphate kinase 7 [Salmo salar], 1x10^-25^).
6. Intraflagellar transport particle protein IFT140. GS04384 is “best hit” to intraflagellar transport particle protein IFT140, but with a much lower score (3x10^-10^) than GS07726, the other “best hit” cluster to same *C. reinhardtii* protein (with a score of 3x10^-47^) so GS04384 could be false positive
7. GS02023 was identified as a “best hit” to one version of the *C. reinhardtii* phototropin sequence (gi|20797097|emb|CAC94941.1| putative blue light receptor (phototropin), 1x10^-12^). This degree of homology was low and not substantially higher than the homology to a non-phototropin homolog from *Mus musculus* (KS6A4_MOUSE, Ribosomal protein S6 kinase alpha-4 (EC 2.7.11.1) (Nuclear mitogen-and stress-activated protein kinase 2) (90 kDa ribosomal protein S6 kinase 4) (RSK-like protein kinase) (RLSK), 3x10^-11^). Therefore it is considered not to be a true phototropin homolog.

Removing likely “false positive” homologs of flagellar-related proteins and homologs of proteins with known non-flagellar roles (described above) left 82 clusters expected to represent highly flagellar-specific genes. Consistent with this prediction, the highly flagellar-specific clusters were represented by 252 reads from the 1N library and 0 reads from the 2N library. See Supplementary File S8, worksheet “Ehux flagellar-related clusters”.

Other notes:

1. MBO2, FAP58 and FAP189: GS02724 and GS01417 were originally identified as “hits” but not “best hits” of *C. reinhardtii* protein FAP58. However they are both much closer in homology (E-values 8x10^-68^ and 3x10^-63^) to the *C. reinhardtii* protein FAP189 (>gi|159471389|ref|XP_001693839.1| flagellar associated protein [Chlamydomonas reinhardtii]) (A8HUA7_CHLRE) which was not in the original query table generated from Pazour et al. Table S3. Cluster GS05052 is composed of three separate mini-clusters. e05052.1 and e05052.3 could be combined into a single larger min-cluster. Blastx showed that this cluster had nearly identical high homology to FAP189 (1x10^-56^) as to FAP58 (3x10^-55^). Cluster GS05764, represented by a single 1N read, originally appeared as a weak hit (E-value 7x10^-6^) to MBO2. However, it had similar weak homologies to other *C. reinhardtii* proteins in the nr protein database, including a predicted basal body protein XP_001702637 (7x10^-5^). Yet, when this cluster was searched against the current version 3 of the *C. reinhardtii* genome assembly, no matches were found either by blastx against the catalog of predicted proteins or using tblastx (nucleotide vs. translated nucleotide) against the masked genome assembly. Therefore it was removed. Thus, no clear homolog of MBO2 was identified yet three clusters were homologous to two apparently related proteins, FAP58 and FAP189.

BLASTX re-analysis of DHC-related clusters against *C. reinhardtii* proteins in the nr database.

Blastx GS00667 against *C. reinhardtii*

>ref|XP_001695733.1| UniGene infoGene info flagellar outer dynein arm heavy chain alpha [Chlamydomonas reinhardtii]

gb|EDP01520.1| Gene info flagellar outer dynein arm heavy chain alpha [Chlamydomonas reinhardtii]

Length=4500

GENE ID: 5721278 ODA11 | flagellar outer dynein arm heavy chain alpha

[Chlamydomonas reinhardtii] (Over 10 PubMed links)

Score = 201 bits (512), Expect = 3e-52

Identities = 115/276 (41%), Positives = 151/276 (54%), Gaps = 13/276 (4%)

Frame = +1

Query 103 EELLREEFNMVEIKLRIKNKGDPYLVFLLQELERMNLVLNTMKSQLTELSLGLSGALNIS 282

+E L E FNMVE++LR+K K P++V LQE RMN +L+ MK + EL LGL GALN+S

Sbjct 4231 KERLPEPFNMVEVELRVKEK-TPFVVVALQEATRMNALLSEMKRSMEELQLGLDGALNMS 4289

Query 283 DAMDALINSLYLNQVPPAWLKICGQIGPTGTYNRKTLSAWYDDLQLRWKQLEDWSAPTKP 462

D M+ L + N VP W+ T TL+AWY D+ R QL W+A

Sbjct 4290 DNMEKLAKGIASNTVPELWMSCMS----TRVQEVYTLTAWYQDVVKRHDQLSAWTAGDII 4345

Query 463 LEECPPSVWISGTFNPMGYVTACMQVTARKEGYSLDEMRVQAEVTDVLDISTVEVQPERG 642

P SVW+ G FNP ++TA MQ AR LD M+ EVT + V G

Sbjct 4346 T---PHSVWLPGLFNPKAFLTAVMQTFARANKLPLDVMKFMTEVTRMTSPEQVTEAAPLG 4402

Query 643 TNIHGLFMEGARWDIASNCIAESFPKELHPVMPMIHVIAVTVDQLKTEGVYQCPIF---- 810

+HGL +EGARWD C+ +S P ELHP MP++ V VT DQ EG Y+CP++

Sbjct 4403 VYVHGLVLEGARWDREDGCLRDSKPNELHPAMPVLQVKPVTADQFNLEGYYECPVYTNMQ 4462

Query 811 MTTIHGPTFVFAAPLRTTVPAHKWILAGVAMVMQPD 918

++ P V LRT KW+LA VA+++Q D

Sbjct 4463 RANVYSPV-VSTFTLRTQDMPAKWVLASVALLLQDD 4497

Blastx GS06462 against *C. reinhardtii*

>ref|XP_001695733.1| UniGene infoGene info flagellar outer dynein arm heavy chain alpha [Chlamydomonas reinhardtii]

gb|EDP01520.1| Gene info flagellar outer dynein arm heavy chain alpha [Chlamydomonas reinhardtii]

Length=4500

GENE ID: 5721278 ODA11 | flagellar outer dynein arm heavy chain alpha

[Chlamydomonas reinhardtii] (Over 10 PubMed links)

Score = 73.2 bits (178), Expect = 7e-14

Identities = 58/194 (29%), Positives = 92/194 (47%), Gaps = 6/194 (3%)

Frame = +3

Query 48 FEALDEHEaalqavvpaasaDSRGVAFFSGALARWQAQLAEVRSTLEEWLEAQRLWLSLH 227

FEAL++++ +Q ++ +R +A F + WQ +L +V + E QR W L

Sbjct 1319 FEALEDNQVQVQGMIA-----NRYMATFKDEILGWQKKLNDVADVNQIMAEIQRTWAYLE 1373

Query 228 PLYSRDERLRDELPDETAEFDACDNVMRRVLGDAEASPSALKACMVEGRWDELRRVRVAL 407

L+ E ++ ELP T F A D +++VL + + + + C EG + L L

Sbjct 1374 SLFIHSEEVKKELPQATERFAAIDTEVKKVLREFQQLKNCVSCCNREGLYANLETQEREL 1433

Query 408 EGCAAALSDHlsrsrrlcprlsmlsNTQLLGVLSCGPTPREVDRHINALTSSVAAIEWLD 587

E C AL+D++ RR PR +S+ LL +LS G P V H+N ++ + LD

Sbjct 1434 EICKKALNDYMESKRRAFPRFYFVSSADLLDILSNGNNPMRVQIHMNKCFQAIDKLR-LD 1492

Query 588 VLEAESGSEPSLDG 629

E G P G

Sbjct 1493 SEEVVPGRRPKALG 1506

GS06462 might be a prematurely terminated mRNA originating from the same gene as GS00667

Blastx GS01613.1 against *C. reinhardtii*

e01613.1

>ref|XP_001695126.1| UniGene infoGene info flagellar outer dynein arm heavy chain beta [Chlamydomonas reinhardtii]

gb|EDP01834.1| Gene info flagellar outer dynein arm heavy chain beta [Chlamydomonas reinhardtii]

Length=4568

GENE ID: 5720753 ODA4 | flagellar outer dynein arm heavy chain beta

[Chlamydomonas reinhardtii] (10 or fewer PubMed links)

Score = 303 bits (776), Expect = 6e-83

Identities = 155/309 (50%), Positives = 222/309 (71%), Gaps = 3/309 (0%)

Frame = +1

Query 1 KEYLEYVEENIERETPAAYGLHPNSEINFMTRQAEDLFSAISELQPRggggeggmtmgeR 180

++ LE+++E + ETP A+GLHPN+EI F R+AE +++ +LQPR GEGGM+ ER

Sbjct 4239 RQVLEFIDEVMPPETPLAFGLHPNAEIGFKLREAESFCNSLVQLQPRESSGEGGMSAEER 4298

Query 181 VKQQLDEILEKLPELFLMIEIEERV---EERTPYVAFFLQECERMNLLVFEMGRSLRELD 351

K LDE+++KLP++F M ++ ++ + P+V +QE ERMN+L+ EM RSL ELD

Sbjct 4299 AKLVLDEVVDKLPDIFDMEDVRSKINPDDPNMPFVMVAIQESERMNMLLAEMKRSLLELD 4358

Query 352 AGLRGDLSISQPMEDLMNALFENRVPSTWEALAWPSLAPLSPWLADFLDRQRQLVEWTAD 531

GL+GDL++++PME L+ AL + VP +W LA+PSL PL WL + L R QLV+WTA+

Sbjct 4359 LGLKGDLTMTEPMERLLKALATDAVPGSWRNLAYPSLRPLGSWLGNLLARHAQLVDWTAE 4418

Query 532 LNTPKVTWISGLFNPQAFLTAVMQVTARKNEWPLDRvvttvdvtKRGPEEIEGAVREGAY 711

L+TPK W+SGLFNPQ+FLTAVMQ TAR+N+WPLD+ V +VTK+ P++IE R+GA+

Sbjct 4419 LSTPKAVWLSGLFNPQSFLTAVMQATARRNDWPLDKTVIITEVTKKQPDQIEANSRDGAF 4478

Query 712 IYGMYMDGARWDVNSGVIEDANMKELYPKLPVMLVKAVPVEKADSRDQYACPVYKTQFRG 891

I+G+ ++GARWD G ++D+ KEL+ +PV+LV+AV +KA+ +D Y CPVY T+ R

Sbjct 4479 IHGLTLEGARWDDKIGALDDSKPKELFCPMPVILVRAVTQDKAEMKDVYKCPVYTTEARF 4538

Query 892 PTYVFTAGL 918

VF A L

Sbjct 4539 REEVFEAQL 4547

e01613.2

>ref|XP_001695126.1| UniGene infoGene info flagellar outer dynein arm heavy chain beta [Chlamydomonas reinhardtii]

gb|EDP01834.1| Gene info flagellar outer dynein arm heavy chain beta [Chlamydomonas reinhardtii]

Length=4568

GENE ID: 5720753 ODA4 | flagellar outer dynein arm heavy chain beta

[Chlamydomonas reinhardtii] (10 or fewer PubMed links)

Score = 218 bits (554), Expect = 2e-57

Identities = 115/247 (46%), Positives = 160/247 (64%), Gaps = 30/247 (12%)

Frame = +2

Query 2 ERVKQQLDEILEKLPELFLMIEIEERV---EERTPYVAFFLQECERMNLLVFEMGRSLRE 172

ER K LDE+++KLP++F M ++ ++ + P+V +QE ERMN+L+ EM RSL E

Sbjct 4297 ERAKLVLDEVVDKLPDIFDMEDVRSKINPDDPNMPFVMVAIQESERMNMLLAEMKRSLLE 4356

Query 173 LDAGLRGDLSISQPMEDLMNALFENRVPSTWEALAWPSLAPLSPWLADFLDRQRQLVEWT 352

LD GL+GDL++++PME L+ AL + VP +W LA+PSL PL WL + L R QLV+WT

Sbjct 4357 LDLGLKGDLTMTEPMERLLKALATDAVPGSWRNLAYPSLRPLGSWLGNLLARHAQLVDWT 4416

Query 353 ADLNTPKVTWISGLFNPQAFLTAVMQVTARKNEWPLDRvvttvdvtKRGPEEIE------ 514

A+L+TPK W+SGLFNPQ+FLTAVMQ TAR+N+WPLD+ V +VTK+ P++IE

Sbjct 4417 AELSTPKAVWLSGLFNPQSFLTAVMQATARRNDWPLDKTVIITEVTKKQPDQIEANSRDG 4476

Query 515 ---------------------DANMKELYPKLPVMLVKAVPVEKADSRDQYACPVYKTQF 631

D+ KEL+ +PV+LV+AV +KA+ +D Y CPVY T+

Sbjct 4477 AFIHGLTLEGARWDDKIGALDDSKPKELFCPMPVILVRAVTQDKAEMKDVYKCPVYTTEA 4536

Query 632 RGPTYVF 652

R VF

Sbjct 4537 RFREEVF 4543

e01613.3

>ref|XP_001695126.1| UniGene infoGene info flagellar outer dynein arm heavy chain beta [Chlamydomonas reinhardtii]

gb|EDP01834.1| Gene info flagellar outer dynein arm heavy chain beta [Chlamydomonas reinhardtii]

Length=4568

GENE ID: 5720753 ODA4 | flagellar outer dynein arm heavy chain beta

[Chlamydomonas reinhardtii] (10 or fewer PubMed links)

Score = 200 bits (509), Expect = 2e-52

Identities = 99/184 (53%), Positives = 131/184 (71%), Gaps = 5/184 (2%)

Frame = +1

Query 4 FDLLLSFEDSGPAVPIFFLLSPGVDPAADVRALGKTMGKTEDEGKFQGVSLGQGQEPVAE 183

+DL S++D+ P PIF LSPGVD A V ALGK +G T D GK+ VSLGQGQEP+A

Sbjct 3955 YDLERSYQDASPGTPIFVFLSPGVDVAGSVEALGKKLGFTLDNGKYASVSLGQGQEPIAM 4014

Query 184 KALDQGYVEGGWVMLENIELVANWLP-KLEKKLASLEEGAHPEFRVFLTAMP----QKVV 348

L + GGWV+L+NI L +W +L+KK+ L EGAHP+FR+FL+A P ++ +

Sbjct 4015 DRLSAAHKNGGWVLLQNIHLTIDWTTNQLDKKVDKLVEGAHPDFRLFLSAEPPPSLERGL 4074

Query 349 PVPILQKSIKLTNEPPSGLKANLLRAFKNFDATIWESSSKQGELKAIIFSLCFFHSVVRE 528

P+ +LQ SIKLTNEPP GLKANL RA+ NF+ I ES +KQ E +AI+F+LC+FH+ + E

Sbjct 4075 PISLLQNSIKLTNEPPEGLKANLRRAWNNFNEEILESCAKQAEFRAIVFALCYFHAALLE 4134

Query 529 RRKF 540

R+KF

Sbjct 4135 RKKF 4138

Blastx GS00095 against *C. reinhardtii*

>ref|XP_001695126.1| UniGene infoGene info flagellar outer dynein arm heavy chain beta [Chlamydomonas reinhardtii]

gb|EDP01834.1| Gene info flagellar outer dynein arm heavy chain beta [Chlamydomonas reinhardtii]

Length=4568

GENE ID: 5720753 ODA4 | flagellar outer dynein arm heavy chain beta

[Chlamydomonas reinhardtii] (10 or fewer PubMed links)

Score = 140 bits (353), Expect = 1e-33

Identities = 92/278 (33%), Positives = 134/278 (48%), Gaps = 30/278 (10%)

Frame = +3

Query 81 DIPSDLGLLPRAQSALEESRADELLRFGAAVMSTSRVGLGPFGRSGRRSCSGTAAAETVE 260

+ P GL P A+ + A+ S V L P R SG E

Sbjct 4252 ETPLAFGLHPNAEIGFKLREAESFCN--------SLVQLQP------RESSGEGGMSAEE 4297

Query 261 AVRLVLDKVRDALPHATTRPKALQRRLRTDLSPLEADLSPLEADLSPLEVVLMQECDRME 440

+LVLD+V D LP D+ + + ++P + ++ P +V +QE +RM

Sbjct 4298 RAKLVLDEVVDKLPDIF------------DMEDVRSKINPDDPNM-PFVMVAIQESERMN 4344

Query 441 ALVTEVHASLARLDESMRGGAPVDEGADTLVGALQRGLVPASWTELAWPSAGPQPLGRWL 620

L+ E+ SL LD ++G + E + L+ AL VP SW LA+PS +PLG WL

Sbjct 4345 MLLAEMKRSLLELDLGLKGDLTMTEPMERLLKALATDAVPGSWRNLAYPSL--RPLGSWL 4402

Query 621 LGLYARRAHLEEWAAQGVPPTVTWLGGLFRPQAFVFALLAETSRRTGTPLESLSAVAEVT 800

L AR A L +W A+ P WL GLF PQ+F+ A++ T+RR PL+ + EVT

Sbjct 4403 GNLLARHAQLVDWTAELSTPKAVWLSGLFNPQSFLTAVMQATARRNDWPLDKTVIITEVT 4462

Query 801 GYDSAEQVGAPPENGVYVHGAALEGANWDASAGKLAEA 914

+Q+ A +G ++HG LEGA WD G L ++

Sbjct 4463 -KKQPDQIEANSRDGAFIHGLTLEGARWDDKIGALDDS 4499

Blastx GS03181 against *C. reinhardtii*

>ref|XP_001695126.1| UniGene infoGene info flagellar outer dynein arm heavy chain beta [Chlamydomonas reinhardtii]

gb|EDP01834.1| Gene info flagellar outer dynein arm heavy chain beta [Chlamydomonas reinhardtii]

Length=4568

GENE ID: 5720753 ODA4 | flagellar outer dynein arm heavy chain beta

[Chlamydomonas reinhardtii] (10 or fewer PubMed links)

Score = 95.5 bits (236), Expect = 2e-20

Identities = 53/147 (36%), Positives = 78/147 (53%), Gaps = 1/147 (0%)

Frame = +2

Query 137 GLAAFSRAVAPLVEAAGLSGRATFWLAGASESLDLPTLVRLGEWVSHGDVDGLFSSEVVG 316

G+ F + L AG G +L ++ + LV + + +S G + LF+ E

Sbjct 2910 GINDFKENLLGLYRKAGTKGTPITFLMTDNQIVKEGFLVYINDLLSTGYIADLFTPEDKE 2969

Query 317 RIVERVRPQVRSAGLIDSHDSCWDFFVARARENLHLALCLSPEGETGLRSLSRSVPALVS 496

VR +V++AG++DS ++CWDFF+ + R+ LH+ LC SP G+ R +R PALV+

Sbjct 2970 AFTNAVRNEVKAAGILDSAENCWDFFIDKVRKFLHIVLCFSPVGDK-FRIRARQFPALVN 3028

Query 497 GCAVHWFRPWPREALVEVAHTLLRDTP 577

WF WP EALV VA L D P

Sbjct 3029 CTMFDWFHGWPGEALVSVAQRFLVDVP 3055

GS03181 might come from same gene as either GS01613 or GS00095 but GS01613 and GS00095 are distinct

Blastx GS03727 against *C. reinhardtii*

>ref|XP_001703170.1| UniGene infoGene info flagellar inner arm dynein 1 heavy chain alpha [Chlamydomonas

reinhardtii]

gb|EDO96546.1| Gene info flagellar inner arm dynein 1 heavy chain alpha [Chlamydomonas

reinhardtii]

Length=4625

GENE ID: 5728710 PF9 | flagellar inner arm dynein 1 heavy chain alpha

[Chlamydomonas reinhardtii] (10 or fewer PubMed links)

Score = 280 bits (716), Expect = 4e-76

Identities = 129/212 (60%), Positives = 163/212 (76%), Gaps = 0/212 (0%)

Frame = +2

Query 2 SGEIAMSSELDELGNNLFNGQLPDLWRKLTPATDKMLGSWVVFHLRRHAQYAAWVESGEP 181

SGEI SS L+EL ++L+NG+LP +W +L PAT+K LG+W+++ RR+ QY W E GEP

Sbjct 4414 SGEIGFSSRLEELASSLYNGKLPAMWARLNPATEKALGAWMLWFGRRYRQYKDWTEHGEP 4473

Query 182 KVMWLSGLQIPETYTAALVQTTCRRYGWPLDKSSLYTKVTSFTSAAEIGERLQDGCYVEG 361

KV+WLSGL IPETY AALVQ CR GWPLDKS+LYTKVT FT ++ ER + GCY+ G

Sbjct 4474 KVIWLSGLHIPETYIAALVQAACRDKGWPLDKSTLYTKVTKFTDPYQVSERPKYGCYMSG 4533

Query 362 LYLEGAAWDAARRCLCRQPPKVLVQELPIVQIIPVELSKLRLQGTIRVPLYITQQRRNAM 541

LYLEGAAWD L +Q PKVLV ELPI+Q+IP+E +KL+L T R P+Y+TQ RRNAM

Sbjct 4534 LYLEGAAWDLEASQLRKQDPKVLVNELPILQVIPIEANKLKLANTFRAPVYVTQARRNAM 4593

Query 542 GVGMMMECDVDTADHESHWVLQGTALVMNTDQ 637

GVG++ + D+ +A+H SHWVLQG ALV+N DQ

Sbjct 4594 GVGLVFDADLASAEHSSHWVLQGVALVLNIDQ 4625

Blastx GS02579 against *C. reinhardtii*

e02579.1

>ref|XP_001692717.1| UniGene infoGene info flagellar inner arm dynein 1 heavy chain beta [Chlamydomonas

reinhardtii]

gb|EDP03736.1| Gene info flagellar inner arm dynein 1 heavy chain beta [Chlamydomonas

reinhardtii]

Length=4525

GENE ID: 5718404 IDA2 | flagellar inner arm dynein 1 heavy chain beta

[Chlamydomonas reinhardtii] (10 or fewer PubMed links)

Score = 295 bits (755), Expect = 1e-80

Identities = 133/215 (61%), Positives = 171/215 (79%), Gaps = 0/215 (0%)

Frame = +1

Query 1 KGIRGLVVMSAELDDIFQRLLVGAVPPSWLSAYPSLKPLASWARDLIQRWQQLMDWCDRG 180

+GI+GLVVMSA+LD IF+ L VP +WL YPSLKPL W RDL+QR +QL W +

Sbjct 4311 RGIKGLVVMSADLDLIFESLYAAKVPAAWLKTYPSLKPLGPWTRDLLQRIEQLATWVEET 4370

Query 181 MPTVFWLAGFTYPTGFLTALMQTAARENTVSVDSLSWDFPIVGAEEASLTERPKDGAYVK 360

P V+WL+GFTYPTGFLTA++QT AR+ +V +D+LS++F I+ +E + PK+G Y+K

Sbjct 4371 YPRVYWLSGFTYPTGFLTAVLQTTARKASVPIDTLSFEFSIINLDEREINAPPKEGVYIK 4430

Query 361 GLFLEGAGWQHEEGCLCEPAPMQLIDHMPIVHFRPVETRRSKAKGVYSCPLYLYPLRTGS 540

GLFLEGAGW E GCLCEP PM+LI MPI+ FRPVE ++ AKG+Y+CPLYLYPLRTG+

Sbjct 4431 GLFLEGAGWDFENGCLCEPNPMELIVPMPILLFRPVENKKRTAKGIYTCPLYLYPLRTGT 4490

Query 541 RERPSFMLNVDLKSGAAEPEAWVKRGTALLLALAS 645

RERPSFM+NVDL+SG+A+P+ W+ RGTALLL+LA+

Sbjct 4491 RERPSFMINVDLRSGSADPDHWIMRGTALLLSLAT 4525

e02579.2

>ref|XP_001692717.1| UniGene infoGene info flagellar inner arm dynein 1 heavy chain beta [Chlamydomonas

reinhardtii]

gb|EDP03736.1| Gene info flagellar inner arm dynein 1 heavy chain beta [Chlamydomonas

reinhardtii]

Length=4525

GENE ID: 5718404 IDA2 | flagellar inner arm dynein 1 heavy chain beta

[Chlamydomonas reinhardtii] (10 or fewer PubMed links)

Score = 273 bits (697), Expect = 5e-74

Identities = 125/213 (58%), Positives = 161/213 (75%), Gaps = 0/213 (0%)

Frame = +3

Query 3 KAGESDGSAMHVVLVQELQRYNKLLAAIRLSLADVRKGIRGLVVMSAELDDIFQRLLVGA 182

KA D SA+HVVL QE++RYN LL A+R S ++++GI+GLVVMSA+LD IF+ L

Sbjct 4275 KAKADDPSALHVVLFQEVERYNALLVAVRRSCVELQRGIKGLVVMSADLDLIFESLYAAK 4334

Query 183 VPPSWLSAYPSLKPLASWARDLIQRWQQLMDWCDRGMPTVFWLAGFTYPTGFLTALMQTA 362

VP +WL YPSLKPL W RDL+QR +QL W + P V+WL+GFTYPTGFLTA++QT

Sbjct 4335 VPAAWLKTYPSLKPLGPWTRDLLQRIEQLATWVEETYPRVYWLSGFTYPTGFLTAVLQTT 4394

Query 363 ARENTVSVDSLSWDFPIVGAEEASLTERPKDGAYVKGLFLEGAGWQHEEGCLCEPAPMQL 542

AR+ +V +D+LS++F I+ +E + PK+G Y+KGLFLEGAGW E GCLCEP PM+L

Sbjct 4395 ARKASVPIDTLSFEFSIINLDEREINAPPKEGVYIKGLFLEGAGWDFENGCLCEPNPMEL 4454

Query 543 IDHMPIVHFRPVETRRSKAKGVYSCPLYLYPLR 641

I MPI+ FRPVE ++ AKG+Y+CPLYLYPLR

Sbjct 4455 IVPMPILLFRPVENKKRTAKGIYTCPLYLYPLR 4487

Blastx GS06225 against *C. reinhardtii*

>ref|XP_001692717.1| UniGene infoGene info flagellar inner arm dynein 1 heavy chain beta [Chlamydomonas

reinhardtii]

gb|EDP03736.1| Gene info flagellar inner arm dynein 1 heavy chain beta [Chlamydomonas

reinhardtii]

Length=4525

GENE ID: 5718404 IDA2 | flagellar inner arm dynein 1 heavy chain beta

[Chlamydomonas reinhardtii] (10 or fewer PubMed links)

Score = 205 bits (521), Expect = 1e-53

Identities = 107/164 (65%), Positives = 134/164 (81%), Gaps = 1/164 (0%)

Frame = +1

Query 1 LMIDPQEQANRWIRNMEKENG-LKVVTLKQSDYLRTLENAIAFGQPvllqeveeelDPSL 177

LMIDPQ QAN+WI+NME G LKV+ L+ SD R +ENAI FGQPVL+Q++ +E+DP L

Sbjct 3490 LMIDPQGQANKWIKNMEGRGGRLKVLNLQMSDMARQIENAIQFGQPVLMQDILQEIDPIL 3549

Query 178 EPIMSRAVVKVGNRSILKLGDKEVDYNPEFRFYLTTKLANPHYTPEISTKACLVNFCVKQ 357

EP+++++ +K GN++++KLGDKEVDYN +FR YLTTKLANP YTPEISTK +VNF VK+

Sbjct 3550 EPVLAKSFIKRGNQTLIKLGDKEVDYNFDFRLYLTTKLANPLYTPEISTKVMIVNFAVKE 3609

Query 358 QGLEDQLLGIVVRKERPDLEAQKNELVVAVAAGKRKLVELEDTV 489

QGLE QLL VV+ ERPDL+ QKN+LVV VAAGKR ELEDT+

Sbjct 3610 QGLEAQLLATVVKNERPDLDKQKNDLVVKVAAGKRTQAELEDTI 3653

Score = 83.2 bits (204), Expect = 7e-17

Identities = 35/63 (55%), Positives = 47/63 (74%), Gaps = 0/63 (0%)

Frame = +1

Query 484 TVSVDSLSWDFPIVGAEEASLTERPKDGAYVKGLFLEGAGWQHEEGCLCEPAPMQLIDHM 663

+V +D+LS++F I+ +E + PK+G Y+KGLFLEGAGW E GCLCEP PM+LI M

Sbjct 4399 SVPIDTLSFEFSIINLDEREINAPPKEGVYIKGLFLEGAGWDFENGCLCEPNPMELIVPM 4458

Query 664 PIV 672

PI+

Sbjct 4459 PIL 4461

Blastx GS07942 against *C. reinhardtii*

>ref|XP_001692717.1| UniGene infoGene info flagellar inner arm dynein 1 heavy chain beta [Chlamydomonas

reinhardtii]

gb|EDP03736.1| Gene info flagellar inner arm dynein 1 heavy chain beta [Chlamydomonas

reinhardtii]

Length=4525

GENE ID: 5718404 IDA2 | flagellar inner arm dynein 1 heavy chain beta

[Chlamydomonas reinhardtii] (10 or fewer PubMed links)

Score = 55.1 bits (131), Expect = 2e-08

Identities = 25/42 (59%), Positives = 33/42 (78%), Gaps = 0/42 (0%)

Frame = +2

Query 446 YHLHVVSHERASFMLNVDLVSGAAEPEEWVKRGTALLLALAS 571

Y L + ER SFM+NVDL SG+A+P+ W+ RGTALLL+LA+

Sbjct 4484 YPLRTGTRERPSFMINVDLRSGSADPDHWIMRGTALLLSLAT 4525

>sp|Q9MBF8.1|DYH1B_CHLRE RecName: Full=Dynein-1-beta heavy chain, flagellar inner arm

I1 complex; AltName: Full=1-beta DHC; AltName: Full=Dynein-1,

subspecies f

emb|CAB99316.1| Geo 1 beta dynein heavy chain [Chlamydomonas reinhardtii]

Length=4513

Score = 55.1 bits (131), Expect = 2e-08

Identities = 25/42 (59%), Positives = 33/42 (78%), Gaps = 0/42 (0%)

Frame = +2

Query 446 YHLHVVSHERASFMLNVDLVSGAAEPEEWVKRGTALLLALAS 571

Y L + ER SFM+NVDL SG+A+P+ W+ RGTALLL+LA+

Sbjct 4472 YPLRTGTRERPSFMINVDLRSGSADPDHWIMRGTALLLSLAT 4513

Note: GS07942 has stop codon in reading frame with homology, right before start of homology.

Clustal alignments of GS02569, GS06225, GS07942, and *C. reinhardtiii* DHC1b

CLUSTAL W (1.83) multiple sequence alignment

DHCb1_CHLRE MEPGDEGKGHQLTADATCIAWVRSKLQLLKPESLGDSDGAEWLSSVWHNDVHTPVVSTFL

GS06225 ------------------------------------------------------------

GS02579 ------------------------------------------------------------

GS07942 ------------------------------------------------------------

DHCb1_CHLRE MSVKATRMFAALDGGHEGGSSPKLVLALEVPKQFEQMVYFVRDPSKFVTRENVGSVIFFG

GS06225 ------------------------------------------------------------

GS02579 ------------------------------------------------------------

GS07942 ------------------------------------------------------------

DHCb1_CHLRE VMRGGDPLHSLLNIMHGLYVPVVVANTTWPETVKSDFTAQMHKFMANLTETVYEVKGKTI

GS06225 ------------------------------------------------------------

GS02579 ------------------------------------------------------------

GS07942 ------------------------------------------------------------

DHCb1_CHLRE LYIPQEDLRDPKAAAKQKDLVQRLESTIIHWTRQVKELLNQQDSVDASEQAGPLAEIEFW

GS06225 ------------------------------------------------------------

GS02579 ------------------------------------------------------------

GS07942 ------------------------------------------------------------

DHCb1_CHLRE RERSVDLSGIRAQLDDGAVSSIVSVLEYAKSSYLAPFLSLRNLIHREAVAAEDNLKFLLC

GS06225 ------------------------------------------------------------

GS02579 ------------------------------------------------------------

GS07942 ------------------------------------------------------------

DHCb1_CHLRE LEEPCQQLASAHPQTIPSLLPPILNCIRMVWNLSRFYNTPERLSVLLRKLSNEIINRCCS

GS06225 ------------------------------------------------------------

GS02579 ------------------------------------------------------------

GS07942 ------------------------------------------------------------

DHCb1_CHLRE VISLPDVWSGDVDNVMVALRQSMEAGERWKELYKRTAAAVAVRSPKPWDFDISSIFAHID

GS06225 ------------------------------------------------------------

GS02579 ------------------------------------------------------------

GS07942 ------------------------------------------------------------

DHCb1_CHLRE AFLQRCNDLLEVCEAQLQFAPRTPLPVFGGTYGPEVKKSILDIQESFQGLVQGLQALKYD

GS06225 ------------------------------------------------------------

GS02579 ------------------------------------------------------------

GS07942 ------------------------------------------------------------

DHCb1_CHLRE ILDVKATRWHDDFNGFKGGVKDLEVMMANVIQRAFDTQPCLAARGELLEGFQTMAKRDYI

GS06225 ------------------------------------------------------------

GS02579 ------------------------------------------------------------

GS07942 ------------------------------------------------------------

DHCb1_CHLRE RRFVEKKTVEFFALFNAEINTVKKLFDAVKRSQPKSPILPRYAGLAKYAMNLMRRLEQSH

GS06225 ------------------------------------------------------------

GS02579 ------------------------------------------------------------

GS07942 ------------------------------------------------------------

DHCb1_CHLRE KVVDSVRYTLPQVSEAADVMQQYELAHQAIEQYISNTHNDWFSTIESSIAKELQACLLTQ

GS06225 ------------------------------------------------------------

GS02579 ------------------------------------------------------------

GS07942 ------------------------------------------------------------

DHCb1_CHLRE DKASGGLLSMNFHKDLLSMGQEVHFWERMRLAVPLVAMEINAQREKYRVLRDNILMVVRD

GS06225 ------------------------------------------------------------

GS02579 ------------------------------------------------------------

GS07942 ------------------------------------------------------------

DHCb1_CHLRE YNKILTALDKEERKLFHDRIRYLDRRIMPGVTKLQWTADKHALEFYYREARKFCRDADMA

GS06225 ------------------------------------------------------------

GS02579 ------------------------------------------------------------

GS07942 ------------------------------------------------------------

DHCb1_CHLRE VGDYKTANSRLDAICRSISELVLVDVEKKKIYQHAEFANLQESHHAKIKDRLVSAVDEIR

GS06225 ------------------------------------------------------------

GS02579 ------------------------------------------------------------

GS07942 ------------------------------------------------------------

DHCb1_CHLRE DIMASIHRVFEQDSEEVQREWVRFTQKVDRKLEDALRHVIKKSLQELSRLLNGDNKTEVM

GS06225 ------------------------------------------------------------

GS02579 ------------------------------------------------------------

GS07942 ------------------------------------------------------------

DHCb1_CHLRE PIFHVTMVLERTNRVELRPTIQALFDTINSVARNLILVLQSVPRVALQLTDKQRRDMEDA

GS06225 ------------------------------------------------------------

GS02579 ------------------------------------------------------------

GS07942 ------------------------------------------------------------

DHCb1_CHLRE GLPLPKPLPTLYETISADEDAVLRTIMQITSGITSIIDKVQAFLTYWEKKYRQVWEADRD

GS06225 ------------------------------------------------------------

GS02579 ------------------------------------------------------------

GS07942 ------------------------------------------------------------

DHCb1_CHLRE AYIRRYEKAQKPLSSFEADISRYLQCIDEIRGEDGATNMRFLRIDCGPLKLTLVGHCEAW

GS06225 ------------------------------------------------------------

GS02579 ------------------------------------------------------------

GS07942 ------------------------------------------------------------

DHCb1_CHLRE VSKFTGLLGQLAATELRTLHTYFRENKDSLMLAPSTLEQLAELVGLHRRLADERRRTEAR

GS06225 ------------------------------------------------------------

GS02579 ------------------------------------------------------------

GS07942 ------------------------------------------------------------

DHCb1_CHLRE FEPLRDKYKLLERYEVGAKEEEAALLEGLEPAWTQFQALLDETAGKLERYKDNFREKVKS

GS06225 ------------------------------------------------------------

GS02579 ------------------------------------------------------------

GS07942 ------------------------------------------------------------

DHCb1_CHLRE LLDTFLKDVAQLCEDFSRDAPYSSEVPTPDALDFIQASKQADEDTRKRAAEIKNGMDIFN

GS06225 ------------------------------------------------------------

GS02579 ------------------------------------------------------------

GS07942 ------------------------------------------------------------

DHCb1_CHLRE IPQPQYKDLAAMEKDLDFLDRIWGLKDEWEQLYYGWKDGSFTDIKVEEMEEAAVRIGKNV

GS06225 ------------------------------------------------------------

GS02579 ------------------------------------------------------------

GS07942 ------------------------------------------------------------

DHCb1_CHLRE AKLGRDIRQWTVWSSLKDTLDAFKRTMPLITDLRNPAMRPRHWQNLQDHIGVRFDPHSRD

GS06225 ------------------------------------------------------------

GS02579 ------------------------------------------------------------

GS07942 ------------------------------------------------------------

DHCb1_CHLRE FTLDSLVALRLDQHVEFVAELSVNATKELAIENNIKAIAATWSALGLDMAEYKSTFKLRS

GS06225 ------------------------------------------------------------

GS02579 ------------------------------------------------------------

GS07942 ------------------------------------------------------------

DHCb1_CHLRE TEEIFTSLEENIVTLSTMKASKYFIVFEKDIAYWEKTLSHISETIEIILQVQRNWMYLEN

GS06225 ------------------------------------------------------------

GS02579 ------------------------------------------------------------

GS07942 ------------------------------------------------------------

DHCb1_CHLRE IFIGSEDIRKQLPQESQMFDAVHNNFMRLMKQLYSTANCLKACTAQGLLESFQDMNNKLE

GS06225 ------------------------------------------------------------

GS02579 ------------------------------------------------------------

GS07942 ------------------------------------------------------------

DHCb1_CHLRE RIQKSLDNYLENKRQQFPRFYFLSSDDLLEILGQAKDPLNVQPHLKKCFEGIKKLDMHLP

GS06225 ------------------------------------------------------------

GS02579 ------------------------------------------------------------

GS07942 ------------------------------------------------------------

DHCb1_CHLRE GEDRKQTISVGITSPDGEYLPFANPVITEGRPEEWLNRVEDAMFLTTKKHLYKVLEESKA

GS06225 ------------------------------------------------------------

GS02579 ------------------------------------------------------------

GS07942 ------------------------------------------------------------

DHCb1_CHLRE QKKEKWVKENQGQMIITAGQIVWTHECEKALADADSARKNLKLLKKKWISYLNKLTAVTR

GS06225 ------------------------------------------------------------

GS02579 ------------------------------------------------------------

GS07942 ------------------------------------------------------------

DHCb1_CHLRE SKLNKIERNKVVALITIEVHARDVIEKLGKSNCSSTNDFEWVSQLRFYWDREKNDCIVKQ

GS06225 ------------------------------------------------------------

GS02579 ------------------------------------------------------------

GS07942 ------------------------------------------------------------

DHCb1_CHLRE VLSVFYYGYEYQGNNGRLVITPLTDRCYMTLGAAMFTRRGGNPLGPAGTGKTETVKDFGK

GS06225 ------------------------------------------------------------

GS02579 ------------------------------------------------------------

GS07942 ------------------------------------------------------------

DHCb1_CHLRE ALARYVIVFNCSDGVDYKMTGKMFSGLAQTGAWACLDEFNRIEVEVLSVVATQIAAVMQA

GS06225 ------------------------------------------------------------

GS02579 ------------------------------------------------------------

GS07942 ------------------------------------------------------------

DHCb1_CHLRE IKESKKRFLFLGQEIRLNPSCGIFVTMNPGYAGRSELPDNLKAMLRPVSMMVPDFTLIAE

GS06225 ------------------------------------------------------------

GS02579 ------------------------------------------------------------

GS07942 ------------------------------------------------------------

DHCb1_CHLRE IMMFSEGFSSAKVLAKKMIAIMELSQQQLSKQDHYDYGLRSFVIPIARAAGSLKRLDPEG

GS06225 ------------------------------------------------------------

GS02579 ------------------------------------------------------------

GS07942 ------------------------------------------------------------

DHCb1_CHLRE SEEVILYRTMLDLIKPKLVYLDLPLFMALLSDLFPGVELPPADGGSLRRAIEAELRESNL

GS06225 ------------------------------------------------------------

GS02579 ------------------------------------------------------------

GS07942 ------------------------------------------------------------

DHCb1_CHLRE QIVPEFVTKIIQVFDCKVARHGNMIVGRTGSGKSEAWKCLQRALGRLRKEEPDDDRFQKV

GS06225 ------------------------------------------------------------

GS02579 ------------------------------------------------------------

GS07942 ------------------------------------------------------------

DHCb1_CHLRE HVHTINPLALSNDELYGCFEAATHEWQDGVLARIMRTVCKDETHEQKWILFDGPVDTLWI

GS06225 ------------------------------------------------------------

GS02579 ------------------------------------------------------------

GS07942 ------------------------------------------------------------

DHCb1_CHLRE ESMNTTLDDNKLLTLLSGERIAMTPAVSLLFEVEDLSQASPATVSRAGMIYLNVEDLGWR

GS06225 ------------------------------------------------------------

GS02579 ------------------------------------------------------------

GS07942 ------------------------------------------------------------

DHCb1_CHLRE PFITSWLAAKQAAPGADAAIIDQVSKLVDKYMEAALEHKRLHCRELVPTDRLSCVRAFTR

GS06225 ------------------------------------------------------------

GS02579 ------------------------------------------------------------

GS07942 ------------------------------------------------------------

DHCb1_CHLRE LWDALAVPENGVGTMPVDESAGPPGSKAAAAAAAAAAAAAPPEETSGGTGGNLVEMWFLF

GS06225 ------------------------------------------------------------

GS02579 ------------------------------------------------------------

GS07942 ------------------------------------------------------------

DHCb1_CHLRE CLIWGIGGPLDEEGRKKFDAFMREMDTRYPSSDTVFEYFVEPKAKSWLAWETKLTGAFKP

GS06225 ------------------------------------------------------------

GS02579 ------------------------------------------------------------

GS07942 ------------------------------------------------------------

DHCb1_CHLRE AMDQPFFKILVPTVDTVRNRFVGSALVRVSQHTLIVGNVGVGKTMIVGSLLEGLPGDRMS

GS06225 ------------------------------------------------------------

GS02579 ------------------------------------------------------------

GS07942 ------------------------------------------------------------

DHCb1_CHLRE SMTINFSAQTSSNSLQDTIEGKLEKRTKGVFAPAGGKRLVCFIDDLNMPQKSKFGFIPPL

GS06225 ------------------------------------------------------------

GS02579 ------------------------------------------------------------

GS07942 ------------------------------------------------------------

DHCb1_CHLRE ELLKLWVDNGFWYDRAKCEVKHIKDMQLLAAMAPPGGGRNAFSQRVQACFATLNVTAPND

GS06225 ------------------------------------------------------------

GS02579 ------------------------------------------------------------

GS07942 ------------------------------------------------------------

DHCb1_CHLRE NQLKRIFGTILNAKLADFDDEVKPLSEPITMATIGIYRAVSKELLPTPSKSHYLFNTRDL

GS06225 ------------------------------------------------------------

GS02579 ------------------------------------------------------------

GS07942 ------------------------------------------------------------

DHCb1_CHLRE AKIIQGMMQATKAFYNSKEEVLQLWCHECMRIIADRMWDHADKEWLVRQLDEKLGTTFST

GS06225 ------------------------------------------------------------

GS02579 ------------------------------------------------------------

GS07942 ------------------------------------------------------------

DHCb1_CHLRE SFGTLFEAYNETVPPFVTFMRQNVDVPVYEAVRDMVALKDLLTERLEDYALEPGHSAMDL

GS06225 ------------------------------------------------------------

GS02579 ------------------------------------------------------------

GS07942 ------------------------------------------------------------

DHCb1_CHLRE VLFRDALSHVCRIHRILGQPRGNALLVGVGGSGRKSLARLAAFVAELKCFTIEITKNYRQ

GS06225 ------------------------------------------------------------

GS02579 ------------------------------------------------------------

GS07942 ------------------------------------------------------------

DHCb1_CHLRE TEFREDLKGLYRQAGVANKPTVFLFDETQIVYETFLEDVNNILTSGEVPNLFPKDELGSV

GS06225 ------------------------------------------------------------

GS02579 ------------------------------------------------------------

GS07942 ------------------------------------------------------------

DHCb1_CHLRE LDELRPAAKAAGAGETADALYGFLLERVRTNLHVVLCLSPVGEAFRERCRMFPGLVNCTT

GS06225 ------------------------------------------------------------

GS02579 ------------------------------------------------------------

GS07942 ------------------------------------------------------------

DHCb1_CHLRE IDWFTEWPADALFEVAQKQLMDVDLGSTEVKTAVCKVFVTAHQSVENTSAKMFAALKRRN

GS06225 ------------------------------------------------------------

GS02579 ------------------------------------------------------------

GS07942 ------------------------------------------------------------

DHCb1_CHLRE YVTPTNYLETVRGYKGLLAEKRTELGEKAAKLQGGLHKLDETSVQVAAMKKVAEEKKVVV

GS06225 ------------------------------------------------------------

GS02579 ------------------------------------------------------------

GS07942 ------------------------------------------------------------

DHCb1_CHLRE AQAKADCEELLVEIVQDKRVADEQEKQVNAEAQKIGKEAEEANIIAAQVQQELDKALPAL

GS06225 ------------------------------------------------------------

GS02579 ------------------------------------------------------------

GS07942 ------------------------------------------------------------

DHCb1_CHLRE REAEAALDVLTKKDMSELKAYAKPPEKVEMTLNAVLTVLRRPPNWDEAKKRLSDANFMQS

GS06225 ------------------------------------------------------------

GS02579 ------------------------------------------------------------

GS07942 ------------------------------------------------------------

DHCb1_CHLRE LKEFDKDKLDDSLLKKIGKFTANPDFTYEKINTVSAAASGMCKWVHAMETYGYVAKDVAP

GS06225 ------------------------------------------------------------

GS02579 ------------------------------------------------------------

GS07942 ------------------------------------------------------------

DHCb1_CHLRE KRAKLKSAQDTLARKQAALALAQEQLAVVLAKVQALKDKYDTSIARKQALEEELADLEGK

GS06225 ------------------------------------------------------------

GS02579 ------------------------------------------------------------

GS07942 ------------------------------------------------------------

DHCb1_CHLRE LERAEKLVTGLAGERVRWEASISEYNIALGCLPGDVVVAAAFMSYAGPFPSEYRDELVKH

GS06225 ------------------------------------------------------------

GS02579 ------------------------------------------------------------

GS07942 ------------------------------------------------------------

DHCb1_CHLRE TWLPQVKALNIPASEHFDFALFLANPAMVRDWNIQGLPSDSFSTENGVMVTRGRRWPLMI

GS06225 ------------------------------------------------------------

GS02579 ------------------------------------------------------------

GS07942 ------------------------------------------------------------

DHCb1_CHLRE DPQGQANKWIKNMEGRGGRLKVLNLQMSDMARQIENAIQFGQPVLMQDILQEIDPILEPV

GS06225 ------------------------------------------------------------

GS02579 ------------------------------------------------------------

GS07942 ------------------------------------------------------------

DHCb1_CHLRE LAKSFIKRGNQTLIKLGDKEVDYNFDFRLYLTTKLANPLYTPEISTKVMIVNFAVKEQGL

GS06225 ------------------------------------------------------------

GS02579 ------------------------------------------------------------

GS07942 ------------------------------------------------------------

DHCb1_CHLRE EAQLLATVVKNERPDLDKQKNDLVVKVAAGKRTQAELEDTILHLLSTATGSLLDNVTLIN

GS06225 ------------------------------------------------------------

GS02579 ------------------------------------------------------------

GS07942 ------------------------------------------------------------

DHCb1_CHLRE TLDQSKTTWEEVNASLAVAEETQKKIEAASQLYRPCSVRASVLYFVLNDLSTIDPMYQFS

GS06225 ------------------------------------------------------------

GS02579 ------------------------------------------------------------

GS07942 ------------------------------------------------------------

DHCb1_CHLRE LDAYNDLFLLSIKNSPKNDNLAERIKSLNDFHTYAVYKYTSRGLFERHKLLLSLQMCVRI

GS06225 ------------------------------------------------------------

GS02579 ------------------------------------------------------------

GS07942 ------------------------------------------------------------

DHCb1_CHLRE LQTANQVNTEEWQFFLRGGTVLDRSSQPNNPSQEWISEEAWDNITELDALPNFKGVVSSF

GS06225 ------------------------------------------------------------

GS02579 ------------------------------------------------------------

GS07942 ------------------------------------------------------------

DHCb1_CHLRE ESNLGEWEAWYRKGDPEASELPAEWESKCNELQRLILVRCLRPDRVIFAATTYVSNALGR

GS06225 ------------------------------------------------------------

GS02579 ------------------------------------------------------------

GS07942 ------------------------------------------------------------

DHCb1_CHLRE KYVEPPVLDLAETLKDSTALSPLIFVLSAGVDPTDNLRKLATEKGMTSRFFTVALGQGQA

GS06225 ------------------------------------------------------------

GS02579 ------------------------------------------------------------

GS07942 ------------------------------------------------------------

DHCb1_CHLRE PTATRLIEDGLREGNWVFLANCHLMTSWLPTLDKIIEGFETKQPHENFRLWLSSNPSPSF

GS06225 ------------------------------------------------------------

GS02579 ------------------------------------------------------------

GS07942 ------------------------------------------------------------

DHCb1_CHLRE PIAILQRGIKMTTEPPKGLRANLLRLYNSVSDASYAQCKTQIKYQKLLFALTYFHSVLLE

GS06225 ------------------------------------------------------------

GS02579 ------------------------------------------------------------

GS07942 ------------------------------------------------------------

DHCb1_CHLRE RRKFRTLGFNIPYDFNDTDFSVSDDLLKSYLDSYEQTPWDALKYLIAEANYGGRVTDELD

GS06225 ------------------------------------------------------------

GS02579 ------------------------------------------------------------

GS07942 ------------------------------------------------------------

DHCb1_CHLRE RRVLASYLNKFYCEDALAVPGYLLSPLSTYYVPENGPLQSFKDYILTLPAGDRPEAFGQH

GS06225 ------------------------------------------------------------

GS02579 ------------------------------------------------------------

GS07942 ------------------------------------------------------------

DHCb1_CHLRE PNAEISYLIEDSKVLLDSLLSLQPRTEGAAGGAGTRREDVVMAIATDLLDQVPQPFNLEE

GS06225 -----------------------------------------------------------L

GS02579 ------------------------------------------------------------

GS07942 ------------------------------------------------------------

DHCb1_CHLRE VMKAKADDPSALHVVLFQEVERYNALLVAVRRSCVELQRGIKGLVVMSADLDLIFESLYA

GS06225 MIDPQEQANRWIRNMEKENGLKVVTLKQSDYLRTLENAIAFGQPVLLQEVEEELDPSLEP

GS02579 --KAGESDGSAMHVVLVQELQRYNKLLAAIRLSLADVRKGIRGLVVMSAELDDIFQRLLV

GS07942 ------------------------------------------------------------

DHCb1_CHLRE AKVPAAWLKTYPSLKPLGPWTRDLLQRIEQLATWVEETYPRVYWLSGFTYPTGFLTAVLQ

GS06225 IMSRAVVKVGNRSILKLGDKEVDYNPEFRFYLTTKLANPHYTPEISTKACLVNFCVKQQG

GS02579 GAVPPSWLSAYPSLKPLASWARDLIQRWQQLMDWCDRGMPTVFWLAGFTYPTGFLTALMQ

GS07942 ------------------------------------------------------------

DHCb1_CHLRE TTARKASVPIDTLSFEFSIINLDEREINAPPKEGVYIKGLFLEGAGWDFENGCLCEPNPM

GS06225 LEDQLLGIVVRKERPDLEAQKNELVVAVAAGKRKLVELEDTVSVDSLSWDFPIVGAE---

GS02579 TAARENTVSVDSLSWDFPIVGAEEASLTERPKDGAYVKGLFLEGAGWQHEEGCLCEPAPM

GS07942 ---------------------------------------------------GLPCS----

DHCb1_CHLRE ELIVPMPILLFRPVENKKRTAKGIYTCPLYLYPLRTGTRERPSFMINVDLRSGSADPDHW

GS06225 -------------------------------EASLTERPKDGAYVKGLFLEGAGWQHEEG

GS02579 QLIDHMPIVHFRPVETRRSKAKGVYSCPLYLYPLRTGSRERPSFMLNVDLKSGAAEPEAW

GS07942 -------------------------------YHLHVVSHERASFMLNVDLVSGAAEPEEW

. : ::: .: * ... : :

DHCb1_CHLRE IMRGTALLLSLAT----

GS06225 CLCEPAPMQLIDHMPIV

GS02579 VKRGTALLLALAS----

GS07942 VKRGTALLLALAS----

.* : :

Note that last amino acids of GS02579 and GS07942 are nearly identical… check nucleotides

CLUSTAL W (1.83) multiple sequence alignment

e07942.1 -------------------------------AGGAGCAGAGCGA-ACTACTCGGCCGATG

e02579.1 AAGGGGATCCGCGGGCTCGTGGTCATGTCGGCCGAGCTGGACGACATCTTCCAGCGGCTG

**** * *** * * ** * **

e07942.1 GTGG-GAAAGCTGTGCCGACA-CGCGCCAGTGCGCTGTCTTGTGCGACCAGCTTCCGGCA

e02579.1 CTCGTGGGCGCCGTGCCGCCCTCGTGGCTGTCGGCCTACCCGTCGCTCAAGCCGCTGGCC

* * * ** ****** * ** * * ** ** * ** * *** * ***

e07942.1 CCGTTCGT-CGCCGGCTG--CCGGCTTTT-CACCTCCTG--GGGCTGG-ACGCGCGGAGA

e02579.1 TCGTGGGCGCGCGACCTGATCCAGCGATGGCAGCAGCTGATGGACTGGTGCGATCGGGGA

*** * *** *** ** ** * ** * *** ** **** ** *** **

e07942.1 C--CCGTCGCGGCTCACGTTCAGGAAGGTGGCATGCCCAGACGCCTTCAGAGCGCACGTC

e02579.1 ATGCCGACCGTGTTCTGGCTGGCGGGCTTCACCTACCCGACCGGCTTTCTCAC-TGCGCT

*** * * ** * * * * * * *** ** *** * **

e07942.1 AGCGCACAATTCGGGCCGGAGGGGC-CGGTGCTGCAGACCTCTCCGCCACCGTCCCCCGC

e02579.1 CATGCAGACGGCGGCGCGCGAGAACACCGTGTCGGTGGACTCGCTCTCGTGGGACTTC--

*** * *** ** * * * *** * * *** * * * * *

e07942.1 TCCGCCGCCGCCGCCGCCGACTTCTCCCCTCC----CGTCCAT--GCCGCCACCCCCTTC

e02579.1 CCCATCGTCGGTGCCGAGGAGGCCTCCCTCACGGAGCGTCCAAAGGACGGCGCGTACGTC

** ** ** **** ** ***** * ****** * ** * * * **

e07942.1 TC---CTGCTCCCTCACGACCGCCG-----CCGCATCCTTGCGGC-GCCGGCGTGATC--

e02579.1 AAGGGCCTCTTCCTCGAGGGAGCCGGGTGGCAGCATGAGGAAGGCTGCCTGTGCGAGCCG

* ** **** * **** * **** *** *** * * ** *

e07942.1 ------GTGCACCTGA---ACTATCTGTCTACTGT------CTGGACGGCTAGAGC-AGC

e02579.1 GCGCCAATGCAGCTGATCGACCACATGCCGATTGTGCATTTTCGGCCGGTCGAGACGAGG

**** **** ** * ** * * *** ** *** * **

e07942.1 CACAGCACGTGCGCGGCCGGATGCTAGGGGCTCCCCTGCTCCTACCACCTGCACGTTGTG

e02579.1 CGGAGCAAGGCCAAGGGCGTGTACTCGTGCCCGCTCTACCTCTACCCGCTGCGCACGGGC

* **** * * ** ** * ** * * * * ** * ***** **** * *

e07942.1 TCGCATGAGCGAGCATCCTTCATGCTCAACGTCGACCTCGTCTCGGGCGCCGCGGAGCCG

e02579.1 TCGCGCGAGCGACCATCCTTCATGCTCAACGTCGACCTCAAGTCGGGCGCCGCGGAGCCG

**** ****** ************************** ******************

e07942.1 GAGGAGTGGGTCAAGCGCGGCACCGCGCTGCTGCTGGCGCTGGCGTCTTGACCGCGCTGC

e02579.1 GAGGCGTGGGTCAAGCGCGGCACCGCGCTGCTGCTGGCGCTGGCGTCTTGACCGCGCTGC

**** *******************************************************

e07942.1 TGCTGCTGCTGATTGGGCTTGTCTTCTGTG------------------------------

e02579.1 TGCTGCTGCTGATTGGGCTTGTCTTCTGTGTTCAGGGGCGGTATAGACGGCGCCCCGGTT

******************************

e07942.1 ------------------------------------------------------------

e02579.1 CTCGTACGTGCTCGCAATCGCATAGATTGTGACGGAGTTTGCATATGTGTGATTTTCTAT

e07942.1 -------------------------------

e02579.1 TTGTTCTTTTTTGAACCACGGGGTAGTCTTC

Conclusion: GS02569, GS06225 hit to overlapping regions of the same *C. reinhardtiii* protein but differ in amino acid sequence so they are likely to be distinct. GS07942 appears to be an alternate splicing product of the same gene as GS02569.

Blastx GS00012 against *C. reinhardtii*

>ref|XP_001694660.1| UniGene infoGene info dynein heavy chain 2 [Chlamydomonas reinhardtii]

gb|EDP02244.1| Gene info dynein heavy chain 2 [Chlamydomonas reinhardtii]

Length=4069

GENE ID: 5720247 DHC2 | dynein heavy chain 2 [Chlamydomonas reinhardtii]

(10 or fewer PubMed links)

Score = 426 bits (1095), Expect = 1e-119

Identities = 235/548 (42%), Positives = 336/548 (61%), Gaps = 28/548 (5%)

Frame = +1

Query 1 EKIVESFSGNASIHPSFRLWLTSMPAKYFPVPVLQASVKMTFEPPKGLRANLKGTWATVT 180

E+IVE + +H FRLWLTSMP+ FPV +LQ VKMT EPPKGL++NL + +T

Sbjct 3535 ERIVEGIQPDR-VHKDFRLWLTSMPSPDFPVAILQNGVKMTLEPPKGLKSNLVRQYNRLT 3593

Query 181 DKQWDGCSKPLEWRKLLFGVAFFHAVVQERRKFGPLGWNIRYEFNGTDLEMSMETLRMFL 360

D SKP +WR+L+FG+ FHAV+Q+RRKFGPLGWNIRY+F DL +S+ L+ +L

Sbjct 3594 DAYLAASSKPEDWRRLVFGLCLFHAVIQDRRKFGPLGWNIRYDFTDGDLNVSLAQLQEYL 3653

Query 361 DEQEVIPWAALLYVTGQINYGGRVTDDWDRRNLMVALRRFYRPEVLEDGFPFAPAGTTAA 540

D+ EVIP+ L ++ +INYGGRVTDD DRR + + F P VLE G+ F+P+GT

Sbjct 3654 DKYEVIPFKVLRFLFTEINYGGRVTDDKDRRLINNLIYTFCGPSVLEPGYAFSPSGT--- 3710

Query 541 STYYSIAEGGLQAVRDWVDGL---PYDDPVTVFGLHPNAKITFERQETDKLLATVTAIQP 711

Y+ + +VRD ++ L P +FGLH NA IT ++ ET + +TV ++QP

Sbjct 3711 ---YATPPEAVVSVRDHLELLRAYPIVPKPEIFGLHENADITCDQNETYDMFSTVLSLQP 3767

Query 712 RLggggdgLSEEETVVALAEKLKAEVPELLLEENAGAGTFALNEKGELNSLQIVLLAEMG 891

R+ G S+E + ALA + +PE L + +A + K +N+ VL E

Sbjct 3768 RVASGAGQ-SQEAVIGALAADILGRLPE-LFDVDAVIERYPTTYKESMNT---VLTQECI 3822

Query 892 RFNKLLRRVSGSLADLGKAIKGLITMSADLDAMYGAMLKNQVPLLWAKVSYPSLKPLSSW 1071

R+N LL + SL + KA+KGL+ MS +L+ + +M NQVP LWA +YPSLKPLS+W

Sbjct 3823 RYNALLAVMKRSLGETIKALKGLVVMSPELEGVAYSMYDNQVPELWASRAYPSLKPLSAW 3882

Query 1072 FKDLQERVRFMRGWLAAGVSGMACYSLPAFFFPQGFMTGILQMHARRYSIPIDSLSFSYE 1251

DL ER F+ GW+ G + Y + FFFPQ F+TG LQ AR+Y+ PID++SF ++

Sbjct 3883 VVDLLERCAFISGWVDKGTPPV--YWISGFFFPQAFLTGTLQNFARKYTYPIDTVSFGFK 3940

Query 1252 IRteetaaacaappeDGVYIDGFFLDGARWDREKRQLADSRPTVMYDTLPVIHFVPTPHF 1431

+ A + PEDG +I G F++GARWD + +A+SRP ++ +P++ P H

Sbjct 3941 VMDALDEGAVVSGPEDGCFIRGLFMEGARWDNQTHVIAESRPKELFTEMPIVWLKPEQHR 4000

Query 1432 ERSEKD-----------YECPLYKTAVRAGVLSTTGQSTNFVVAVDMPTDRDPDYWVAMG 1578

++ E+ Y+CP+YKT RAG LSTTG STNFV+ +++P+D+ +W+ G

Sbjct 4001 KKPEQPAEGDASGSIGVYDCPVYKTLTRAGTLSTTGHSTNFVMYLELPSDKPQGHWINRG 4060

Query 1579 TAMLCALA 1602

A+ LA

Sbjct 4061 VALFTGLA 4068

>ref|XP_001694409.1| UniGene infoGene info dynein heavy chain 9 [Chlamydomonas reinhardtii]

gb|EDO96110.1| Gene info dynein heavy chain 9 [Chlamydomonas reinhardtii]

Length=952

GENE ID: 5719989 CHLREDRAFT_133402 | dynein heavy chain 9

[Chlamydomonas reinhardtii] (10 or fewer PubMed links)

Score = 400 bits (1029), Expect = 5e-112

Identities = 234/537 (43%), Positives = 311/537 (57%), Gaps = 18/537 (3%)

Frame = +1

Query 1 EKIVESFSGNASIHPSFRLWLTSMPAKYFPVPVLQASVKMTFEPPKGLRANLKGTWATVT 180

E + E+ +H +FRLWLTS P+ FP+ +L+ VKMT E PKGLRA L T+ +

Sbjct 428 ELMCETQLVEGKVHRNFRLWLTSYPSPIFPISILENGVKMTNEAPKGLRAGLLRTYMSDP 487

Query 181 DKQWD---GCSKPLEWRKLLFGVAFFHAVVQERRKFGPLGWNIRYEFNGTDLEMSMETLR 351

D GCSK E+R +LFG+AFFH++VQERRKFGP+GWNI YEFN DL +S+ LR

Sbjct 488 ISNADFFTGCSKDAEFRSMLFGLAFFHSIVQERRKFGPIGWNIPYEFNENDLRISVRQLR 547

Query 352 MFLDEQEVIPWAALLYVTGQINYGGRVTDDWDRRNLMVALRRFYRPEVLEDGFPFAPAGT 531

MFLDE IP+ L Y G+ NYGG+VTD DR LM L +Y + E G+ F+ +G

Sbjct 548 MFLDEYPEIPYDTLSYTAGECNYGGKVTDSHDRHTLMTVLATYYTHTIHEPGYRFSTSG- 606

Query 532 TAASTYYSIAEGGLQAVRDWVDGLPYDDPVTVFGLHPNAKITFERQETDKLLATVTAIQP 711

TYY A + ++++GLP VFGLH NA IT + QET+ LL ++ Q

Sbjct 607 ----TYYPPAYTSYKGYMEYINGLPLISQPEVFGLHENADITKDLQETNLLLDSLMLTQS 662

Query 712 RLggggdgLSEEETVVALAEKLKAEVPELLLEENAGAGTFALNEKGELNSLQIVLLAEMG 891

R GG E E L+ P +E + NS+ VL E+G

Sbjct 663 REASGGAASFEATVGEVAGEVLERLPPNFHIE-----AVERRYPQDYYNSMNTVLAQELG 717

Query 892 RFNKLLRRVSGSLADLGKAIKGLITMSADLDAMYGAMLKNQVPLLWAKVSYPSLKPLSSW 1071

RFN LL V SL +LGKA+KGL MSA+LD + A+ +VP W K S+PSLKPL ++

Sbjct 718 RFNTLLSVVRSSLQNLGKAVKGLALMSAELDGIGRALYDGKVPAAWLKKSFPSLKPLGAY 777

Query 1072 FKDLQERVRFMRGWLAAGVSGMACYSLPAFFFPQGFMTGILQMHARRYSIPIDSLSFSYE 1251

K++ ERV F + W+ G Y + FFF Q F+TG Q +AR+ IPID + F +E

Sbjct 778 VKEVLERVAFFQSWVEDGAP--TVYWISGFFFTQAFLTGAKQNYARKCRIPIDHIDFDFE 835

Query 1252 IRteetaaacaappeDGVYIDGFFLDGARWDREKRQLADSRPTVMYDTLPVIHFVPTPHF 1431

+R + A APPEDGVY G FL+G RW + +L +S P V++ LP I VP

Sbjct 836 VR--DGAGDVDAPPEDGVYCAGLFLEGCRWSSDLHELDESEPKVLFTPLPPIWMVPREIA 893

Query 1432 ERSE-KDYECPLYKTAVRAGVLSTTGQSTNFVVAVDMPTDRDPDYWVAMGTAMLCAL 1599

+ S Y CP+YKT R GVLSTTG STNFV+ V + + +DP +W G A++ +L

Sbjct 894 KFSSFPHYLCPMYKTTERRGVLSTTGHSTNFVLDVKLASSKDPAHWTKRGVALITSL 950

Blastx GS00981 against *C. reinhardtii*. More than just the top hit is shown.

>ref|XP_001694660.1| UniGene infoGene info dynein heavy chain 2 [Chlamydomonas reinhardtii]

gb|EDP02244.1| Gene info dynein heavy chain 2 [Chlamydomonas reinhardtii]

Length=4069

GENE ID: 5720247 DHC2 | dynein heavy chain 2 [Chlamydomonas reinhardtii]

(10 or fewer PubMed links)

Score = 395 bits (1016), Expect = 9e-111

Identities = 194/339 (57%), Positives = 246/339 (72%), Gaps = 16/339 (4%)

Frame = +2

Query 11 PEVFGLHDNADITCAQNDTYAMFRTILSLQPRASSGGGKSREEVLADTAKDILDKVPEPF 190

PE+FGLH+NADITC QN+TY MF T+LSLQPR +SG G+S+E V+ A DIL ++PE F

Sbjct 3737 PEIFGLHENADITCDQNETYDMFSTVLSLQPRVASGAGQSQEAVIGALAADILGRLPELF 3796

Query 191 NLEAVSDRYPTMYEESMNTVVQQECIRYNKMLRVVLKSLKDVIKALKGEVVMTAELEAMG 370

+++AV +RYPT Y+ESMNTV+ QECIRYN +L V+ +SL + IKALKG VVM+ ELE +

Sbjct 3797 DVDAVIERYPTTYKESMNTVLTQECIRYNALLAVMKRSLGETIKALKGLVVMSPELEGVA 3856

Query 371 TSLFNNEVPQMWSKVAYPSLKPLGTWVPDLLSRLNFIQRWIDHGKPQAFWISGFYFPQAF 550

S+++N+VP++W+ AYPSLKPL WV DLL R FI W+D G P +WISGF+FPQAF

Sbjct 3857 YSMYDNQVPELWASRAYPSLKPLSAWVVDLLERCAFISGWVDKGTPPVYWISGFFFPQAF 3916

Query 551 ITGTLQNHARKHEIAIDTVSFAYKMQMHTFEGDACPSVSTPPEDGAYIYGLFIEGARWDA 730

+TGTLQN ARK+ IDTVSF +K+ EG +V + PEDG +I GLF+EGARWD

Sbjct 3917 LTGTLQNFARKYTYPIDTVSFGFKVMDALDEG----AVVSGPEDGCFIRGLFMEGARWDN 3972

Query 731 DVQLLQESEAKVLFTQLPVVHMLPEQHRKPPSE----------GVYACPIYKTLARLGTL 880

++ ES K LFT++P+V + PEQHRK P + GVY CP+YKTL R GTL

Sbjct 3973 QTHVIAESRPKELFTEMPIVWLKPEQHRKKPEQPAEGDASGSIGVYDCPVYKTLTRAGTL 4032

Query 881 STTGHSTNFVMVLDIPCSLDQPQPHWVKRGVAGLLSLNF 997

STTGHSTNFVM L++P D+PQ HW+ RGVA L F

Sbjct 4033 STTGHSTNFVMYLELPS--DKPQGHWINRGVALFTGLAF 4069

>ref|XP_001700741.1| UniGene infoGene info dynein heavy chain 6 [Chlamydomonas reinhardtii]

gb|EDP06995.1| Gene info dynein heavy chain 6 [Chlamydomonas reinhardtii]

Length=3553

GENE ID: 5726463 DHC6 | dynein heavy chain 6 [Chlamydomonas reinhardtii]

(Over 10 PubMed links)

Score = 318 bits (814), Expect = 3e-87

Identities = 164/332 (49%), Positives = 220/332 (66%), Gaps = 11/332 (3%)

Frame = +2

Query 5 AGPEVFGLHDNADITCAQNDTYAMFRTILSLQPRASSGGGKS--REEVLADTAKDILDKV 178

A PEVFGLH NADIT Q +T M ++L+ GGG R+ +LA+ A DI+ +V

Sbjct 3228 ADPEVFGLHANADITKDQQETDLMLSSLLAASSGGGGGGGAGHGRDALLAEVAADIMARV 3287

Query 179 PEPFNLEAVSDRYPTMYEESMNTVVQQECIRYNKMLRVVLKSLKDVIKALKGEVVMTAEL 358

P+PF++EAV +YP Y ESMNTV+ QE +R+N++L VV +SL + KAL+G V+M+ +L

Sbjct 3288 PQPFDIEAVRFKYPVDYFESMNTVLCQELVRFNRLLEVVHESLAGLQKALRGLVLMSGDL 3347

Query 359 EAMGTSLFNNEVPQMWSKVAYPSLKPLGTWVPDLLSRLNFIQRWIDHGKPQAFWISGFYF 538

EA+G ++++ VP++W +YPSLKPL ++V DL+ R + W++HG P FWISGFYF

Sbjct 3348 EALGNAMYDGRVPKLWMDKSYPSLKPLASYVADLIERCRMMSDWVEHGPPPVFWISGFYF 3407

Query 539 PQAFITGTLQNHARKHEIAIDTVSFAYK-MQMHTFEGDACPSVSTPPEDGAYIYGLFIEG 715

AF+TG QN+AR+ I IDT++F Y MQ H T PEDGA I G+F+EG

Sbjct 3408 THAFLTGVKQNYARRQRIPIDTITFTYTCMQGH------ADDYKTAPEDGALISGMFVEG 3461

Query 716 ARWDADVQLLQESEAKVLFTQLPVVHMLPEQHRKPPSEGVYACPIYKTLARLGTLSTTGH 895

ARWD + LQES KVLF+ P++ + P + + Y CP+Y+T R G L+TTGH

Sbjct 3462 ARWDPESCKLQESLPKVLFSPAPLIKLSPCDAAEQATFPHYECPLYRTPERRGVLATTGH 3521

Query 896 STNFVMVLDIPCSLDQPQPHWVKRGVAGLLSL 991

STNFVM L IP DQPQ HW +RGVA LLSL

Sbjct 3522 STNFVMELMIPS--DQPQDHWTRRGVAFLLSL 3551

>dbj|BAE19786.3| dynein heavy chain 9 [Chlamydomonas reinhardtii]

Length=4149

Score = 308 bits (790), Expect = 2e-84

Identities = 156/328 (47%), Positives = 215/328 (65%), Gaps = 8/328 (2%)

Frame = +2

Query 11 PEVFGLHDNADITCAQNDTYAMFRTILSLQPRASSGGGKSREEVLADTAKDILDKVPEPF 190

PEVFGLH+NADIT +T + +++ Q R +SGG S E + + A ++L+++P F

Sbjct 3829 PEVFGLHENADITKDLQETNLLLDSLMLTQSREASGGAASFEATVGEVAGEVLERLPPNF 3888

Query 191 NLEAVSDRYPTMYEESMNTVVQQECIRYNKMLRVVLKSLKDVIKALKGEVVMTAELEAMG 370

++EAV RYP Y SMNTV+ QE R+N +L VV SL+++ KA+KG +M+AEL+ +G

Sbjct 3889 DIEAVERRYPQDYYNSMNTVLAQELGRFNTLLSVVRSSLQNLGKAVKGLALMSAELDGIG 3948

Query 371 TSLFNNEVPQMWSKVAYPSLKPLGTWVPDLLSRLNFIQRWIDHGKPQAFWISGFYFPQAF 550

+L++ +VP W K ++PSLKPLG +V ++L R+ F Q W++ G P +WISGF+F QAF

Sbjct 3949 RALYDGKVPAAWLKKSFPSLKPLGAYVKEVLERVAFFQSWVEDGAPTVYWISGFFFTQAF 4008

Query 551 ITGTLQNHARKHEIAIDTVSFAYKMQMHTFEGDACPSVSTPPEDGAYIYGLFIEGARWDA 730

+TG QN+ARK I ID + F ++++ D V PPEDG Y GLF+EG RW +

Sbjct 4009 LTGAKQNYARKCRIPIDHIDFDFEVR------DGAGDVDAPPEDGVYCAGLFLEGCRWSS 4062

Query 731 DVQLLQESEAKVLFTQLPVVHMLPEQHRKPPSEGVYACPIYKTLARLGTLSTTGHSTNFV 910

D+ L ESE KVLFT LP + M+P + K S Y CP+YKT R G LSTTGHSTNF

Sbjct 4063 DLHELDESEPKVLFTPLPPIWMVPREIAKFSSFPHYLCPMYKTTERRGVLSTTGHSTNF- 4121

Query 911 MVLDIPCSLDQPQPHWVKRGVAGLLSLN 994

VLD+ + + HW KRGVA + SLN

Sbjct 4122 -VLDVKLASSKDPAHWTKRGVALITSLN 4148

>ref|XP_001694409.1| UniGene infoGene info dynein heavy chain 9 [Chlamydomonas reinhardtii]

gb|EDO96110.1| Gene info dynein heavy chain 9 [Chlamydomonas reinhardtii]

Length=952

GENE ID: 5719989 CHLREDRAFT_133402 | dynein heavy chain 9

[Chlamydomonas reinhardtii] (10 or fewer PubMed links)

Score = 308 bits (790), Expect = 2e-84

Identities = 156/328 (47%), Positives = 215/328 (65%), Gaps = 8/328 (2%)

Frame = +2

Query 11 PEVFGLHDNADITCAQNDTYAMFRTILSLQPRASSGGGKSREEVLADTAKDILDKVPEPF 190

PEVFGLH+NADIT +T + +++ Q R +SGG S E + + A ++L+++P F

Sbjct 632 PEVFGLHENADITKDLQETNLLLDSLMLTQSREASGGAASFEATVGEVAGEVLERLPPNF 691

Query 191 NLEAVSDRYPTMYEESMNTVVQQECIRYNKMLRVVLKSLKDVIKALKGEVVMTAELEAMG 370

++EAV RYP Y SMNTV+ QE R+N +L VV SL+++ KA+KG +M+AEL+ +G

Sbjct 692 HIEAVERRYPQDYYNSMNTVLAQELGRFNTLLSVVRSSLQNLGKAVKGLALMSAELDGIG 751

Query 371 TSLFNNEVPQMWSKVAYPSLKPLGTWVPDLLSRLNFIQRWIDHGKPQAFWISGFYFPQAF 550

+L++ +VP W K ++PSLKPLG +V ++L R+ F Q W++ G P +WISGF+F QAF

Sbjct 752 RALYDGKVPAAWLKKSFPSLKPLGAYVKEVLERVAFFQSWVEDGAPTVYWISGFFFTQAF 811

Query 551 ITGTLQNHARKHEIAIDTVSFAYKMQMHTFEGDACPSVSTPPEDGAYIYGLFIEGARWDA 730

+TG QN+ARK I ID + F ++++ D V PPEDG Y GLF+EG RW +

Sbjct 812 LTGAKQNYARKCRIPIDHIDFDFEVR------DGAGDVDAPPEDGVYCAGLFLEGCRWSS 865

Query 731 DVQLLQESEAKVLFTQLPVVHMLPEQHRKPPSEGVYACPIYKTLARLGTLSTTGHSTNFV 910

D+ L ESE KVLFT LP + M+P + K S Y CP+YKT R G LSTTGHSTNF

Sbjct 866 DLHELDESEPKVLFTPLPPIWMVPREIAKFSSFPHYLCPMYKTTERRGVLSTTGHSTNF- 924

Query 911 MVLDIPCSLDQPQPHWVKRGVAGLLSLN 994

VLD+ + + HW KRGVA + SLN

Sbjct 925 -VLDVKLASSKDPAHWTKRGVALITSLN 951

Blastx GS00730 against *C. reinhardtii*. More than just top hit is shown.

>ref|XP_001694660.1| UniGene infoGene info dynein heavy chain 2 [Chlamydomonas reinhardtii]

gb|EDP02244.1| Gene info dynein heavy chain 2 [Chlamydomonas reinhardtii]

Length=4069

GENE ID: 5720247 DHC2 | dynein heavy chain 2 [Chlamydomonas reinhardtii]

(10 or fewer PubMed links)

Score = 368 bits (945), Expect = 2e-102

Identities = 194/343 (56%), Positives = 242/343 (70%), Gaps = 23/343 (6%)

Frame = +1

Query 4 VFGLHANADITCAQNETQELADIMLSLQPKVSTGGGKSREEVIAEVAAGLQARDLKPFPM 183

+FGLH NADITC QNET ++ +LSLQP+V++G G+S+E VI +AA + R + F +

Sbjct 3739 IFGLHENADITCDQNETYDMFSTVLSLQPRVASGAGQSQEAVIGALAADILGRLPELFDV 3798

Query 184 DEIAARYPLSYEQSMNTVLSQECIRYNRLIRVYNKSLADLLKALKGLIVMSAELEAMATS 363

D + RYP +Y++SMNTVL+QECIRYN L+ V +SL + +KALKGL+VMS ELE +A S

Sbjct 3799 DAVIERYPTTYKESMNTVLTQECIRYNALLAVMKRSLGETIKALKGLVVMSPELEGVAYS 3858

Query 364 LYSNQVPAMWAKVAYPSLKPLAAWVDDLARRIEFLQSWDRGGPPPAYWISGFFFPQAFLT 543

+Y NQVP +WA AYPSLKPL+AWV DL R F+ W G PP YWISGFFFPQAFLT

Sbjct 3859 MYDNQVPELWASRAYPSLKPLSAWVVDLLERCAFISGWVDKGTPPVYWISGFFFPQAFLT 3918

Query 544 GTLQNYARKHKVAIDTVSFAFHVM-AQEPNSVAEAPEDGCYVFGMFLEGAVWDPDACLLA 720

GTLQN+ARK+ IDTVSF F VM A + +V PEDGC++ G+F+EGA WD ++A

Sbjct 3919 GTLQNFARKYTYPIDTVSFGFKVMDALDEGAVVSGPEDGCFIRGLFMEGARWDNQTHVIA 3978

Query 721 EARPKELYSVFPMLWLKPEVDRKPP----------TSGVYSCPLYKtttragtlsttGHS 870

E+RPKEL++ P++WLKPE RK P + GVY CP+YKT TRAGTLSTTGHS

Sbjct 3979 ESRPKELFTEMPIVWLKPEQHRKKPEQPAEGDASGSIGVYDCPVYKTLTRAGTLSTTGHS 4038

Query 871 TNFVLMIELPSDKPCSGTFSRYAETFSAHWIGRAVALFTTLTY 999

TNFV+ +ELPSDKP HWI R VALFT L +

Sbjct 4039 TNFVMYLELPSDKP------------QGHWINRGVALFTGLAF 4069

>ref|XP_001700741.1| UniGene infoGene info dynein heavy chain 6 [Chlamydomonas reinhardtii]

gb|EDP06995.1| Gene info dynein heavy chain 6 [Chlamydomonas reinhardtii]

Length=3553

GENE ID: 5726463 DHC6 | dynein heavy chain 6 [Chlamydomonas reinhardtii]

(Over 10 PubMed links)

Score = 292 bits (748), Expect = 1e-79

Identities = 157/336 (46%), Positives = 213/336 (63%), Gaps = 22/336 (6%)

Frame = +1

Query 4 VFGLHANADITCAQNETQELADIMLSLQPKVSTGGG------KSREEVIAEVAAGLQARD 165

VFGLHANADIT Q ET D+MLS S+GGG R+ ++AEVAA + AR

Sbjct 3232 VFGLHANADITKDQQET----DLMLSSLLAASSGGGGGGGAGHGRDALLAEVAADIMARV 3287

Query 166 LKPFPMDEIAARYPLSYEQSMNTVLSQECIRYNRLIRVYNKSLADLLKALKGLIVMSAEL 345

+PF ++ + +YP+ Y +SMNTVL QE +R+NRL+ V ++SLA L KAL+GL++MS +L

Sbjct 3288 PQPFDIEAVRFKYPVDYFESMNTVLCQELVRFNRLLEVVHESLAGLQKALRGLVLMSGDL 3347

Query 346 EAMATSLYSNQVPAMWAKVAYPSLKPLAAWVDDLARRIEFLQSWDRGGPPPAYWISGFFF 525

EA+ ++Y +VP +W +YPSLKPLA++V DL R + W GPPP +WISGF+F

Sbjct 3348 EALGNAMYDGRVPKLWMDKSYPSLKPLASYVADLIERCRMMSDWVEHGPPPVFWISGFYF 3407

Query 526 PQAFLTGTLQNYARKHKVAIDTVSFAFHVMAQEPNSVAEAPEDGCYVFGMFLEGAVWDPD 705

AFLTG QNYAR+ ++ IDT++F + M + APEDG + GMF+EGA WDP+

Sbjct 3408 THAFLTGVKQNYARRQRIPIDTITFTYTCMQGHADDYKTAPEDGALISGMFVEGARWDPE 3467

Query 706 ACLLAEARPKELYSVFPMLWLKPEVDRKPPTSGVYSCPLYKtttragtlsttGHSTNFVL 885

+C L E+ PK L+S P++ L P + T Y CPLY+T R G L+TTGHSTNFV+

Sbjct 3468 SCKLQESLPKVLFSPAPLIKLSPCDAAEQATFPHYECPLYRTPERRGVLATTGHSTNFVM 3527

Query 886 MIELPSDKPCSGTFSRYAETFSAHWIGRAVALFTTL 993

+ +PSD+P HW R VA +L

Sbjct 3528 ELMIPSDQP------------QDHWTRRGVAFLLSL 3551

>ref|XP_001692092.1| UniGene infoGene info dynein heavy chain 8 [Chlamydomonas reinhardtii]

gb|EDP04582.1| Gene info dynein heavy chain 8 [Chlamydomonas reinhardtii]

Length=3241

GENE ID: 5717634 DHC8 | dynein heavy chain 8 [Chlamydomonas reinhardtii]

(Over 10 PubMed links)

Score = 278 bits (711), Expect = 2e-75

Identities = 150/337 (44%), Positives = 212/337 (62%), Gaps = 19/337 (5%)

Frame = +1

Query 7 FGLHANADITCAQNETQELADIMLSLQPKVSTGGGK-------SREEVIAEVAAGLQARD 165

FGLHANADI QN+T + +LS+ GG + EE +A + + AR

Sbjct 2916 FGLHANADIAKDQNDTAAMFASLLSMTGGSGGSGGGGGGGAAGAAEERVAGIVSQCLARL 2975

Query 166 LKPFPMDEIAARYPLSYEQSMNTVLSQECIRYNRLIRVYNKSLADLLKALKGLIVMSAEL 345

+ ++ I R+P+ YE+SMNTVL+QE R+N+L+ V ++SLA++ A++GL+VMS+EL

Sbjct 2976 PPQYDIEAIQRRWPVKYEESMNTVLAQEASRFNKLLAVLHESLANIQLAIQGLLVMSSEL 3035

Query 346 EAMATSLYSNQVPAMWAKVAYPSLKPLAAWVDDLARRIEFLQSWDRGGPPPAYWISGFFF 525

E S+ NQVP +W + +YPSLKPL +++DDL R+ +W GGPPP++W+ GFFF

Sbjct 3036 EEAFASIAINQVPELWKRRSYPSLKPLGSYLDDLYERLNMFTAWAAGGPPPSFWLPGFFF 3095

Query 526 PQAFLTGTLQNYARKHKVAIDTVSFAFHVMAQEPNSVAEAPEDGCYVFGMFLEGAVWDPD 705

Q+FLT +LQNYAR+ KV IDTV F F ++ E + AP +G YV G++LEG WD

Sbjct 3096 VQSFLTASLQNYARRRKVPIDTVGFGFEMLGMEHAAYKTAPAEGVYVHGLYLEGCGWDAG 3155

Query 706 ACLLAEARPKELYSVFPMLWLKPEVDRKPPTSGVYSCPLYKtttragtlsttGHSTNFVL 885

L E++PK L+ P++WL+P+ K Y CPLY+T R G L+TTGHSTNFV+

Sbjct 3156 PQRLCESQPKVLFVNAPVMWLRPQSADKKFDYPHYDCPLYRTADRRGVLATTGHSTNFVM 3215

Query 886 MIELPSDKPCSGTFSRYAETFSAHWIGRAVALFTTLT 996

++LP+D+P S HWI R VAL T L+

Sbjct 3216 FVKLPTDQPAS------------HWIMRGVALLTQLS 3240

Conclusion: GS00012, GS00981, and GS00730 hit to overlapping regions of the same *C. reinhardtiii* proteins (dynein heavy chain 2).

Clustal alignment of the three DHC2 homologs against *C. reinhardtii* DHC2:

CLUSTAL W (1.83) multiple sequence alignment

GS00981 ------------------------------------------------------------

GS00730 ------------------------------------------------------------

DHC2_CHLRE MAPFETRSGETPRKVLIQRRRRQFAAQDVAELVHGEGVAQPPQELFPLEVFDNTNFESRM

GS00012 ------------------------------------------------------------

GS00981 ------------------------------------------------------------

GS00730 ------------------------------------------------------------

DHC2_CHLRE HPEWSLHGERPQTPTTKPAGVPLTSGRALVAHDDGTGHSVVDWVPCTVVDFDEATNSYGV

GS00012 ------------------------------------------------------------

GS00981 ------------------------------------------------------------

GS00730 ------------------------------------------------------------

DHC2_CHLRE TLHQLAHSGNGSAEADAEDMHVMWLPRVKVCFSAEDPAQFARRHAEAHRSRARAESLLRY

GS00012 ------------------------------------------------------------

GS00981 ------------------------------------------------------------

GS00730 ------------------------------------------------------------

DHC2_CHLRE NLYVDSMPTDDIPPLTNEQVNRMLSFALNSKKLKDKLMDTSALIAEVNIEYARTMNKVVF

GS00012 ------------------------------------------------------------

GS00981 ------------------------------------------------------------

GS00730 ------------------------------------------------------------

DHC2_CHLRE DTALTAARAEREADAEAAANGGSDFPRDPPRPVPERGTVPVEGGADFPQQFSEFSFKTLL

GS00012 ------------------------------------------------------------

GS00981 ------------------------------------------------------------

GS00730 ------------------------------------------------------------

DHC2_CHLRE TKTEVIMAITKIKVECAKVTKMCLFNTHYTKSARLEELEQTQVAALDSAGNYLKDTWCVA

GS00012 ------------------------------------------------------------

GS00981 ------------------------------------------------------------

GS00730 ------------------------------------------------------------

DHC2_CHLRE LRNAIRNSFKDVGKGWFNLGEASMETYEFSKLRKFLTLTRFVMEDTMRALVEDSLGKFTG

GS00012 ------------------------------------------------------------

GS00981 ------------------------------------------------------------

GS00730 ------------------------------------------------------------

DHC2_CHLRE FIQSCCPGRVTVHSTSSVEILDASSPVPVPAIGPGRKPPLLVMDLATNKEATRFVYSTQP

GS00012 ------------------------------------------------------------

GS00981 ------------------------------------------------------------

GS00730 ------------------------------------------------------------

DHC2_CHLRE ESIVTKIMALFDAAIGRTQGLHTLEPAIMENLFWATAPVLSTVHQQEEIVVRHRELLRAA

GS00012 ------------------------------------------------------------

GS00981 ------------------------------------------------------------

GS00730 ------------------------------------------------------------

DHC2_CHLRE LSAALVPLEEYMAKFEKYVPLLQLNVESYVAALEAKGEELSLTEVRAEIKRASADLESLM

GS00012 ------------------------------------------------------------

GS00981 ------------------------------------------------------------

GS00730 ------------------------------------------------------------

DHC2_CHLRE ESVPIGISLGLVQINLVKTRELLVKKQEKLVALLKALAARVPRRAMASVSTKFAEIDRAL

GS00012 ------------------------------------------------------------

GS00981 ------------------------------------------------------------

GS00730 ------------------------------------------------------------

DHC2_CHLRE KAKANNLEDVDEQRHYIESLPNKVYELMADVEAQRGWYELLEGMRYLLPEEDLKEKFAGE

GS00012 ------------------------------------------------------------

GS00981 ------------------------------------------------------------

GS00730 ------------------------------------------------------------

DHC2_CHLRE SWGMRLTRQAEKQLEVLAGDEARFKGEMITEQDMFRDTIGDLQVLVSNFGLYTDLGKMEA

GS00012 ------------------------------------------------------------

GS00981 ------------------------------------------------------------

GS00730 ------------------------------------------------------------

DHC2_CHLRE VVTEVRSVDERLKKADRDAGVYNSREALLGLPPTDYSPLKKVIDTFEPFLQFWTTASNWR

GS00012 ------------------------------------------------------------

GS00981 ------------------------------------------------------------

GS00730 ------------------------------------------------------------

DHC2_CHLRE SLHKSWMHDSWEKLHGETVEREVTNAYKVLFKTAKVFGQRGGLDKCAENCELIREEVEAF

GS00012 ------------------------------------------------------------

GS00981 ------------------------------------------------------------

GS00730 ------------------------------------------------------------

DHC2_CHLRE KKFVPLVQALRNPGMRDRHWDQLSALLGFKLHPDKTFTMAAAEQMGLLQHLQAITKVADV

GS00012 ------------------------------------------------------------

GS00981 ------------------------------------------------------------

GS00730 ------------------------------------------------------------

DHC2_CHLRE AGKEYSIEQALDKMQREWESAEMQVLDYRETKTFVIKVEEQISQMLDDHIAMTQSMAFSP

GS00012 ------------------------------------------------------------

GS00981 ------------------------------------------------------------

GS00730 ------------------------------------------------------------

DHC2_CHLRE YKKPFEERIAKWEQQLSLVSEILDQWIQLQRQWMYLEPIFGSEDIMQQLPLEGKRFATVD

GS00012 ------------------------------------------------------------

GS00981 ------------------------------------------------------------

GS00730 ------------------------------------------------------------

DHC2_CHLRE RMWRKTTDAAKRNPLLLKVCSSQKLLDSFIEANKLLESVQKGLADYLETKRLAFARFFFL

GS00012 ------------------------------------------------------------

GS00981 ------------------------------------------------------------

GS00730 ------------------------------------------------------------

DHC2_CHLRE SNDELLQILSQTKNPLAVQPHLRKCFEAIESLDFAPNGEIGAMNSREKEKVPFDKPMMPQ

GS00012 ------------------------------------------------------------

GS00981 ------------------------------------------------------------

GS00730 ------------------------------------------------------------

DHC2_CHLRE GNVEIWLGEVERRMRFSVRHQVVLAVAAYATTPRKQWVRDWPAMVVLAVSAIYWSREVEE

GS00012 ------------------------------------------------------------

GS00981 ------------------------------------------------------------

GS00730 ------------------------------------------------------------

DHC2_CHLRE AISEGSVPAYLDKCSADLLDLTDLVRGRLSGQERLTLGALITIDVHARDVVAELAEAKIK

GS00012 ------------------------------------------------------------

GS00981 ------------------------------------------------------------

GS00730 ------------------------------------------------------------

DHC2_CHLRE NPTDFEWVSRLRYYWRNDDVCVDMVQASIAYGYEYLGNTPRLVITPLTDRCYMTLMSAMH

GS00012 ------------------------------------------------------------

GS00981 ------------------------------------------------------------

GS00730 ------------------------------------------------------------

DHC2_CHLRE MNLGGAPAGPAGTGKTETTKDLAKALAKQCVVFNCSDGLDYQAMAKFFKGLASSGAWACF

GS00012 ------------------------------------------------------------

GS00981 ------------------------------------------------------------

GS00730 ------------------------------------------------------------

DHC2_CHLRE DEFNRIDLEVLSVVAQQILTIQLAIQAKVKRFIFEDTEIDLNPACSVYITMNPGYAGRSE

GS00012 ------------------------------------------------------------

GS00981 ------------------------------------------------------------

GS00730 ------------------------------------------------------------

DHC2_CHLRE LPDNLKALFRPCAMMVPDYALIAEICLYSYGYKNGKDLARKMVATFKLCSEQLSSQDHYD

GS00012 ------------------------------------------------------------

GS00981 ------------------------------------------------------------

GS00730 ------------------------------------------------------------

DHC2_CHLRE YGMRAVKSVITAAGNLKREFPDDDEEVLLLRALRDVNVPKFLSHDLPLFDGIITDLFPGV

GS00012 ------------------------------------------------------------

GS00981 ------------------------------------------------------------

GS00730 ------------------------------------------------------------

DHC2_CHLRE KMPEVDYNSLLKALDESCGELGIQPVESFVGKVIQLYETTIVRHGLMLVGPTMGGKTCCY

GS00012 ------------------------------------------------------------

GS00981 ------------------------------------------------------------

GS00730 ------------------------------------------------------------

DHC2_CHLRE RSLQKAMTKLAAAGDSKYERVRVVALNPKSITMGQLYGEFDENTHEWTDGVLACYMRECS

GS00012 ------------------------------------------------------------

GS00981 ------------------------------------------------------------

GS00730 ------------------------------------------------------------

DHC2_CHLRE EDTKPDKKWIMFDGPVDAVWIENMNTVLDDNKKLCLVSGEIIQLSASMTMMFEVEDLAVA

GS00012 ------------------------------------------------------------

GS00981 ------------------------------------------------------------

GS00730 ------------------------------------------------------------

DHC2_CHLRE SPATVSRCGMVYMEPTALGLEPLLTSWLARLPPGGVAENSGKLGAIFNALVPDALRFLRK

GS00012 ------------------------------------------------------------

GS00981 ------------------------------------------------------------

GS00730 ------------------------------------------------------------

DHC2_CHLRE NLKETVTTVNNNLVASCFGLMDSLTKPFVRGEGEDPLTADEKVKLGGFLPSLMLFSIVWS

GS00012 ------------------------------------------------------------

GS00981 ------------------------------------------------------------

GS00730 ------------------------------------------------------------

DHC2_CHLRE LGASCDKAGRTLFDEWFRKHAAECGLPLEGAMFPGEGTVYDWVYDTDGTFAEGGAPGWVG

GS00012 ------------------------------------------------------------

GS00981 ------------------------------------------------------------

GS00730 ------------------------------------------------------------

DHC2_CHLRE WMATVPEFKCDPDRPFSEIIVPTADTVRYTYVVDKLVANQRHVLCVGETGTGKTLNVSNK

GS00012 ------------------------------------------------------------

GS00981 ------------------------------------------------------------

GS00730 ------------------------------------------------------------

DHC2_CHLRE LLNDMPPEVQPVFMTFSARTSANQTQDIIDAKMDKRRKGVFGPPAGKRMVIFIDDLNMPQ

GS00012 ------------------------------------------------------------

GS00981 ------------------------------------------------------------

GS00730 ------------------------------------------------------------

DHC2_CHLRE REKYFAQPPIELLRQWMDHGGWYERKPPCPFRTIVDTQFVAAMGPPGGGRNPVTNRLLRH

GS00012 ------------------------------------------------------------

GS00981 ------------------------------------------------------------

GS00730 ------------------------------------------------------------

DHC2_CHLRE FNFISFTEMSDSSVSRIFTTILGAFFRKYFGDAIQALTDPVVTATVRLYNSIRAELLPTP

GS00012 ------------------------------------------------------------

GS00981 ------------------------------------------------------------

GS00730 ------------------------------------------------------------

DHC2_CHLRE TRSHYTFNLRDLSKVVQGVMRADPRSTGDSKQVLSLWLHECSRVFEDRLINDEDHGWFRA

GS00012 ------------------------------------------------------------

GS00981 ------------------------------------------------------------

GS00730 ------------------------------------------------------------

DHC2_CHLRE RQEALLTENFGLGYGDVVTSERLIFGDFMVPGADPRVYSQITDMPKLVKVVEEYLEDYNS

GS00012 ------------------------------------------------------------

GS00981 ------------------------------------------------------------

GS00730 ------------------------------------------------------------

DHC2_CHLRE VSSAPMKLVMFLDAIEHVSRITRVIRLPLGNALLLGVGGSGRQSLTRLAAYMEEYDVVQI

GS00012 ------------------------------------------------------------

GS00981 ------------------------------------------------------------

GS00730 ------------------------------------------------------------

DHC2_CHLRE EIAKGYGSNEWRDDLRKVLRKTGLDGRDTVFLFTDTQIVQENFLEDINNILNSGEVPNLW

GS00012 ------------------------------------------------------------

GS00981 ------------------------------------------------------------

GS00730 ------------------------------------------------------------

DHC2_CHLRE GNDDQEAIANAMRPLMAAAGLPITKMGISTFFINRVRSYLHVVLCFSPIGDAFRQRLRMF

GS00012 ------------------------------------------------------------

GS00981 ------------------------------------------------------------

GS00730 ------------------------------------------------------------

DHC2_CHLRE PSLVNCCTIDWFREWPEEALRSVADSFYGDVDFGDDTGAIMAGVVDCCVGVHQSVEKKSK

GS00012 ------------------------------------------------------------

GS00981 ------------------------------------------------------------

GS00730 ------------------------------------------------------------

DHC2_CHLRE KFYDELRRYNYVTPTSYLELLTTFIKLLGEKRTEIAEKRRRLEVGLQKLLNTAGQVEVMQ

GS00012 ------------------------------------------------------------

GS00981 ------------------------------------------------------------

GS00730 ------------------------------------------------------------

DHC2_CHLRE KELQELQPVLAATAKEVEDMMVVITNDKKEADETKKQVEQQEKDANEQAARAKQIAEDAQ

GS00012 ------------------------------------------------------------

GS00981 ------------------------------------------------------------

GS00730 ------------------------------------------------------------

DHC2_CHLRE RDLDEALPALERALESLKNLSRNDIVEVKSLQNPPAGVRTVMDATCIMFDEKPKMKDDPA

GS00012 ------------------------------------------------------------

GS00981 ------------------------------------------------------------

GS00730 ------------------------------------------------------------

DHC2_CHLRE NVGKKVPDYWEPAKKLLNDPTKFLESLFSYDKDNIPDHVIKKIEPYIQRDDFTPEAISKV

GS00012 ------------------------------------------------------------

GS00981 ------------------------------------------------------------

GS00730 ------------------------------------------------------------

DHC2_CHLRE SKACTSICMWVRAMYVYHNVALSVAPKRAALAAAQEQLNETMEQLRAAQAKLKAVEEKIA

GS00012 ------------------------------------------------------------

GS00981 ------------------------------------------------------------

GS00730 ------------------------------------------------------------

DHC2_CHLRE TLEAQYEEALAKKAQLAQQVLRCTVQLQRADKLIGGLGGERVRWQATVDQLADDLINVVG

GS00012 ------------------------------------------------------------

GS00981 ------------------------------------------------------------

GS00730 ------------------------------------------------------------

DHC2_CHLRE DVVISAATIAYSGPFTPLYRSSLVHEWSGFLEAAKVPATKGTNLLSTLQDPVKVRAWTIA

GS00012 ------------------------------------------------------------

GS00981 ------------------------------------------------------------

GS00730 ------------------------------------------------------------

DHC2_CHLRE GLPTDTLSVENGIIVSKARRWPLMIDPQGQANKWIKNMERESGLDVIKLSDKDFLRTLEN

GS00012 ------------------------------------------------------------

GS00981 ------------------------------------------------------------

GS00730 ------------------------------------------------------------

DHC2_CHLRE GVRFGRAVLLENIGETLDAALEPLLLKQTFKQGGSEVIKIGDNIIPYHPDFRFYMTTKLR

GS00012 ------------------------------------------------------------

GS00981 ------------------------------------------------------------

GS00730 ------------------------------------------------------------

DHC2_CHLRE NPHYAPEVSVKVSLLNFFVTPEGLEDQLLGTVVTQERPDLANLKSQLVVSNAKMKKELSD

GS00012 ------------------------------------------------------------

GS00981 ------------------------------------------------------------

GS00730 ------------------------------------------------------------

DHC2_CHLRE IEDRILQLLSASSGEILDDEELINTLAQSKVTSNEISAKVAEAEATEREIDETRELYRPV

GS00012 ------------------------------------------------------------

GS00981 ------------------------------------------------------------

GS00730 ------------------------------------------------------------

DHC2_CHLRE ALRASLLFFAISDLALVDPMYQYSLAWFISLFIRGIEEAPKAASVEERGHNLNEYFTYSL

GS00012 ------------------------------------------------------------

GS00981 ------------------------------------------------------------

GS00730 ------------------------------------------------------------

DHC2_CHLRE YVNICRSLFEAHKLMFSLLLTIKILQNRNMIDGREWRFLLAGPTTSELSQPNPAPDWLTD

GS00012 ------------------------------------------------------------

GS00981 ------------------------------------------------------------

GS00730 ------------------------------------------------------------

DHC2_CHLRE KAWNELLNLSHLPTFKGFADHVAANLPHYRAIFDSNDAHELPLAPPWEDKLDTFQKLSFL

GS00012 ------------------------------------------------------------

GS00981 ------------------------------------------------------------

GS00730 ------------------------------------------------------------

DHC2_CHLRE RCLRPDKVTGAVQAFVSQHLGQRFIEPPPFDLATCYKESSPSVPLIFVLSPGADPMADLL

GS00012 ------------------------------------------------------------

GS00981 ------------------------------------------------------------

GS00730 ------------------------------------------------------------

DHC2_CHLRE KLAEDMKFSRKFEKVSLGQGQGPKAEKLLEAGMERGIWVCLQNCHLAVSWMPTLERIVEG

GS00012 ------------------------------------------------------EKIVES

GS00981 ------------------------------------------------------------

GS00730 ------------------------------------------------------------

DHC2_CHLRE IQPD-RVHKDFRLWLTSMPSPDFPVAILQNGVKMTLEPPKGLKSNLVRQYNRLTDAYLAA

GS00012 FSGNASIHPSFRLWLTSMPAKYFPVPVLQASVKMTFEPPKGLRANLKGTWATVTDKQWDG

GS00981 ------------------------------------------------------------

GS00730 ------------------------------------------------------------

DHC2_CHLRE SSKPEDWRRLVFGLCLFHAVIQDRRKFGPLGWNIRYDFTDGDLNVSLAQLQEYLDKYEVI

GS00012 CSKPLEWRKLLFGVAFFHAVVQERRKFGPLGWNIRYEFNGTDLEMSMETLRMFLDEQEVI

GS00981 ------------------------------------------------------------

GS00730 ------------------------------------------------------------

DHC2_CHLRE PFKVLRFLFTEINYGGRVTDDKDRRLINNLIYTFCGPSVLEPGYAFSPSGTYATPPEAVV

GS00012 PWAALLYVTGQINYGGRVTDDWDRRNLMVALRRFYRPEVLEDGFPFAPAGTTAASTYYSI

GS00981 -----------------NAGPEVFGLHDNADITCAQNDTYAMFRTILSLQPRASSGG-GK

GS00730 ---------------------NVFGLHANADITCAQNETQELADIMLSLQPKVSTGG-GK

DHC2_CHLRE S---VRDHLELLRAYPIVPKPEIFGLHENADITCDQNETYDMFSTVLSLQPRVASGA-GQ

GS00012 AEGGLQAVRDWVDGLPYDDPVTVFGLHPNAKITFERQETDKLLATVTAIQPRLGGGGDGL

:**** **.** :::* : : ::**: . *. *

GS00981 SREEVLADTAKDILDKVPEPF-NLEAVSDRYPTMYEESMNT---VVQQECIRYNKMLRVV

GS00730 SREEVIAEVAAGLQARDLKPF-PMDEIAARYPLSYEQSMNT---VLSQECIRYNRLIRVY

DHC2_CHLRE SQEAVIGALAADILGRLPELF-DVDAVIERYPTTYKESMNT---VLTQECIRYNALLAVM

GS00012 SEEETVVALAEKLKAEVPELLLEENAGAGTFALNEKGELNSLQIVLLAEMGRFNKLLRRV

*.* .: * : . : : : :. : .:*: *: * *:* ::

GS00981 LKSLKDVIKALKGEVVMTAELEAMGTSLFNNEVPQMWSKVAYPSLKPLGTWVPDLLSRLN

GS00730 NKSLADLLKALKGLIVMSAELEAMATSLYSNQVPAMWAKVAYPSLKPLAAWVDDLARRIE

DHC2_CHLRE KRSLGETIKALKGLVVMSPELEGVAYSMYDNQVPELWASRAYPSLKPLSAWVVDLLERCA

GS00012 SGSLADLGKAIKGLITMSADLDAMYGAMLKNQVPLLWAKVSYPSLKPLSSWFKDLQERVR

** : **:** :.*:.:*:.: :: .*:** :*:. :*******.:*. ** *

GS00981 FIQRWIDHGKP--QAFWISGFYFPQAFITGTLQNHARKHEIAIDTVSFAYKMQMHTFEGD

GS00730 FLQSWDRGGPP--PAYWISGFFFPQAFLTGTLQNYARKHKVAIDTVSFAF----HVMAQE

DHC2_CHLRE FISGWVDKGTP--PVYWISGFFFPQAFLTGTLQNFARKYTYPIDTVSFGF----KVMDAL

GS00012 FMRGWLAAGVSGMACYSLPAFFFPQGFMTGILQMHARRYSIPIDSLSFSY----EIRTEE

*: * * . : :..*:***.*:** ** .**:: .**::**.: .

GS00981 ACPSVSTPPEDGAYIYGLFIEGARWDADVQLLQESEAKVLFTQLPVVHMLPEQHRKPP--

GS00730 P-NSVAEAPEDGCYVFGMFLEGAVWDPDACLLAEARPKELYSVFPMLWLKPEVDRKPP--

DHC2_CHLRE DEGAVVSGPEDGCFIRGLFMEGARWDNQTHVIAESRPKELFTEMPIVWLKPEQHRKKPEQ

GS00012 TAAACAAPPEDGVYIDGFFLDGARWDREKRQLADSRPTVMYDTLPVIHFVPTPHFERS--

: **** :: *:*::** ** : : ::... :: :*:: : * . : .

GS00981 --------SEGVYACPIYKTLARLGTLSTTGHSTNFVMVLDIP----CS------LDQPQ

GS00730 --------TSGVYSCPLYKTTTRAGTLSTTGHSTNFVLMIELPSDKPCSGTFSRYAETFS

DHC2_CHLRE PAEGDASGSIGVYDCPVYKTLTRAGTLSTTGHSTNFVMYLELP------------SDKPQ

GS00012 ---------EKDYECPLYKTAVRAGVLSTTGQSTNFVVAVDMP------------TDRDP

* **:*** .* *.*****:*****: :::* :

GS00981 PHWVKRGVAGLLSLNF-----------------------------

GS00730 AHWIGRAVALFTTLTY-KQWSVRGVCLQRVS-TRFVNYRGADDLF

DHC2_CHLRE GHWINRGVALFTGLAF-----------------------------

GS00012 DYWVAMGTAMLCALAS-----------------------------

:*: ..* : *

Conclustion: GS00981, GS00730, and GS00012 appear to be distinct based alignment with closely overlapping region of *C. reinhardtii* DHC2 but distinct amino acid sequences.

Blastx GS11376 against *C. reinhardtii*

>ref|XP_001700741.1| UniGene infoGene info dynein heavy chain 6 [Chlamydomonas reinhardtii]

gb|EDP06995.1| Gene info dynein heavy chain 6 [Chlamydomonas reinhardtii]

Length=3553

GENE ID: 5726463 DHC6 | dynein heavy chain 6 [Chlamydomonas reinhardtii]

(Over 10 PubMed links)

Score = 44.3 bits (103), Expect = 2e-05

Identities = 27/76 (35%), Positives = 34/76 (44%), Gaps = 20/76 (26%)

Frame = +2

Query 5 WASQLRCRWEEEAEVESRSLRGRAADLKGDGGNLHVTIGEYNRRHGYEYASGGEVLVVTP 184

W SQLR WE GG + V + +GYEY G LV+TP

Sbjct 790 WNSQLRYGWE--------------------GGEVSVRMINAQIYYGYEYLGNGSRLVITP 829

Query 185 LTERSYLSLARAAHLS 232

LT+R Y +L A HL+

Sbjct 830 LTDRCYRTLMGALHLN 845

Blastx e00124.1 against *C. reinhardtii*

>dbj|BAE19786.3| dynein heavy chain 9 [Chlamydomonas reinhardtii]

Length=4149

Score = 467 bits (1201), Expect = 5e-132

Identities = 237/432 (54%), Positives = 307/432 (71%), Gaps = 7/432 (1%)

Frame = +3

Query 3 GPLGWNIPYEFNESDLRISVRQLQMFLDSYDETPLAALNYLTAECNYGGRVTDDKDRRTL 182

GP+GWNIPYEFNE+DLRISVRQL+MFLD Y E P L+Y ECNYGG+VTD DR TL

Sbjct 3721 GPIGWNIPYEFNENDLRISVRQLRMFLDEYPEIPYDTLSYTAGECNYGGKVTDSHDRHTL 3780

Query 183 VTAVLNIYCPQILEDGYRLSASGMYVVPTDALDSHEATVEYIRQWPPVAKPEVFGLHENA 362

+T + Y I E GYR S SG Y P A S++ +EYI P +++PEVFGLHENA

Sbjct 3781 MTVLATYYTHTIHEPGYRFSTSGTYYPP--AYTSYKGYMEYINGLPLISQPEVFGLHENA 3838

Query 363 DITKDLGEVDLMLKTMLLTQAQSggggggggKSTDDVVAEISGEILGKLPSNFDMEVAAR 542

DITKDL E +L+L +++LTQ++ GG ++T V E++GE+L +LP NFD+E R

Sbjct 3839 DITKDLQETNLLLDSLMLTQSREASGGAASFEAT---VGEVAGEVLERLPPNFDIEAVER 3895

Query 543 RYPVVYSESMNTVLRQELQRFNKLTSKIRSSLLELGKALKGLVVMSADLESIASAMFVNQ 722

RYP Y SMNTVL QEL RFN L S +RSSL LGKA+KGL +MSA+L+ I A++ +

Sbjct 3896 RYPQDYYNSMNTVLAQELGRFNTLLSVVRSSLQNLGKAVKGLALMSAELDGIGRALYDGK 3955

Query 723 VPESWAKVSYPSLKPLSAYVTELLERLEFFSTWLEEGNPVCYPMPHFFFVQAFMTGALQN 902

VP +W K S+PSLKPL AYV E+LER+ FF +W+E+G P Y + FFF QAF+TGA QN

Sbjct 3956 VPAAWLKKSFPSLKPLGAYVKEVLERVAFFQSWVEDGAPTVYWISGFFFTQAFLTGAKQN 4015

Query 903 YARKYTLPIDTVEFDFHFFWEA--PTAKPEDGVHTSGLFVEGARMGDEKVQLEESQPKVL 1076

YARK +PID ++FDF A A PEDGV+ +GLF+EG R + +L+ES+PKVL

Sbjct 4016 YARKCRIPIDHIDFDFEVRDGAGDVDAPPEDGVYCAGLFLEGCRWSSDLHELDESEPKVL 4075

Query 1077 FSPMTYVQLLPVQSDKLSTYPHYECPVYRTTARRGTLSTTGHSTNFVMFMRLPTDQPSSH 1256

F+P+ + ++P + K S++PHY CP+Y+TT RRG LSTTGHSTNFV+ ++L + + +H

Sbjct 4076 FTPLPPIWMVPREIAKFSSFPHYLCPMYKTTERRGVLSTTGHSTNFVLDVKLASSKDPAH 4135

Query 1257 WIARGVALINSL 1292

W RGVALI SL

Sbjct 4136 WTKRGVALITSL 4147

Blastx e00124.2 against *C. reinhardtii*

>ref|XP_690143.3| UniGene infoGene info PREDICTED: similar to Dynein heavy chain 7, axonemal (Ciliary

dynein heavy chain 7) (Axonemal beta dynein heavy chain 7)

(Dynein heavy chain-like protein 2) (HDHC2) [Danio rerio]

Length=3990

GENE ID: 561639 LOC561639 | similar to Dynein heavy chain 7, axonemal (Ciliary

dynein heavy chain 7) (Axonemal beta dynein heavy chain 7) (Dynein heavy

chain-like protein 2) (HDHC2) [Danio rerio]

Score = 195 bits (496), Expect = 3e-48

Identities = 99/211 (46%), Positives = 134/211 (63%), Gaps = 3/211 (1%)

Frame = +1

Query 10 FGADYMRMATSVVGATIDAYKESIANLLPTPSKSHYTFNLRDVARVVQGLLLTNVEDYTG 189

F + + + ++ AT+ Y+E+ NLLPTP+KSHY FNLRD +RV+QG+ L+ E

Sbjct 2107 FPKPFASVTSQIISATMSVYQEATKNLLPTPAKSHYLFNLRDFSRVIQGICLSRPETADD 2166

Query 190 PSDMIMAWCHELNRVFYDRLTDMDDCAWFREMLVGLCTKHFQQPVSKLFEERGLPLGSTG 369

+ + W HE+ RV+YDRL DDCAW L + H + +LF+ L S G

Sbjct 2167 LTAIKRLWVHEVLRVYYDRLVHPDDCAWMVGFLQEVSKSHLNEDFHQLFKH--LDSNSDG 2224

Query 370 DVDDDSLRRLIYCNFAVPKGK-RTYRRVESTEGLQSTMSTFLDDYNAVSSKPMKLVLFLF 546

V +D LR L++C+F PKG+ R YR V E L+ + T L++YN +S PM LVLF F

Sbjct 2225 LVTEDDLRSLMFCDFHDPKGEDRNYREVGDAEKLRQVVETHLEEYNNISKAPMNLVLFRF 2284

Query 547 AMEHVCRISRVLQMPRGNVLLAGVGGSGRQS 639

A+EHVCRISR+L+ PRG+ LL GVGGSGRQS

Sbjct 2285 AIEHVCRISRILKQPRGHALLVGVGGSGRQS 2315

Score = 46.6 bits (109), Expect = 0.002

Identities = 21/35 (60%), Positives = 25/35 (71%), Gaps = 0/35 (0%)

Frame = +3

Query 630 STVGHSTNFVMFMPLPTDQPSSHWIARGVALINSL 734

ST GHSTN+V+ M L +D P HWI RGVAL+ L

Sbjct 3954 STTGHSTNYVISMALNSDVPPEHWIRRGVALLCQL 3988

Blastx e01392.1 against *C. reinhardtii*

>dbj|BAE19786.3| dynein heavy chain 9 [Chlamydomonas reinhardtii]

Length=4149

Score = 256 bits (653), Expect(2) = 1e-81

Identities = 123/232 (53%), Positives = 159/232 (68%), Gaps = 0/232 (0%)

Frame = +3

Query 12 EDVLDKIAEGIASKLPPAFDLDVAGANYPVQYLESMNTVLHQELIRFNRLTSVIRSSLAS 191

E + ++A + +LPP FD++ YP Y SMNTVL QEL RFN L SV+RSSL +

Sbjct 3870 EATVGEVAGEVLERLPPNFDIEAVERRYPQDYYNSMNTVLAQELGRFNTLLSVVRSSLQN 3929

Query 192 LRKAIRGLVVMSSDLEALGTALVQGVRPALWMKRSFPSLKPLGSYVSDLLARLAFFQGWL 371

L KA++GL +MS++L+ +G AL G PA W+K+SFPSLKPLG+YV ++L R+AFFQ W+

Sbjct 3930 LGKAVKGLALMSAELDGIGRALYDGKVPAAWLKKSFPSLKPLGAYVKEVLERVAFFQSWV 3989

Query 372 DNGVPTHFWISGFFFTQAFLTGSSQNYARANAIPIDHLGFDMHVLPANHDCSVAPQEGVY 551

++G PT +WISGFFFTQAFLTG+ QNYAR IPIDH+ FD V D P++GVY

Sbjct 3990 EDGAPTVYWISGFFFTQAFLTGAKQNYARKCRIPIDHIDFDFEVRDGAGDVDAPPEDGVY 4049

Query 552 VHGIFLEGARFDESSAVLGESEPKVLFTKLPSLWLRPQREADIADRAHYLXP 707

G+FLEG R+ L ESEPKVLFT LP +W+ P+ A + HYL P

Sbjct 4050 CAGLFLEGCRWSSDLHELDESEPKVLFTPLPPIWMVPREIAKFSSFPHYLCP 4101

Score = 65.5 bits (158), Expect(2) = 1e-81

Identities = 30/52 (57%), Positives = 38/52 (73%), Gaps = 2/52 (3%)

Frame = +1

Query 703 CPLYKTSDRRGTLSTTGHSTNFVMFLKLPRLEEQPQEHWVKRGVAALCELDD 858

CP+YKT++RRG LSTTGHSTNFV+ +KL + HW KRGVA + L+D

Sbjct 4100 CPMYKTTERRGVLSTTGHSTNFVLDVKL--ASSKDPAHWTKRGVALITSLND 4149

Blastx e01392.2 against *C. reinhardtii*

>dbj|BAE19786.3| dynein heavy chain 9 [Chlamydomonas reinhardtii]

Length=4149

Score = 212 bits (539), Expect = 9e-56

Identities = 105/203 (51%), Positives = 135/203 (66%), Gaps = 0/203 (0%)

Frame = +2

Query 2 QERRKFGALGWNIPYEFNESDLRISVRQLRLFLTMFDDVPFEALRYCFGEANYGGRVTDD 181

QERRKFG +GWNIPYEFNE+DLRISVRQLR+FL + ++P++ L Y GE NYGG+VTD

Sbjct 3715 QERRKFGPIGWNIPYEFNENDLRISVRQLRMFLDEYPEIPYDTLSYTAGECNYGGKVTDS 3774

Query 182 KDRRCLSALLAEPISAAAIEPGFTFSSDGVYCQPGAATHEGYLESIRALPLTQLPTAFGL 361

DR L +LA + EPG+ FS+ G Y P +++GY+E I LPL P FGL

Sbjct 3775 HDRHTLMTVLATYYTHTIHEPGYRFSTSGTYYPPAYTSYKGYMEYINGLPLISQPEVFGL 3834

Query 362 HENADITKDLKETRELFDAVLSTQARagaggggggEDVLDKIAEGIASKLPPAFDLDVAG 541

HENADITKDL+ET L D+++ TQ+R +GG E + ++A + +LPP FD++

Sbjct 3835 HENADITKDLQETNLLLDSLMLTQSREASGGAASFEATVGEVAGEVLERLPPNFDIEAVE 3894

Query 542 ANYPVQYLESMNTVLHQELIHFN 610

YP Y SMNTVL QEL FN

Sbjct 3895 RRYPQDYYNSMNTVLAQELGRFN 3917

Blastx GS01392 consensus againt *C. reinhardtii*

>dbj|BAE19786.3| dynein heavy chain 9 [Chlamydomonas reinhardtii]

Length=4149

Score = 421 bits (1081), Expect(2) = 7e-130

Identities = 206/387 (53%), Positives = 265/387 (68%), Gaps = 0/387 (0%)

Frame = +2

Query 2 QERRKFGALGWNIPYEFNESDLRISVRQLRLFLTMFDDVPFEALRYCFGEANYGGRVTDD 181

QERRKFG +GWNIPYEFNE+DLRISVRQLR+FL + ++P++ L Y GE NYGG+VTD

Sbjct 3715 QERRKFGPIGWNIPYEFNENDLRISVRQLRMFLDEYPEIPYDTLSYTAGECNYGGKVTDS 3774

Query 182 KDRRCLSALLAEPISAAAIEPGFTFSSDGVYCQPGAATHEGYLESIRALPLTQLPTAFGL 361

DR L +LA + EPG+ FS+ G Y P +++GY+E I LPL P FGL

Sbjct 3775 HDRHTLMTVLATYYTHTIHEPGYRFSTSGTYYPPAYTSYKGYMEYINGLPLISQPEVFGL 3834

Query 362 HENADITKDLKETRELFDAVLSTQARagaggggggEDVLDKIAEGIASKLPPAFDLDVAG 541

HENADITKDL+ET L D+++ TQ+R +GG E + ++A + +LPP FD++

Sbjct 3835 HENADITKDLQETNLLLDSLMLTQSREASGGAASFEATVGEVAGEVLERLPPNFDIEAVE 3894

Query 542 ANYPVQYLESMNTVLHQELIHFNRLTSVIRSSLASLRKAIRGLVVMSSDLEALGTALVQG 721

YP Y SMNTVL QEL FN L SV+RSSL +L KA++GL +MS++L+ +G AL G

Sbjct 3895 RRYPQDYYNSMNTVLAQELGRFNTLLSVVRSSLQNLGKAVKGLALMSAELDGIGRALYDG 3954

Query 722 VRPALWMKRSFPSLKPLGSYVSDLLARLAFFQGWLDNGVPTHFWISGFFFTQAFLTGSSQ 901

PA W+K+SFPSLKPLG+YV ++L R+AFFQ W+++G PT +WISGFFFTQAFLTG+ Q

Sbjct 3955 KVPAAWLKKSFPSLKPLGAYVKEVLERVAFFQSWVEDGAPTVYWISGFFFTQAFLTGAKQ 4014

Query 902 NYARANAIPIDHLGFDMHVLPANHDCSVAPQEGVYVHGIFLEGARFDESSAVLGESEPKV 1081

NYAR IPIDH+ FD V D P++GVY G+FLEG R+ L ESEPKV

Sbjct 4015 NYARKCRIPIDHIDFDFEVRDGAGDVDAPPEDGVYCAGLFLEGCRWSSDLHELDESEPKV 4074

Query 1082 LFTKLPSLWLRPQREADIADRAHYLXP 1162

LFT LP +W+ P+ A + HYL P

Sbjct 4075 LFTPLPPIWMVPREIAKFSSFPHYLCP 4101

Score = 62.0 bits (149), Expect(2) = 7e-130

Identities = 29/51 (56%), Positives = 37/51 (72%), Gaps = 2/51 (3%)

Frame = +3

Query 1161 PLYKTSDRRGTLSTTGHSTNFVMFLKLPRLEEQPQEHWVKRGVAALCELDD 1313

P+YKT++RRG LSTTGHSTNFV+ +KL + HW KRGVA + L+D

Sbjct 4101 PMYKTTERRGVLSTTGHSTNFVLDVKL--ASSKDPAHWTKRGVALITSLND 4149

Blastx e01656.1 against *C. reinhardtii*

>ref|XP_001700495.1| UniGene infoGene info dynein heavy chain 9 [Chlamydomonas reinhardtii]

gb|EDO98050.1| Gene info dynein heavy chain 9 [Chlamydomonas reinhardtii]

Length=2612

GENE ID: 5726066 DHC9 | dynein heavy chain 9 [Chlamydomonas reinhardtii]

(10 or fewer PubMed links)

Score = 85.1 bits (209), Expect = 3e-17

Identities = 43/109 (39%), Positives = 67/109 (61%), Gaps = 1/109 (0%)

Frame = +3

Query 114 HKITQMRKAKAGPKYLNQLLREVSTFLCYEATADLEMEPCAVTSRLGEEKGQRLANR-VA 290

HK+T R + +L+RE+S L YEAT DL ME A+ + + + LA + +

Sbjct 2500 HKVTLARSEETTTDNFRRLVREISQLLTYEATRDLPMETIAIKTPIAPTQSPVLAGKKLC 2559

Query 291 IVPVMRGGLGMLEAMTEMVCNARVYHLGIYRDKKSLMPVEYYNKLPKRV 437

+V ++R G G L+ M E++ ARV H+G+YRD ++L PVEYY K+P+ +

Sbjct 2560 LVSILRAGNGFLDGMLELLPAARVGHIGLYRDPETLEPVEYYFKMPEDI 2608

Blastx e01656.2 against *C. reinhardtii*

>ref|XP_001693503.1| UniGene infoGene info predicted protein [Chlamydomonas reinhardtii]

gb|EDP08757.1| Gene info predicted protein [Chlamydomonas reinhardtii]

Length=399

GENE ID: 5719196 CHLREDRAFT_77471 | hypothetical protein

[Chlamydomonas reinhardtii] (Over 10 PubMed links)

Score = 41.2 bits (95), Expect = 3e-04

Identities = 28/80 (35%), Positives = 40/80 (50%), Gaps = 7/80 (8%)

Frame = +3

Query 123 SGATAIAAIDILKAWGSTADFKLRIKFVCIGASQQAVDNISSAHPDVPIHVGLIDRLHDP 302

+G TA+ AI ILK G D RI F+ I A+ + V + S+ P V + +D D

Sbjct 325 TGGTAVKAIQILKDRGVPED---RILFLTIIAAPEGVHRVCSSFPGVKLLTSEVDEYVDK 381

Query 303 DGAPLAPILPGLGDIGDRLF 362

+ ++PG G GDR F

Sbjct 382 N----YMLVPGAGSYGDRYF 397

Clustal align of GS01392 consensus and e00124.1 nucleotide sequences

CLUSTAL W (1.83) multiple sequence alignment

GS01392consensus CCAGGAGCGGCGGAAGTTCGGTGCGCTCGGCTGGAACATCCCGTACGAGTTCAACGAGAG

e00124.1 -----------------ACGGCCCGCTCGGCTGGAACATCCCGTACGAGTTCAACGAGAG

*** *************************************

GS01392consensus CGACTTGCGCATCTCGGTCCGGCAGCTGCGGCTCTTCTTGACCATGTTCGACGACGTGCC

e00124.1 CGACCTGCGCATCTCTGTGCGACAGCTGCAGATGTTCCTCGACAGCTATGACGAGACACC

**** ********** ** ** ******* * * *** * ** * ***** **

GS01392consensus TTTCGAAGCGTTGCGCTACTGCTTTGGCGAGGCCAACTATGGCGGCCGCGTGACCGACGA

e00124.1 GCTTGCAGCGCTCAACTACCTCACCGCCGAGTGCAACTATGGCGGCAGGGTGACGGATGA

* * **** * **** * * **** ************* * ***** ** **

GS01392consensus CAAGGACCGGCG-ATGCCTG--AGCGCGCTCCTCGCGGAGCCAATCTCGGCGGCTGCGAT

e00124.1 CAAGGACCGCCGCACGCTCGTCACAGCGGTGCT-GAACATCTACTGCCCGCAGATTC--T

********* ** * ** * * *** * ** * * * * * * ** * * * *

GS01392consensus CGAGCCCGGCTTCACCTTCTCATCGGACGGGGTGTAC-TG--TCAACCCGGGGCG---GC

e00124.1 GGAGGATGGGTACCGCCTCTCGGCGTCGGGCATGTACGTGGTTCCGACCGATGCGCTTGA

*** ** * * * **** ** ** ***** ** ** *** *** *

GS01392consensus CACGCACGAGGGCTACCTTGAATCGATCCGCGC----GCTTCCTCTCACGCAGCTGCCCA

e00124.1 CTCGCACGAGGCGACGGTCGAGTACATTCG-GCAGTGGCCGCCTGTCGCCAAAC---CAG

* ********* * ** * ** ** ** ** *** ** * * * *

GS01392consensus CGGCCTTTGGCCTGCACGAGAACGCCGATATCACCAAGGATCTCAAGGAGACACGCGAGC

e00124.1 AGGTGTTTGGGTTGCACGAGAATGCCGACATCACAAAGGATCTGGGCGAGGTGGACCTGA

** ***** ********** ***** ***** ******** *** * *

GS01392consensus TCTTCGACGCCGTGCTCTCGACCCAGGCTCGCGCCGGCGCTGGCGGCGGGGGCGGTGGCG

e00124.1 TGCTCAAGACCATGCTACTCACCCAGGCGCAGAGCGGTGGCGGTGGCGGTGGCGGTGGCA

* ** * ** **** ******** * *** * ** ***** *********

GS01392consensus AG---------GATGTGCTCGACAAGATCGCCGAGGGTATCGCTTCGAAGCTCCCTCCCG

e00124.1 AGTCGACCGATGACGTGGTCGCCGAAATCTCCGGCGAGATCCTGGGCAAGCTCCCGAGCA

** ** *** *** * * *** *** * *** ******** *

GS01392consensus CCTTCGACCTCGACGTGGCCGGAGCCAACTACCCCGTACAGTACCTCGAGTCGATGAACA

e00124.1 ACTTTGACATGGAGGTTGCGGCGCGGCGGTACCCTGTGGTATACTCCGAGTCGATGAACA

*** *** * ** ** ** * ***** ** *** **************

GS01392consensus CGGTGCTGCACCAGGAACTGATTCACTTCAACCGGCTGACGTCGGTCATCCGGTCGTCGC

e00124.1 CCGTGCTAAGACAGGAACTGCAGCGCTTCAACAAGCTCACCTCCAAGATCAGGAGCTCGC

* ***** ********* * ******* *** ** ** *** ** ****

GS01392consensus TCGCCTCGCTGCGCAAGGCAATCAGGGGTCTCGTGGTCATGAGCTCCGACCTCGAGGCGC

e00124.1 TTCTCGAACTGGGCAAAGCGCTCAAGGGGCTCGTCGTGATGAGCGCCGACCTCGAGTCGA

* * *** **** ** *** *** ***** ** ****** *********** **

GS01392consensus T---GGGCACCGCCCTTGTCCAGGGGGTGCGGCCGGCGCTCTGGATGAAGCGCTCCTTCC

e00124.1 TTGCGAGCGCCATGTTTGTCAACCAGGTGC---CGGAGTCGTGGGCCAAGGTGTCATATC

* * ** ** ***** * ***** *** * *** *** ** * *

GS01392consensus CCTCGCTGAAGCCGCTGGGGAGCTACGTGAGCGACCTGCTCGCCCGCTTAGCCTTCTTCC

e00124.1 CCTCGCTCAAGCCCCTCTCTGCCTACGTCACGGAGCTCCTCGAACGGCTTGAGTTCTTTT

******* ***** ** ****** * ** ** **** ** * * *****

GS01392consensus AGGGCTGGCTCGACAACGGCGTGCCGACCCACTTTTGGATCTCCGGCTTTTTCTTCACAC

e00124.1 CGACGTGGCTTGAGGAGGGCAACCCCGTCTGCTATCCCATGCCGCACTTCTTCTTCGTGC

* ***** ** * *** ** * ** * ** * *** ****** *

GS01392consensus AGGCGTTCCTCACGGGCTCCAGCCAAAACTACGCGAGGGCCAACGCGATCCCGATCGACC

e00124.1 AGGCCTTTATGACTGGCGCGCTGCAAAACTACGCCCGAAAATACACGCTGCCCATCGACA

**** ** * ** *** * *********** * ** ** * ** ******

GS01392consensus ACCTCGGCTTTGACATGCACGTGCTCCCGGCGAACCACGACTGCTCCGTCGCGCCGCAGG

e00124.1 CCGTCGAGTTTGACTTTCAC-TTTTTCTGGGAGGCTCCAACGGC-----CAAGCCTGAGG

* *** ****** * *** * * * ** * * ** ** * *** ***

GS01392consensus AGGGCGTGTACGTGCACGGGATCTTCCTGGAGGGCGCGCGCTTCGACGAGAGCAGCGCGG

e00124.1 ACGGCGTGCACACGAGCGGTCTCTTCGTCGAGGGTGCGCGGATGGGCGACGAGAAGGTCC

* ****** ** * *** ***** * ***** ***** * * *** * *

GS01392consensus TGCTCGGCGAGTCGGAACCTAAGGTACTCTTCACCAAACTCCCCTCCCTCTGGCTGCGAC

e00124.1 AGCTCGAGGAGTCGCAACCCAAGGTGCTATTCAGCCCAATGACTTACGTCCAGCTGCTGC

***** ****** **** ***** ** **** * * * * * * ** ***** *

GS01392consensus CGCAACGGGAGGCTGACATCGCCGACCGGGCGCACTACCTCTGCCCCCTCTACAAGACGT

e00124.1 CAGTGCAGTCGGACAAGCTGTCCACCTACCCCCACTATGAGTGCCCGGTCTACCGGACGA

* * * ** * * ** * * ***** ***** ***** ****

GS01392consensus CCGACCGGCGCGGCACGCTCTCGACGACGGGCCATTCGACCAACTTTGTCATGTTCCTCA

e00124.1 CAGCGCGGCGCGGGACGCTCTCCACCACCGGCCATTCCACCAACTTCGTCATGTTCATGC

* * ******** ******** ** ** ******** ******** ********* *

GS01392consensus AGCTGCCGCGGTTAGAGGAGCAGCCGCAGGAACACTGGGTGAAGAGGGGGGTGGCGGCGC

e00124.1 GGCTGCCG------ACGGATCAGCCGTCATCACACTGGATAGCCCGTGGAGTGGCCCTGA

******* *** ****** ******* * * ** ***** *

GS01392consensus TGTGTGAGCTGGACGACTAAGTACGTGTGCGGGCGGGCGGTTTGTACTCGAGTACCCCTG

e00124.1 TCAACAGTCTGGCCACTTGACAATTTTCTAAGGCGAGGTGAGAG-ACT---GTGCTCGTA

* **** * * * * * **** * * * *** ** * * *

GS01392consensus CTGTGGCGGGAGAGAGGTAGGGGAAATAATCTGCGAACGAGCTCCAAGTG

e00124.1 CGTTTACAGGTTTGACGTGTAAATTACAATGCGCCATAGAGAGC------

* * * ** ** ** * *** ** * *** *

GS01392 and GS00124 appear to be distinct.

Blastx e02889.1 against *C. reinhardtii*

>ref|XP_001696428.1| UniGene infoGene info cytoplasmic dynein 1b heavy chain [Chlamydomonas reinhardtii]

gb|EDP08405.1| Gene info cytoplasmic dynein 1b heavy chain [Chlamydomonas reinhardtii]

Length=4333

GENE ID: 5722214 DHC1b | cytoplasmic dynein 1b heavy chain

[Chlamydomonas reinhardtii] (Over 10 PubMed links)

Score = 78.6 bits (192), Expect = 2e-15

Identities = 51/136 (37%), Positives = 77/136 (56%), Gaps = 8/136 (5%)

Frame = +2

Query 269 GFFLTALRQQTARSTKTPMDALELCCGLASADLR---GASLAVVVSGLALQGATCSTSAG 439

G FL ALRQQ+AR+ MD L+ +A L+ G + ++ GL +QGAT S

Sbjct 4200 GTFLNALRQQSARTLGCSMDMLKAVTSWETAKLKAAAGGAPVALLGGLIMQGATFDGSR- 4258

Query 440 LAPLDADAPTRSSMPDLHLAWVLAGSPPPYDTKRSALVPLYADGERTTLLCDVRLPCSGS 619

L+P+ A+AP ++P + +AW+ SP Y + A PLY +R+ LL V+LP SG

Sbjct 4259 LSPVAAEAPAFRAVPAMSMAWLHKDSPMAYASYMEA--PLYMTSDRSKLLARVQLPVSGP 4316

Query 620 DAQ--WLQAGVACFLA 661

+ W+ AG++ FL+

Sbjct 4317 EEMDGWVLAGLSLFLS 4332

Blastx e02889.2 against *C. reinhardtii*

>ref|XP_001696428.1| UniGene infoGene info cytoplasmic dynein 1b heavy chain [Chlamydomonas reinhardtii]

gb|EDP08405.1| Gene info cytoplasmic dynein 1b heavy chain [Chlamydomonas reinhardtii]

Length=4333

GENE ID: 5722214 DHC1b | cytoplasmic dynein 1b heavy chain

[Chlamydomonas reinhardtii] (Over 10 PubMed links)

Score = 70.5 bits (171), Expect = 3e-13

Identities = 62/197 (31%), Positives = 87/197 (44%), Gaps = 53/197 (26%)

Frame = +1

Query 1 IPPAWAKEWEGSEEPAEWMRQVTSRAIAIEA-WHGAAQGGG-------llsrplrlhell 156

IP +W WEG E P ++ R V ++A+AIE W Q GG + PL L +

Sbjct 4138 IPGSWDAAWEGPEAPMDYCRAVVAKALAIEGHWARCQQPGGGGLLDGSGGAGPLELSSVF 4197

Query 157 hpGFFLTALRQQTARSTKTPMDALD----------------------------------- 231

HPG FL ALRQQ+AR+ MD L

Sbjct 4198 HPGTFLNALRQQSARTLGCSMDMLKAVTSWETAKLKAAAGGAPVALLGGLIMQGATFDGS 4257

Query 232 ------ADAPTRSSMPDLHLAWVLAGSPPPYDTKRSALVPLYADGERTTLLCDVRLPCSG 393

A+AP ++P + +AW+ SP Y + A PLY +R+ LL V+LP SG

Sbjct 4258 RLSPVAAEAPAFRAVPAMSMAWLHKDSPMAYASYMEA--PLYMTSDRSKLLARVQLPVSG 4315

Query 394 SDAQ--WLQAGVACFLA 438

+ W+ AG++ FL+

Sbjct 4316 PEEMDGWVLAGLSLFLS 4332

Blastx GS03135 against *C. reinhardtiii*

>ref|XP_001696428.1| UniGene infoGene info cytoplasmic dynein 1b heavy chain [Chlamydomonas reinhardtii]

gb|EDP08405.1| Gene info cytoplasmic dynein 1b heavy chain [Chlamydomonas reinhardtii]

Length=4333

GENE ID: 5722214 DHC1b | cytoplasmic dynein 1b heavy chain

[Chlamydomonas reinhardtii] (Over 10 PubMed links)

Score = 228 bits (581), Expect = 2e-60

Identities = 120/262 (45%), Positives = 172/262 (65%), Gaps = 9/262 (3%)

Frame = +2

Query 2 EKELSALEPHPDFRLWLTTEQHAAFPPIMLQQSLKVTFEAPPGMQKNLSRSFESSLSAEI 181

EKEL L+ H +FR++LT+E H FP +L+ SLKVTFEAPPGM+KNL R++E+ SAE

Sbjct 3805 EKELLMLQKHDNFRIFLTSEPHPKFPSTLLEMSLKVTFEAPPGMKKNLQRTYEA-WSAEY 3863

Query 182 VESGSPQRAQLLLVIAWFHAVLQERRSFLPQGWTKFYEFSPSDLRTAIDICDSAGGVVKG 361

+ SG P RAQLL V+AWFHAV+QERR+++PQGWTKFYEFS +DLR+ +D+ A

Sbjct 3864 LASGPPIRAQLLFVLAWFHAVVQERRTYIPQGWTKFYEFSFADLRSGMDVITLATRAGTA 3923

Query 362 QPDWAAIHGLLSLAVYGGRVDNLHDERLLHSFLQQYFSRAML--SGGRL----AQGVAIP 523

P W + GLL A+YGGR+DN D ++L +FL++ FS + +GG++ V +P

Sbjct 3924 -PQWPLLLGLLDDAIYGGRLDNPFDSQVLLTFLRRLFSAETVGAAGGKVRPLPGSKVVVP 3982

Query 524 TSNRHADYLRAIESIPPIDSPTLFGLPANAGMAVQKRAATAVRASLRELDRDEAESTSSF 703

T+N ADY+ I ++P +D+P LF +P N Q+ + V + L+ + A++ F

Sbjct 3983 TTNHRADYVSIISALPDVDTPGLFCMPDNIDRTAQQVNSARVISQLKAMSL-RADAAGGF 4041

Query 704 DREVWTHALSPVLSLWQKLLSG 769

+R W L P+L LW +L+SG

Sbjct 4042 NRAQWQAQLGPLLRLWDQLMSG 4063
